# Direct catalytic cross-coupling of alkenyllithium compounds†
†Electronic supplementary information (ESI) available. See DOI: 10.1039/c4sc03117b


**DOI:** 10.1039/c4sc03117b

**Published:** 2014-11-28

**Authors:** Valentín Hornillos, Massimo Giannerini, Carlos Vila, Martín Fañanás-Mastral, Ben L. Feringa

**Affiliations:** a Stratingh Institute for Chemistry , University of Groningen , Nijenborgh 4 , 9747 AG Groningen , The Netherlands . Email: b.l.feringa@rug.nl ; Fax: +31 50 363 4278 ; Tel: +31 50 3634296

## Abstract

A catalytic method for the direct cross-coupling of alkenyllithium reagents with aryl and alkenyl halides is described. The use of a catalyst comprising Pd_2_(dba)_3_/XPhos allows for the stereoselective preparation of a wide variety of substituted alkenes in high yields under mild conditions. In addition (1-ethoxyvinyl)lithium can be efficiently converted into substituted vinyl ethers which, after hydrolysis, give readily access to the corresponding methyl ketones in a one pot procedure.

## Abstract 2

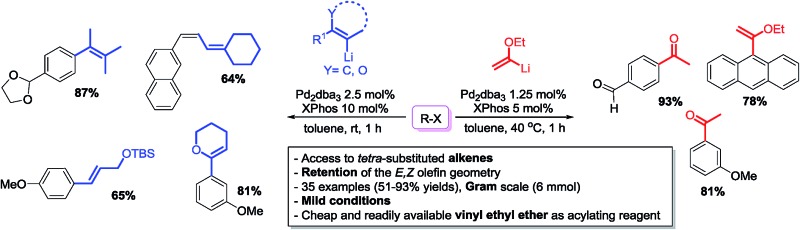
The direct cross-coupling of alkenyllithium reagents with aryl and alkenyl halides is described. The use of a catalyst comprising Pd_2_(dba)_3_/XPhos allows for the stereoselective preparation of a wide variety of substituted alkenes in high yields under mild conditions.

## Introduction

Palladium-catalysed cross-coupling of organometallic reagents with organic halides represents one of the most powerful methods for the construction of carbon–carbon bonds.^1^ Among these, reactions employing alkenylmetal reagents remain indispensable tools to access a broad range of highly substituted olefins and polyenes.^2^ With a variety of structurally diverse, functionalized organic halides available, the challenge of this transformation often remains the choice of the alkenyl nucleophile. Tin^3^ and boron^4^ reagents are frequently used for these coupling reactions. However, the high toxicity of organostannanes makes the Stille cross-coupling often less desirable. On the other hand, alkenyl boronic acids are prone to rapid protodeboronation or polymerization so requiring large excess of the coupling partner. Elegant solutions have been found *via* the conversion into more stable derivates such as boronic esters, MIDA boronates^5^ and trifluoroborates.^6^ Organosilicon compounds feature high stability and low toxicity although the use of fluoride anion for the activation is generally needed.^7^ Nonetheless, non-fluoride activators have recently been introduced to enhance the utility of these compounds.^8^,2d Alkenyl reagents for the Negishi cross-coupling (Zn, Al and Zr), have also been used in Pd-catalysed alkenylation reactions with a broad scope for the synthesis of dienes and polyenes.^9^ These alkenyl intermediates are routinely prepared by elementometalation^9^ from alkynes or transmetallation from the corresponding organolithium reagents, as is the case for boron, tin and silicon reagents. In contrast, the direct use of alkenyllithium compounds^10^ have, to the best of our knowledge, not been reported in Pd-catalysed cross-coupling reactions. Alkenyllithium reagents are easily accessible by lithium–halogen exchange or *via* direct metallation of the corresponding olefins without requiring purification prior to use.^11^ Their high reactivity has largely prohibited the use of these reagents in cross-coupling reactions due to the lack of selectivity. Pioneering studies by Murahashi and co-workers on the use of aryl and alkyllithium reagents in catalytic cross-coupling reactions revealed the limitations associated with their high reactivity.^12^,^13^ However, if controlled, this reactivity might be advantageous in facilitating the transmetallation with Pd allowing for milder reaction conditions which are beneficial to preserve the geometry of the alkene. In addition, their use would also drastically reduce the amount of byproducts generated with the light and non-toxic lithium halide being the only stoichiometric waste. Therefore, the development of a general method for the use of alkenyllithium reagents in catalytic cross-coupling reactions is highly desirable.

Recently, our group reported the direct palladium catalysed cross-coupling of alkyl and (hetero)aryl lithium reagents with aryl and alkenyl (pseudo)halides, providing high yields and selectivity.^14^,^15^ The choice of the proper combination of catalyst and reaction conditions allows for efficient transmetallation, prevents the notorious lithium halogen exchange and homocoupling reactions and gives rise to the corresponding cross-coupled products in high yields. However, the use of alkenyllithium reagents in this transformation remains elusive.

**Scheme 1:**
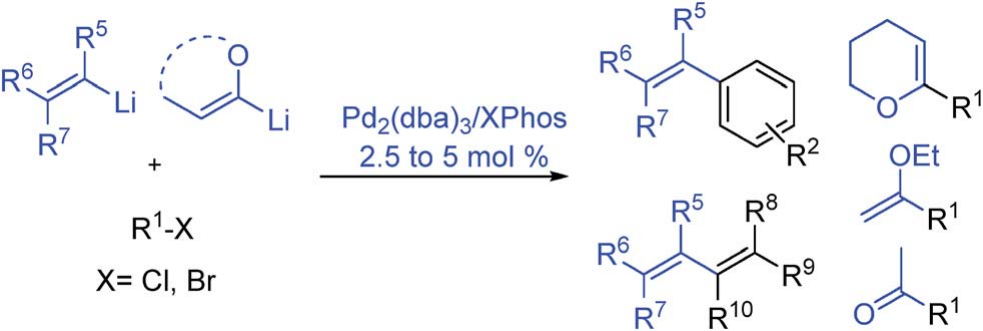
Palladium catalysed cross-coupling of alkenyllithium reagents.

Herein, we report a palladium-based method for the direct catalytic cross-coupling of alkenyllithium reagents and organic halides to afford substituted alkenes in high yield and isomeric purity under mild conditions in short reaction times (Scheme 1).

## Results and discussion

Our investigation started with the reaction between (3-methylbut-2-en-2-yl)lithium, readily prepared from the corresponding bromide, and 4-methoxy-bromobenzene **1a**, a reluctant arylbromide in many coupling reactions,^16^ in the presence of various Pd-based catalytic systems using toluene as solvent (Table 1).

**Table 1:** Screening of different Ligands

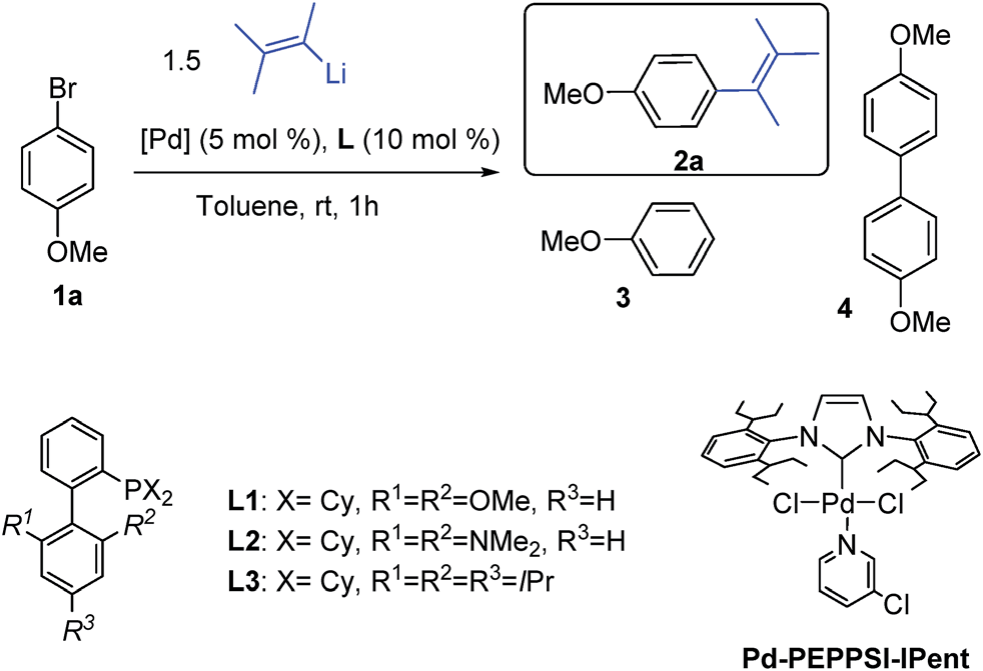				
Entry^*a*^	[Pd]	Ligand	Conv. (%)	**2a** : **3** : **4**^*b*^
1	Pd[P(*t*-Bu)_3_]_2_		Full	44 : 56 : 0
2	Pd_2_(dba)_3_	**L1**, SPhos	46	83 : 0 : 17
3	Pd_2_(dba)_3_	**L2**, CPhos	38	87 : 0 : 13
4	Pd_2_(dba)_3_	**L3**, XPhos	Full	>99 : 0 : 0
5	Pd-PEPPSI-IPent		Full	96 : 0 : 4

^*a*^Conditions: (3-methylbut-2-en-2-yl)lithium (0.40 mmol, 0.6 M in THF) was added to a solution of **1a** (0.3 mmol) and catalyst in toluene (1 mL), 1 h addition time.

^*b*^ **2a** : **3** : **4** ratios determined by GC analysis. dba = dibenzylideneacetone.

The use of Pd[P(*t*-Bu)_3_]_2_ 17 (Table 1, entry 1), a catalyst we previously disclosed to be highly successful for the cross-coupling of alkyllithium reagents with aryl bromides14a,d led to the desired product **2a** but in the presence of a large amount of dehalogenated side product **3**. We then examined the use of Buchwald biaryl phosphine ligands^18^ in combination with Pd_2_(dba)_3_ (2.5 mol%). When ligands **L1** and **L2** were used, incomplete conversion was observed, along with homocoupling side product **4** (entries 2 and 3). To our delight, the use of XPhos **L3** led to the cross-coupled product **2a**, in 1 h at r.t., with excellent selectivity (>99%), avoiding dehalogenation and inhibiting the formation of the homocoupling product (entry 4). We also found that the air stable Pd-PEPPSI-IPent catalyst, introduced by Organ,^19^ displayed good selectivity although the presence of 4% of homocoupling was observed (entry 5).

**Scheme 2:**
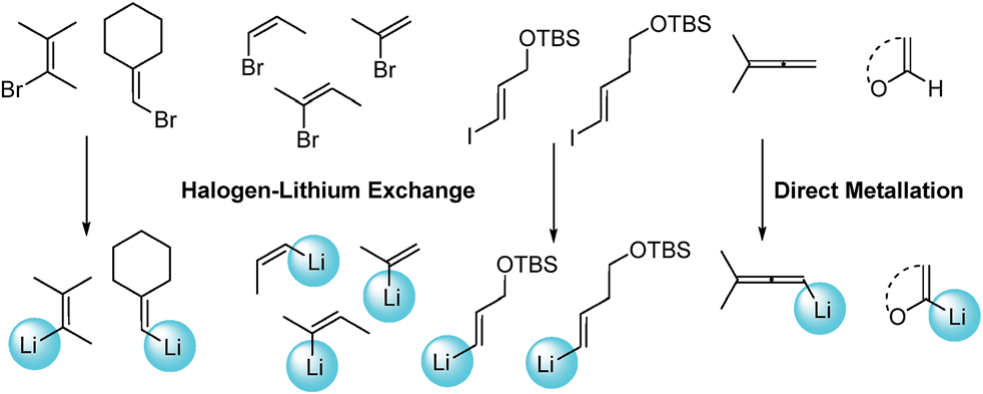
Synthesis of alkenyllithium reagents.

With Pd_2_(dba)_3_/Xphos as a highly efficient catalytic system, we set out to investigate the scope of this new reaction (Table 2)‡
‡Representative procedure: In a dry Schlenk flask Pd_2_(dba)_3_ (2.5 mol%) and XPhos (10 mol%) were dissolved in toluene (2 mL/0.3 mmol of substrate) and the solution was stirred under nitrogen atmosphere at room temperature for 5 min. The substrate (1 equiv.) was added and the solution stirred at the indicated temperature. The corresponding lithium reagent solution (1.3 equiv., 0.6 or 0.68 M, see ESI† for details) was slowly added over 1 h by the use of a syringe pump. After the addition was completed a saturated solution of aqueous NH_4_Cl was added and the mixture was extracted with Et_2_O or AcOEt. The organic phases were combined and dried with anhydrous Na_2_SO_4_. Evaporation of the solvent under reduced pressure afforded the crude product that was then purified by column chromatography.. A wide variety of alkenes were readily lithiated *via* direct metallation or by halogen–lithium exchange as shown in Scheme 2 (see ESI† for details).

**Table 2:** Pd-catalysed cross-coupling of alkenyllithium reagents with aryl and alkenyl halides^*a*^

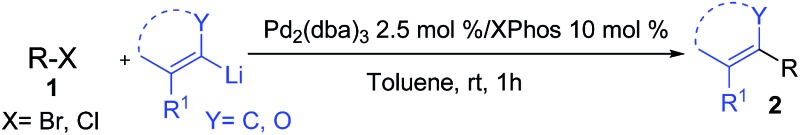
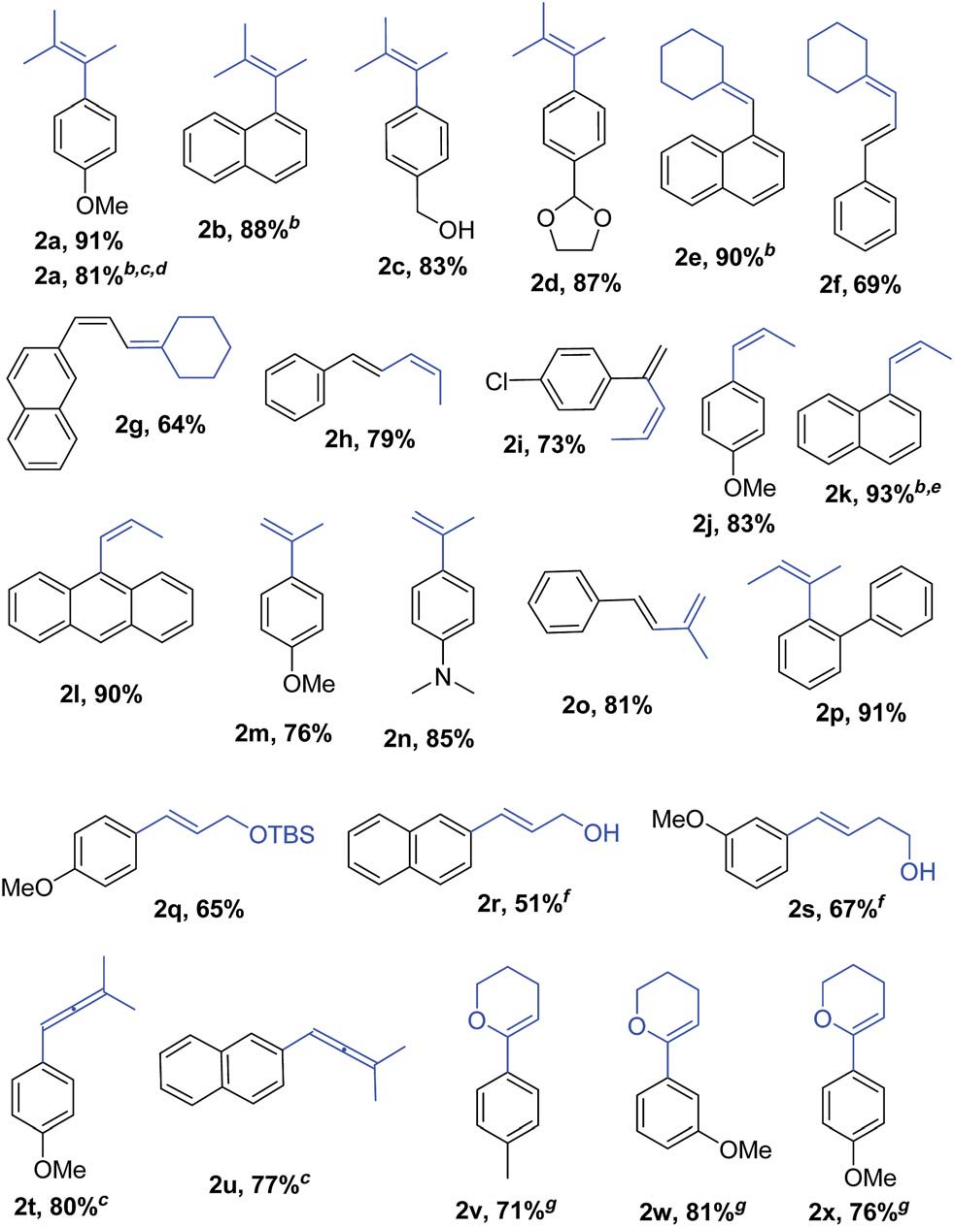

^*a*^Conditions: RLi (1.3 equiv.) was added to a solution of organic bromide (0.3 mmol) and catalyst in toluene, 1 h addition time unless otherwise noted. Selectivity **2** : **3**,**4** > 90%. Isolated yields after column chromatography. For limitations of the method see Table S3, Schemes S2 and S3.

^*b*^X = Cl.

^*c*^The reaction was performed at 40 °C.

^*d*^3.5 h addition time.

^*e*^The reaction was performed at 35 °C.

^*f*^After workup, product mixture was treated with TBAF to remove the silyl group prior to purification.

^*g*^0.9 mmol scale. Reaction performed at 60 °C.

The catalytic system proved to be also highly efficient in the reaction of (3-methylbut-2-en-2-yl)lithium with 1-chloronaphthalene **1b** at r.t. (see also ESI, Table S1†). The corresponding tetrasubstituted alkene **2b** was obtained in good yield with no trace of the 2-substituted regioisomer, indicating that benzyne intermediates *via* 1,2-elimination are not formed. To further explore the effectiveness of this catalytic systems we studied the more reluctant coupling partner 4-methoxy-chlorobenzene.14*b* Unfortunately, low conversion was found in the reaction at room temperature. However, the use of slightly higher temperatures (40 °C) and longer addition times (3.5 h) of the organolithium reagent allowed full conversion with high selectivity (see ESI, Table S2†). Substrate **1c**, bearing an unprotected hydroxyl group, could also be coupled with this organolithium reagent (2.2 equiv.). The use of acetal-protected aldehyde **1d** was also tolerated without cleavage of the protecting group. The cross-coupling of (cyclohexylidenemethyl)lithium and 1-chloronaphthalene proceeds efficiently giving alkene **2e** in good yield. It is important to note that (*E*) and (*Z*) alkenyl bromides **1f** and **1g** reacted under the optimized conditions to give conjugated dienes **2f** and **2g** in good yield and with no presence of Fritsh–Butlenberg–Wiechell type rearrangement side products.^20^ In addition, both (*E*) and (*Z*)-olefins were coupled to form dienes **2f–2h** with retention of stereochemistry. Illustrative is the use of (*Z*)-propenyllithium, obtained by treatment of (*Z*)-1-bromopropene with elemental lithium, which was smoothly coupled with retention of the (*Z*)-configuration, with a variety of alkenyl and aryl bromides providing the corresponding dienes **2h**, **2i** and alkenes **2j**, **2k** and **2l** in good yields. Synthesis of diene **2h** illustrates that both the alkenyl nucleophile and electrophile retain their geometrical configuration as confirmed by ^1^H NMR.^21^ In addition, vinyl bromide **1i** reacted chemoselectively, leaving the chloride intact and available for further functionalization. It is interesting to note that (*Z*)-propenyllithium compared with the more hindered (3-methylbut-2-en-2-yl)lithium, required higher temperature in the coupling with 1-chloronaphthalene. This result could be due to a faster reductive elimination taking place from the more strained Pd(ii) intermediates.^22^ This result is in accordance with similar observation using less hindered 2-chloronaphthalene. (see ESI, Scheme S1†).

2-Propenyllithium underwent cross-coupling reaction with 4-methoxy-bromobenzene and the more electron rich 4-bromo-*N*,*N*-dimethylaniline providing styrenes **2m** and **2n** in good yield without the need to increase temperature or reaction time. Retention of the olefin configuration was also observed in the reaction of this organolithium reagent with (*E*)-bromostyrene **2o**. Moreover, a sterically hindered *ortho*-substituted arylbromide such as 2-bromo-1,1′-biphenyl **1p** could be coupled in high yield and without loss of selectivity. Remarkably, alkenyllithium reagents bearing a protected alcohol functionality could also be used, providing the corresponding allylic TBS-protected alcohol **2q**, allylic alcohol **2r** and homoallylic alcohol **2s** in fair to good yields with retention of the (*E*)-configuration. Interestingly, compound **2q** is an intermediate in the synthesis of (–)-cytoxazone, a Novel Cytokine Modulator Isolated from *Streptomyces* sp.^23^

We envisioned extension of this coupling to allenyllithium^24^ compounds, providing a synthesis of substituted allenes.^25^ Under the optimized reaction conditions the coupling between (3-methylbuta-1,2-dienyl)lithium, readily available by direct metallation of the corresponding allene, and both 4-methoxy-bromobenzene and 2-chloronaphthalene proceeded in good yields and high regioselectivity and with less than 5% of regioisomeric alkyne arising from 1,3-lithium shift of the organolithium reagent.^26^

Next the use of functionalized (α-alkoxyvinyl)lithium reagents in this new cross-coupling transformation was investigated. An illustrative example is the reaction of (3,4-dihydro-2*H*-pyran-6-yl)lithium, obtained by direct metallation of commercially available dihydropyran with *t*BuLi.27*a* To our delight, reaction with *p*-methyl- and *p*- and *m*-methoxy-substituted bromoarenes provided compounds **2v**, **2w** and **2x** in high yields and excellent selectivity (Table 2). Due to the reduced reactivity of this organometallic reagent, a slightly higher temperature was required (60 °C, 1 h). Nonetheless, established methods for the cross-coupling of glycal metal reagents require severe reaction conditions, long reaction times or they only were examined with the corresponding aryl iodides.27b,c

Encouraged by these results, we studied other α-alkoxyvinyl precursor such as the cheap and simple ethyl vinyl ether (Table 3). The use of the corresponding lithium reagent, readily obtained by direct lithiation, in the Pd-catalysed cross-coupling reaction could be used as a masked acetylating agent giving, after hydrolysis, the corresponding aryl methyl ketones.27b,^28^ This class of carbonyl derivates serves as versatile synthetic building blocks and are intermediates in the synthesis of drug candidates, fragrances and heterocycles.^29^ We performed this one pot transformation on larger scale (6 mmol), employing 2.5 mol% of catalyst to illustrate the synthetic utility of the method. As shown in Table 3, the method tolerates a variety of functional groups including acetals, ethers, amines alcohols and phenols. Electron rich substrates bearing amine, methyl and methoxy substituents underwent the expected coupling reaction at 40 °C in 2.5 h to give the corresponding ketones **6a–6c** in good yields. Remarkably, bromofluorene was successfully employed, despite the acidity of the benzylic protons (p*K*_a_ = 22). Although the formation of **6e** demonstrates that hindered substrates are tolerated, we further validated this by coupling 9-bromoanthracene to give **5f** in 78% yield. Facile multiple coupling is illustrated with the two fold acetylation of 4,4′-bis-bromobiphenyl, providing **6g** in 76% yield. β-Bromo-styrene was coupled in moderate yield, with retention of the configuration. Acetal-protected *p*-bromobenzaldehyde was also tolerated in the reaction affording, after subsequent hydrolysis, 4-acetylbenzaldehyde **6i** in excellent yield. Alcohols and phenols as Mg alkoxides are tolerated in this method to afford **6j** and **6k**. It should be noted that same of the compounds obtained such as **6a**, **6d**, **6e**, **6i**, or **6j** would be challenging to synthesize through standard Friedel–Crafts acylation chemistry.^30^

**Table 3:** Pd-catalysed cross-coupling of (1-ethoxyvinyl)lithium^*a*^

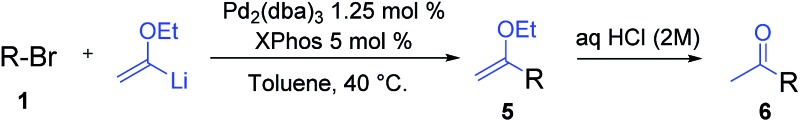
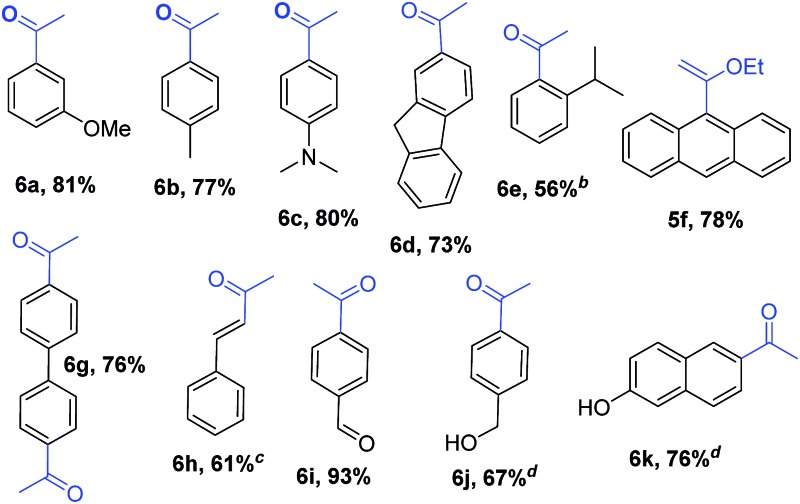

^*a*^Conditions: 3.0–6.0 mmol scale reaction. Aryl bromide (1 equiv.), (1-ethoxyvinyl)lithium (1.5 equiv., 0.60 M in THF). 2.5 h addition time. Toluene at 40 °C. Selectivity >90%. Yield values refer to isolated yields after purification.

^*b*^70% conversion.

^*c*^80% selectivity.

^*d*^iPrMgCl (1.0 equiv., 2 M in Et_2_O) was added over 5 min prior to the organolithium.

## Conclusions

In summary, a fast and selective method has been developed for the Pd-catalysed alkenylation of aryl and alkenylhalides using organolithium reagents. This transformation leads to highly substituted alkenes under mild conditions and shows tolerance to various functional groups. Moreover, we describe the one pot synthesis of aryl methyl ketones involving coupling of lithiated alkenyl ethers.

## Supplementary Material

Supplementary information
