# Supplementary material for: Direct catalytic cross-coupling of alkenyllithium compounds
Source: Chem Sci. 2014 Nov 28;6(2):1394–8. doi: 10.1039/c4sc03117b (PMC5811103; doi:10.1039/c4sc03117b)
Supplement: Supplementary file 1 [file SC-006-C4SC03117B-s001.pdf]

Electronic Supplementary Material (ESI) for Chemical Science.
This journal is © The Royal Society of Chemistry 2014

Supplementary Information

Direct Catalytic Cross-Coupling of Alkenyllithium Compounds

Valentín Hornillos, Massimo Giannerini, Carlos Vila, Martín Fañanás-Mastral and Ben L. Feringa*

Correspondence to: b.l.feringa@rug.nl

Stratingh Institute for Chemistry, University of Groningen, Nijenborgh 4, 9747 AG, Groningen, The Netherlands.

Table of Contents

**Table of Contents**	**S2**
**General Methods**	**S3**
**Additional Data**	**S4**
**General Procedures for the Cross-Coupling of alkenyllithium Reagents**	**S7**
**Preparation of alkenyllithium Reagents**	**S7**
**Data of Compounds 2a-2u, 6a-6k**	**S9**
**^{1}H and ^{13}C NMR spectra**	**S22**

General methods:

All reactions were carried out under a nitrogen atmosphere using oven dried glassware and using standard Schlenk techniques. THF and toluene were dried and distilled over sodium. $Pd_2(dba)_3$, SPhos, XPhos, DavePhos, CPhos, Qphos, PCy_3, Pd-PEPPSI-*I*Pr and Pd-PEPPSI-*I*Pent were purchased from Aldrich and used without further purification. *t*BuLi (1.7 M in pentane), Lithium granular (4-10 mesh particle size, high sodium, 99%), DIBAL-H (1.0 M in THF), $ZrCp_2Cl_2$, *tert*-butyldimethyl(2-propynyloxy)silane, (but-3-yn-1-yloxy)(*tert*-butyl)dimethylsilane, iodine and the compounds used as precursor for the preparation of lithium reagents, namely 2-bromo-3-methyl-2-butene, bromomethylenecyclohexane, (1*Z*)-1-bromo-1-propene, 2-bromo-1-propene, (*E*)-2-bromo-2-butene, 3-methyl-1,2-butadiene, 3,4-dihydro-2*H*-pyran and 1-ethoxyethylene were purchased from Aldrich. All the aryl- and vinylhalides were commercially available and were purchased from Aldrich, TCI Europe N.V. and Acros Organics. Organolithium reagents were prepared according to described procedures (*vide infra*).

Chromatography: Merck silica gel type 9385 230-400 mesh, TLC: Merck silica gel 60, 0.25 mm. Components were visualized by UV and cerium/molybdenum or potassium permanganate staining. Progress and conversion of the reaction were determined by GC-MS (GC, HP6890: MS HP5973) with an HP1 or HP5 column (Agilent Technologies, Palo Alto, CA). Mass spectra were recorded on an AEI-MS-902 mass spectrometer (EI+) or a LTQ Orbitrap XL (ESI+). ^{1}H- and ^{13}C-NMR were recorded on a Varian AMX400 (400 and 100.59 MHz, respectively) using $CDCl_3$ as solvent. Chemical shift values are reported in ppm with the solvent resonance as the internal standard ($CHCl_3$: δ 7.26 for ^{1}H, δ 77.0 for ^{13}C). Data are reported as follows: chemical shifts, multiplicity (s = singlet, d = doublet, t = triplet, q = quartet, br = broad, m = multiplet), coupling constants (Hz), and integration. Carbon assignments are based on APT ^{13}C-NMR experiments.

Additional Data:

Table S1. Optimization data of the reaction between (3-methylbut-2-en-2-yl)lithium and 1-chloronaphthalene[a]

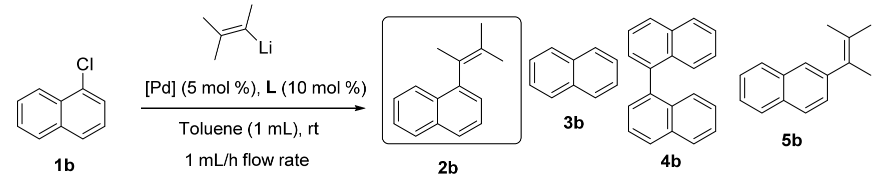

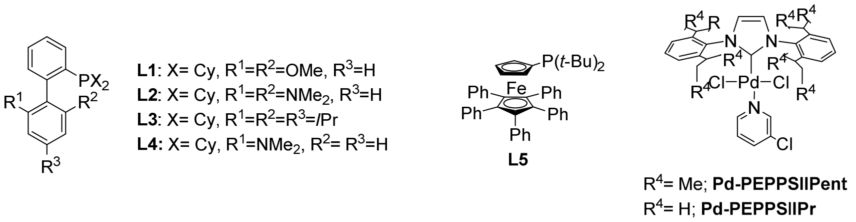

[Pd]	Ligand	2b%	1b%	3b%	4b	5b
$Pd_2(dba)_3$	**L3**, XPhos	99	0	0	0	0
$Pd_2(dba)_3$	**L1**, SPhos	47	35	2	12	4
$Pd_2(dba)_3$	**L2**, Cphos	79	9	0	12	0
$Pd_2(dba)_3$	**L4**, DavePhos	20	63	3	11	3
$Pd_2(dba)_3$	PCy_3	64	24	0	6	6
$Pd_2(dba)_3$	**L5**, QPhos	14	60	0	1	19
Pd[P(*t*-Bu)$_3$]$_2$		21	38	0	0	24
Pd-Peppsi-IPr		71	23	0	1	5
Pd-Peppsi-IPent		99	0	0	0	0
No catalyst		19	52	0	0	30

[a]Conditions: (3-methylbut-2-en-2-yl)lithium (0.45 mmol, 0.6 M in THF) was added to a solution of **1b** (0.3 mmol) in toluene (1 mL), flow rate = 1.0 mL/h. [b]**2b:3b:4b:5b** ratios determined by GC analysis. dba = dibenzylideneacetone.

Table S2. Optimization data of the reaction between (3-methylbut-2-en-2-yl)lithium and 4-methoxy-chlorobenzene[a]

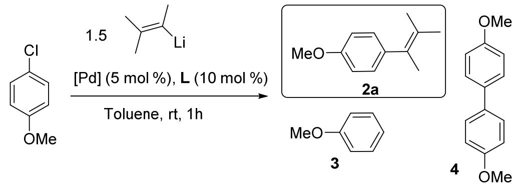

Entry[a]	[Pd]	Ligand	Temp.	flow rate mL/h	Conv. (%)	2a:3:4[b]
1	**$Pd_2(dba)_3$**	L3, XPhos	rt	1	10	>99:0:0
2	**Pd-PEPPSI-IPent**		rt	1	44	96:2:2
3	**$Pd_2(dba)_3$**	L3, XPhos	40°C	0.2	Full	95:0:5
4	**Pd-PEPPSI-IPent**		40°C	0.2	34	91:6:3

[a]Conditions: (3-methylbut-2-en-2-yl)lithium (0.40 mmol, 0.6 M in THF) was added to a solution of 4-methoxy-chlorobenzene (0.3 mmol) in toluene (1 mL). [b]**2a:3:4** ratios determined by GC analysis. dba = dibenzylideneacetone.

Scheme S1. Conversion for the reactions between 1- and 2-chloronaphthalene with (3-methylbut-2-en-2-yl)lithium and (*Z*)-propenyllithium[a]

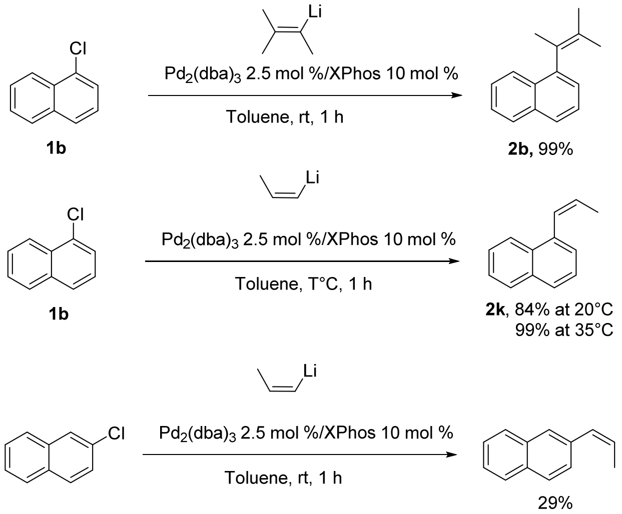

[a]Conditions: RLi (1.5 equiv) was added to a solution of chloronaphthalene (0.3 mmol) in toluene (1 mL), flow rate = 1.0 mL/h. Conversion determined by GC analysis. dba = dibenzylideneacetone.

Table S3. Attempts for the reaction between vinyllithium and 4-methoxy-bromobenzene[a]

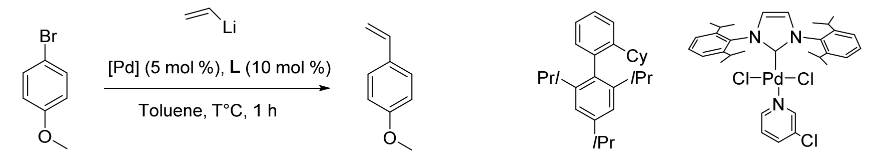

2a **XPhos** **Pd-PEPPSI-IPr**

Entry	[Pd]	Ligand	Conversion%
**1**	$Pd_2(dba)_3$, **L1**, XPhos		5
**2**	PEPPSI-*I*Pr		5
**3**[b]	PEPPSI-*I*Pr		1
**4**[c]	PEPPSI-*I*Pr		1

[a]Conditions: Aryl bromide (0.3 mmol), vinyllithium (0.45 mmol, diluted with THF to reach 0.30 M concentration and added at 1 mL/h flow rate). Toluene (1 mL). Conversion determined by GC analysis [b]Reaction performed at 40 °C. [c]TMEDA (1.2 equiv) was added and the reaction was performed at 40 °C. [d]Synthesis of vinyllithium: In a dry Schlenk flask vinylbromide (5 mmol) was dissolved in dry THF (5 mL) and the solution was cooled down to -78 ºC. *t*BuLi (10.5 mmol, 6.2 mL) was added slowly and the solution was stirred for 20 min. The solution was then allowed to reach room temperature.

Unfortunately, the use of simple vinyl lithium led to less than 5% conversion in the reaction with 4-methoxy-bromobenzene under the optimal reaction conditions. The use of different catalysts, additives or higher temperatures did not improve this result.

Scheme S2. Pd-catalysed cross-coupling of (*Z*)-propenyllithium with 2-bromo-1,3,5-tri-*tert*-butylbenzene[a]

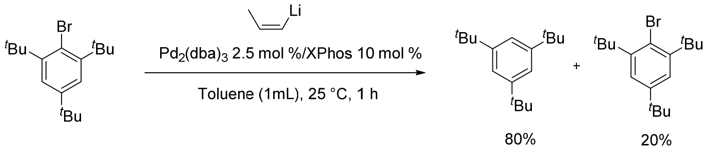

[a]Ratios determined by GC analysis.

Scheme S3. Pd-catalysed cross-coupling of allenyllithium reagents with aryl and alkenyl halides: limitations[a]

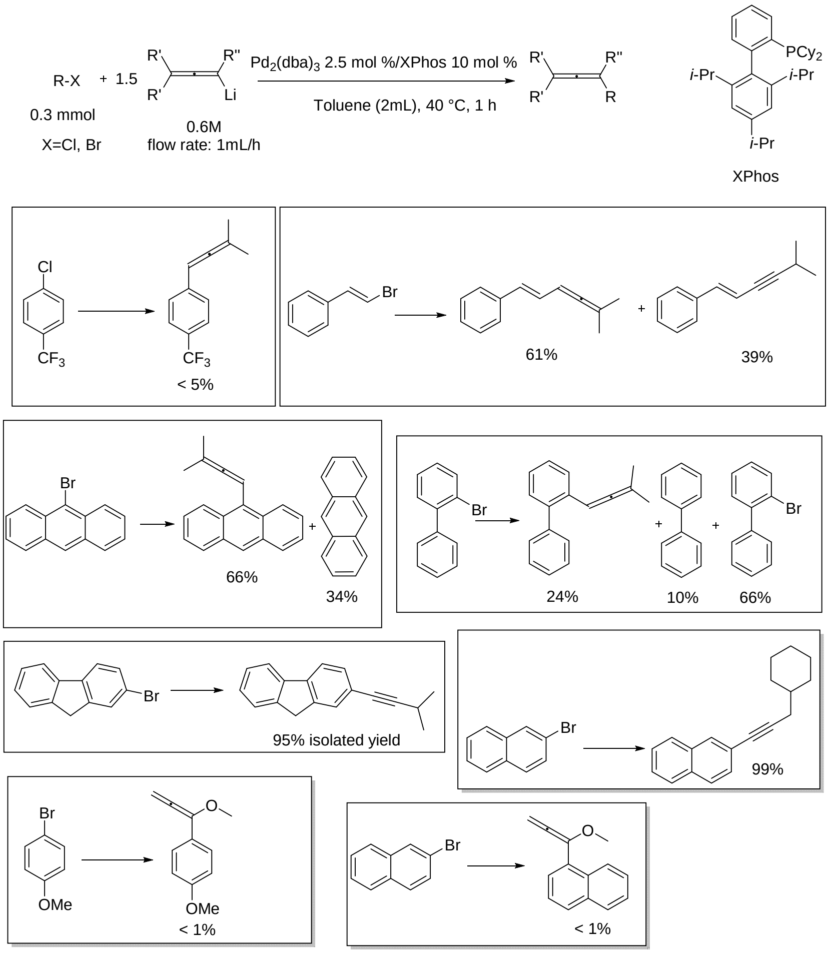

[a]Ratios determined by GC analysis.

General procedure A for the cross-coupling of alkenyllithium reagents.

In a dry Schlenk flask $Pd_2(dba)_3$ (2.5 mol%, 0.0075 mmol, 6.87 mg) and XPhos (10 mol%, 0.03 mmol, 14.3 mg) were dissolved in toluene (2 mL) and the solution was stirred under nitrogen atmosphere at room temperature for 5 min. The substrate (0.3 mmol) was added and the solution stirred at the indicated temperature. The corresponding lithium reagent solution (1.3 equiv, 0.6 or 0.68 M, see below) was slowly added over 1h by the use of a syringe pump. After the addition was completed a saturated solution of aqueous NH_4Cl was added and the mixture was extracted with Et_2O, AcOEt or DCM (3 x 5 mL). The organic phases were combined and dried with anhydrous Na_2SO_4. Evaporation of the solvent under reduced pressure afforded the crude product that was then purified by column chromatography.

General procedure B for the cross-coupling using (1-ethoxyvinyl)lithium (6 mmol scale).

In a dry Schlenk flask $Pd_2(dba)_3$ (1.25 mol%, 0.075 mmol, 69 mg) and XPhos (5 mol%, 0.3 mmol, 143 mg) were dissolved in toluene (7 mL) and the solution was stirred under nitrogen atmosphere at room temperature for 5 min. The substrate (6 mmol) in toluene (8 mL) was added and the temperature raised to 40°C. (1-Ethoxyvinyl)lithium solution in THF (1.5 equiv, 0.6 M, 15 mL) was slowly added over 2.5h by the use of a syringe pump. After the addition was completed, the full conversion into the corresponding ethoxyvinyl ether derivate was confirmed by GC/MS. 2 M aqueous HCl (10 mL) was then added and the mixture was stirred for 10 min at room temperature. The aqueous phase was extracted with EtOAc (3 x 15 mL) and the combined organic phases were washed with brine (1 x 20 mL) and dried with anhydrous Na_2SO_4. Evaporation of the solvent under reduced pressure afforded the crude product that was then purified by column chromatography.

Preparation of alkenyllithium reagents:

Vinyl iodides ((*E*)-*tert*-butyl((3-iodoallyl)oxy)dimethylsilane and (*E*)-*tert*-butyl((4-iodobut-3-en-1-yl)oxy)dimethylsilane) were prepared according to previously reported procedures.[1]

Method 1: (3-methylbut-2-en-2-yl)lithium, (cyclohexylidenemethyl)lithium, (*E*)-(3-((*tert*-butyldimethylsilyl)oxy)prop-1-en-1-yl)lithium and (*E*)-(4-((*tert*-butyldimethylsilyl)oxy)but-1-en-1-yl)lithium.

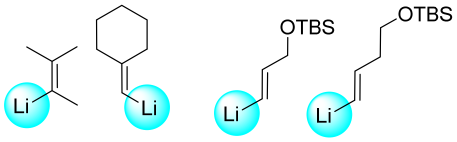

In a dry Schlenk flask the corresponding vinyl halide (2.5 mmol) was dissolved in dry THF (1.25 mL) and the solution was cooled down to -78 ºC. *t*BuLi (2.1 equiv, 3 mL)

[1] Z. Huang and E. Negishi, *Org. Lett.* 2006, **8**, 3675.

was added slowly and the solution was stirred for 20 min. Then the solution was allowed to reach room temperature and stirred for another 20 min.

Method 2: (*Z*)-prop-1-en-1-yllithium, (*E*)-but-2-en-2-yllithium and prop-1-en-2-yllithium [2]

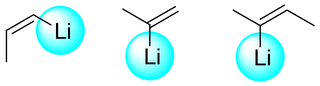

The corresponding bromide (7.5 mmol) dissolved in diethyl ether (1 mL) was added dropwise at -50 °C to a suspension of lithium shot (0.3 g) in diethyl ether (4 to 10 mL). After stirring for 0.5 h at -40 °C, the reaction mixture was slowly allowed to warm to room temperature and stirred for another 15 min. The corresponding lithium reagent solution was then diluted with THF to reach a concentration of 0.68 M.

Method 3: (3-methylbuta-1,2-dien-1-yl)lithium[3] and (3,4-dihydro-2*H*-pyran-6-yl)lithium [4]

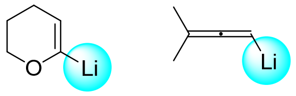

In a dry Schlenk flask 3-methyl-1,2-butadiene or 3,4-dihydro-2*H*-pyran (2.5 mmol) was dissolved in dry THF (2.7 mL) and the solution was cooled down to -78 ºC. *t*BuLi (1 equiv, 2.5 mmol, 1.47 mL) was added dropwise and the solution was stirred for 0.5 h. Then the solution was allowed to reach room temperature.

Method 4: (1-ethoxyvinyl)lithium (6 mmol scale)[4]

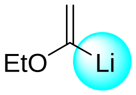

In a dry Schlenk flask 1-ethoxyethylene (20 mmol, 1442 mg, 1915 µL) was dissolved in dry THF (21 mL) and the solution was cooled down to -78 ºC. ^{t}BuLi (1 equiv, 20 mmol, 11.8 mL) was added dropwise and the solution was stirred for 0.5 h. The mixture was allowed to warm to room temperature and stirred for 15 min whereby a pale yellow solution was obtained.

[2] M. Noack and R. Göttlich, *Eur. J. Org. Chem.* 2002, 3171.
[3] W. De Graaf, J. Boersma, G. van Koten and C. J. Elsevier, *J. Organomet. Chem.* 1989, **378**, 115.
[4] S. E. Denmark, and L. Neuville, *Org. Lett.* 2000, **2**, 3221.

Data of Compounds 2a-2u, 6a-6k

Physical data for known compounds were identical in all respects to those previously reported (references are given).

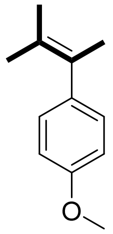

2-(4-Methoxyphenyl)-3-methylbut-2-ene (2a).[5]

(2a, X=Br) Synthesized using the general procedure A with 1-bromo-4-methoxybenzene (0.3 mmol, 56 mg) and 0.75 mL of (3-methylbut-2-en-2-yl)lithium (0.6M). Reaction carried out at room temperature. Colorless oil obtained after column chromatography (SiO_2, *n*-pentane/ Et_2O 100:1), 48 mg, 91% yield. ^1H NMR (400 MHz, $CDCl_3$) δ 7.07 (d, J = 8.6 Hz, 2H), 6.86 (d, J = 8.6 Hz, 2H), 3.82 (s, 3H), 1.96 (s, 3H), 1.81 (s, 3H), 1.62 (s, 3H) ppm. ^{13}C NMR (100 MHz, $CDCl_3$) δ 157.6, 137.7, 129.4, 126.9, 113.3, 55.2, 22.1, 20.9, 20.6 ppm. EI-MS m/z: 176 (100%), 161, 145.

(2a, X=Cl) Synthesized using the general procedure A with 1-chloro-4-methoxybenzene (0.3 mmol, 43 mg) and 0.75 mL of (3-methylbut-2-en-2-yl)lithium (0.6M). Reaction carried out at 40°C in 1 mL of toluene. Colorless oil obtained after column chromatography (SiO_2, *n*-pentane/ Et_2O 100:1), 43 mg, 81% yield.

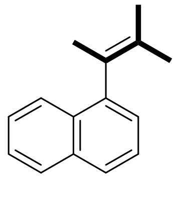

1-(3-methylbut-2-en-2-yl)naphthalene (2b).

Synthesized using the general procedure A with 1-chloronaphthalene (0.3 mmol, 49 mg) and 0.75 mL of (3-methylbut-2-en-2-yl)lithium (0.6M). Reaction carried out at room temperature in 2 mL of toluene. Colorless oil obtained after column chromatography (SiO_2, *n*-pentane/ Et_2O 100:1), 52 mg, 88% yield. ^1H NMR (400 MHz, $CDCl_3$) δ 7.82 – 7.76 (m, 2H), 7.68 (d, J = 8.2 Hz, 1H), 7.43 – 7.38 (m, 2H), 7.15 (d, J = 6.9 Hz, 1H), 1.99 (s, 3H), 1.90 (s, 3H), 1.36 (s, 3H) ppm. ^{13}C NMR (100 MHz, $CDCl_3$) δ 143.5, 133.8, 131.2, 128.8, 128.3, 128.1, 126.2, 125.8, 125.7, 125.6, 125.5, 125.3, 22.1, 21.1, 20.1 ppm. EI-MS m/z: 196, 181 (100%), 165, 153.

[5] F. Berthiol, H. Doucet and M. Santelli, *Eur. J. Org. Chem.* 2003, 1091.

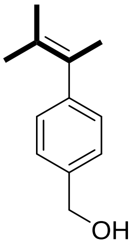

(4-(3-methylbut-2-en-2-yl)phenyl)methanol (2c).

In a dry Schlenk flask (4-bromophenyl)methanol (0.3 mmol, 56 mg) was dissolved in toluene (1.5 mL) and (3-methylbut-2-en-2-yl)lithium (0.5 mL, 0.6M) was added over 5 min. In a separate dry Schlenk flask $Pd_2(dba)_3$ (2.5 mol%, 0.0075 mmol, 6.87 mg) and XPhos (10 mol%, 0.03 mmol, 14.3 mg) were dissolved in toluene (0.5 mL), the solution was stirred under nitrogen atmosphere at room temperature for 5 min and added to the former solution. (3-Methylbut-2-en-2-yl)lithium (0.75 mL, 0.6M) was then slowly added over 1h by the use of a syringe pump. After the addition was completed the reaction mixture was worked up as described in general procedure A. Pale yellow oil obtained after column chromatography (SiO_2, *n*-pentane/AcOEt 7:3), 44 mg, 83% yield. ^{1}H NMR (400 MHz, $CDCl_3$) δ 7.31 (d, *J* = 7.9 Hz, 2H), 7.13 (d, *J* = 8.1 Hz, 2H), 4.65 (s, 2H), 1.96 (s, 3H), 1.82 (s, 3H), 1.60 (s, 3H) ppm. ^{13}C NMR (100 MHz, $CDCl_3$) δ 144.8, 138.2, 129.6, 128.6, 127.4, 126.8, 65.2, 22.1, 20.8, 20.6 ppm. EI-MS m/z: 176 (100%), 161, 145.

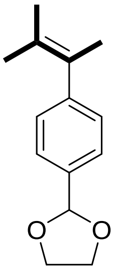

2-(4-(3-methylbut-2-en-2-yl)phenyl)-1,3-dioxolane (2d).

Synthesized using the general procedure A with 2-(4-bromophenyl)-1,3-dioxolane (0.3 mmol, 69 mg) and 0.55 mL of (3-methylbut-2-en-2-yl)lithium (0.6M). Reaction carried out at room temperature. Pale oil obtained after column chromatography (SiO_2, *n*-pentane/ AcOEt 7:1), 57 mg, 87% yield. ^{1}H NMR (400 MHz, $CDCl_3$) δ 7.43 (d, *J* = 7.9 Hz, 2H), 7.15 (d, *J* = 8.2 Hz, 2H), 5.80 (s, 1H), 4.17 – 4.11 (m, 2H), 4.08 – 4.02 (m, 2H), 1.95 (s, 3H), 1.81 (s, 3H), 1.59 (s, 3H) ppm. ^{13}C NMR (100 MHz, $CDCl_3$) δ 146.4, 135.1, 129.6, 128.5, 127.6, 126.1, 103.8, 65.3, 22.0, 20.7, 20.5 ppm. EI-MS m/z: 217 (100%), 203, 173, 146, 131.

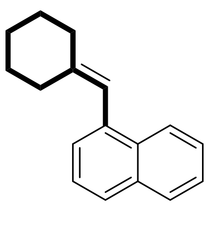

1-(cyclohexylidenemethyl)naphthalene (2e).[6]

Synthesized using the general procedure A with 1-chloronaphthalene (0.3 mmol, 49 mg) and 0.60 mL of (cyclohexylidenemethyl)lithium (0.6M). Reaction carried out at room temperature. White solid obtained after column chromatography (SiO_2, *n*-pentane/ Et_2O 99:1), 60 mg, 90% yield. ^1H NMR (400 MHz, $CDCl_3$) δ 8.10 – 8.05 (m, 1H), 7.89 – 7.86 (m, 1H), 7.77 (d, *J* = 8.2 Hz, 1H), 7.53 – 7.44 (m, 3H), 7.32 (d, *J* = 7.1 Hz, 1H), 6.64 (s, 1H), 2.45 (d, *J* = 6.1 Hz, 2H), 2.23 (d, *J* = 6.1 Hz, 2H), 1.77 (m, 2H), 1.66 (m, 2H), 1.55 (m, 2H) ppm. ^{13}C NMR (100 MHz, $CDCl_3$) δ 144.8, 135.7, 133.6, 132.4, 128.3, 126.7, 126.6, 125.6, 125.5, 125.4, 125.3, 119.7, 37.4, 30.1, 28.9, 28.1, 26.8 ppm. EI-MS m/z: 222, 179, 165 (100%), 153, 141, 128.

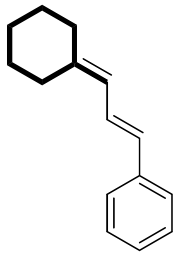

(*E*)-(3-Cyclohexylideneprop-1-en-1-yl)benzene (2f).[7]

Synthesized using the general procedure A with (2-bromovinyl)benzene (4:1 *E/Z* mixture, 0.3 mmol, 55 mg) and 0.60 mL of (cyclohexylidenemethyl)lithium (0.6M). Reaction carried out at room temperature. Colorless oil obtained after column chromatography (SiO_2, *n*-pentane/ Et_2O 99:1), 41 mg, 69% yield. ^1H NMR (400 MHz, $CDCl_3$) δ 7.42 – 7.38 (m, 2H), 7.33 – 7.27 (m, 2H), 7.22 – 7.16 (m, 1H), 7.07 (dd, *J* = 15.5, *J* = 11.1 Hz, 1H), 6.46 (d, *J* = 15.5 Hz, 1H), 5.97 (d, *J* = 11.1 Hz, 1H), 2.40 (s, 2H), 2.22 (s, 2H), 1.61 (s, 6H) ppm. ^{13}C NMR (100 MHz, $CDCl_3$) δ 144.9, 138.1, 129.8, 128.5, 126.8, 126.0, 125.0, 122.3, 37.5, 29.5, 28.6, 27.9, 26.8 ppm. EI-MS m/z: 198 (100%), 183, 169, 155, 141, 129, 115.

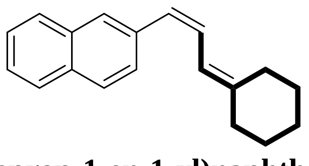

(*Z*)-2-(3-cyclohexylideneprop-1-en-1-yl)naphthalene (2g)

Synthesized using the general procedure A with (*Z*)-2-(2-bromovinyl)naphthalene (0.3 mmol, 70 mg) and 0.60 mL of (cyclohexylidenemethyl)lithium (0.6M). Reaction carried out at room temperature. White solid obtained after column chromatography (SiO_2, *n*-pentane/ Et_2O 99:1), 48 mg, 64% yield. ^1H NMR (400 MHz, $CDCl_3$) δ 7.87 – 7.75 (m, 4H), 7.52 – 7.40 (m, 3H), 6.59 (t, *J* = 11.4 Hz, 1H), 6.48 (d, *J* = 11.6 Hz, 1H), 6.42 (d, *J* = 11.6 Hz, 1H), 2.41 (s, 2H), 2.20 (s, 2H), 1.61 (s, 6H) ppm. ^{13}C NMR (100 MHz, $CDCl_3$) δ 146.9, 135.6, 133.4, 132.2, 127.9, 127.7, 127.6, 127.5, 127.4, 127.3,

---

[6] T. Satoh, N. Hanaki, N. Yamada and T. Asano, *Tetrahedron* **2000**, *56*, 6223.

[7] Y. Horikawa, M. Watanabe, T. Fujiwara and T. Takeda, *J. Am. Chem. Soc.* 1997, **119**, 1127.

126.1, 126.0, 125.6, 118.2, 37.7, 29.3, 28.6, 27.8, 26.8 ppm. EI-MS m/z: 248 (100%), 233, 219, 205, 179, 165, 141.

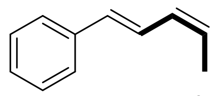

((1*E*,3*Z*)-penta-1,3-dien-1-yl)benzene (2h)[8]

Synthesized using the general procedure A with (2-bromovinyl)benzene (4:1 *E/Z* mixture, 0.3 mmol, 55 mg) and 0.55 mL of (*Z*)-prop-1-en-1-yllithium (0.68M). Reaction carried out at room temperature. Colorless oil obtained after column chromatography (SiO_2, *n*-pentane/ Et_2O 99:1), 34 mg, 79% yield. ^{1}H NMR (400 MHz, $CDCl_3$) δ 7.45 – 7.40 (m, 2H), 7.36 – 7.29 (m, 2H), 7.26 – 7.20 (m, 1H), 7.10 (dd, *J* = 15.7, *J* = 11.2 Hz, 1H), 6.54 (d, *J* = 15.7 Hz, 1H), 6.19 (t, *J* = 11.0 Hz, 1H), 5.61 (m, 1H) 1.87 (d, *J* = 7.2 Hz, 3H) ppm. ^{13}C NMR (100 MHz, $CDCl_3$) δ 137.7, 131.8, 129.6, 128.6, 127.3, 127.2, 126.3, 124.2, 13.6 ppm. EI-MS m/z:144, 129 (100%), 115.

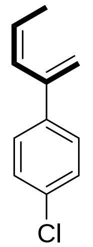

(*Z*)-1-chloro-4-(penta-1,3-dien-2-yl)benzene (2i)

Synthesized using the general procedure A with 1-(1-bromovinyl)-4-chlorobenzene (0.3 mmol, 65 mg) and 0.50 mL of (*Z*)-prop-1-en-1-yllithium (0.68M). Reaction carried out at room temperature. Colorless oil obtained after column chromatography (SiO_2, *n*-pentane/ Et_2O 99:1), 39 mg, 73% yield. ^{1}H NMR (400 MHz, $CDCl_3$) δ 7.31 (m, 4H), 6.12 (d, *J* = 11.7 Hz, 1H), 5.82 (m, 1H), 5.54 (s, 1H), 5.16 (s, 1H), 1.68 (d, *J* = 6.9 Hz, 3H) ppm. ^{13}C NMR (100 MHz, $CDCl_3$) δ 143.2, 139.4, 133.3, 129.3, 128.9, 128.3, 127.9, 115.4, 14.8 ppm. EI-MS m/z: 178, 163, 143, 128 (100%), 115.

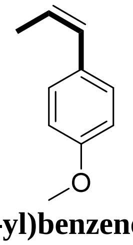

(*Z*)-1-methoxy-4-(prop-1-en-1-yl)benzene (2j).[9]

Synthesized using the general procedure A with 1-bromo-4-methoxybenzene (0.3 mmol, 56.1 mg) and 0.60 mL of (Z)-prop-1-en-1-yllithium (0.68M). Reaction carried out at room temperature. Colorless oil obtained after column chromatography (SiO_2, *n*-pentane/

[8] A. L. Watkins and C. R. Landis, *Org. Lett.* 2011, **13**, 164.
[9] G. Vassilikogiannakis, M. Hatzimarinaki and M. Orfanopoulos, *J. Org. Chem.* 2000, **65**, 8180.

Et_2O 99:2), 37 mg, 83% yield. ^{1}H NMR (400 MHz, $CDCl_3$) δ 7.26 (d, *J* = 8.6 Hz, 2H), 6.89 (d, *J* = 8.6 Hz, 2H), 6.38 (dd, *J* = 11.4, *J* = 1.6 Hz, 1H), 5.71 (m, 1H), 3.82 (s, 3H), 1.90 (dd, *J* = 7.2, *J* = 1.6 Hz, 3H) ppm. ^{13}C NMR (100 MHz, $CDCl_3$) δ 158.1, 130.3, 130.0, 129.3, 125.1, 113.5, 55.2, 14.6 ppm. EI-MS m/z: 148 (100%), 133, 117, 105.

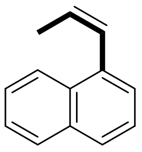

(*Z*)-1-(prop-1-en-1-yl)naphthalene (2k)

Synthesized using the general procedure A with 1-chloronaphthalene (0.3 mmol, 49 mg) and 0.66 mL of (Z)-prop-1-en-1-yllithium (0.68M). Reaction carried out at 35°C. Colorless oil obtained after column chromatography (SiO_2, *n*-pentane/ Et_2O 99:1), 47 mg, 93% yield. ^{1}H NMR (400 MHz, $CDCl_3$) δ 8.05 – 8.00 (m, 1H), 7.91 – 7.85 (m, 1H), 7.79 (d, *J* = 8.1 Hz, 1H), 7.55 – 7.47 (m, 3H), 7.39 (d, *J* = 7.1 Hz, 1H), 6.94 (d, *J* = 11.4 Hz, 1H), 6.12 – 6.04 (m, 1H), 1.79 (dd, *J* = 7.0, *J* = 1.4 Hz, 3H) ppm. ^{13}C NMR (100 MHz, $CDCl_3$) δ 134.6, 133.6, 131.9, 128.6, 128.4, 127.9, 127.1, 126.5, 125.8, 127.7, 125.2, 125.1, 14.6 ppm. EI-MS m/z: 168, 153 (100%).

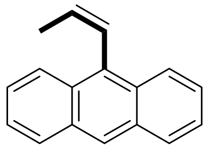

(*Z*)-9-(prop-1-en-1-yl)anthracene (2l)

Synthesized using the general procedure A with 9-bromoanthracene (0.3 mmol, 77 mg) and 0.66 mL of (Z)-prop-1-en-1-yllithium (0.68M). Reaction carried out at room temperature. Pale yellow waxy solid obtained after column chromatography (SiO_2, *n*-pentane/ Et_2O 99:1), 59 mg, 90% yield. ^{1}H NMR (400 MHz, $CDCl_3$) δ 8.44 (s, 1H), 8.25 – 8.19 (m, 2H), 8.08 – 8.02 (m, 2H), 7.54 – 7.48 (m, 4H), 7.09 (d, *J* = 11.3 Hz, 1H), 6.40 (m, 1H), 1.49 (dd, *J* = 6.9, *J* = 1.5 Hz, 3H) ppm. ^{13}C NMR (100 MHz, $CDCl_3$) δ 132.2, 131.4, 130.9, 129.5, 128.7, 128.2, 126.4, 126.1, 125.3, 125.1, 15.0 ppm. EI-MS m/z: 218, 203(100%).

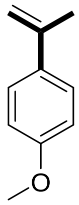

1-methoxy-4-(prop-1-en-2-yl)benzene (2m)[10]

[10] E. Comer, M. Organ and S. J. Hynes, *J. Am. Chem. Soc.* 2004, **126**, 16087.

Synthesized using the general procedure A with 1-bromo-4-methoxybenzene (0.3 mmol, 56 mg) and 0.66 mL of prop-1-en-2-yllithium (0.68M). Reaction carried out at room temperature. Yellow solid obtained after column chromatography (SiO_2, *n*-pentane/ Et_2O 99:2), 34 mg, 76% yield. ^{1}H NMR (400 MHz, $CDCl_3$) δ 7.43 (d, *J* = 8.7 Hz, 2H), 6.88 (d, *J* = 8.7 Hz, 2H), 5.30 (s, 1H), 5.00 (s, 1H), 3.82 (s, 3H), 2.15 (s, 3H) ppm. ^{13}C NMR (100 MHz, $CDCl_3$) δ 159.1, 142.5, 133.8, 126.6, 113.5, 110.6, 55.3, 21.9 ppm EI-MS m/z: 148 (100%), 133.

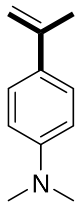

_N,N_-dimethyl-4-(prop-1-en-2-yl)aniline (2n)[11]

Synthesized using the general procedure A with 4-bromo-*N,N*-dimethylaniline (0.3 mmol, 60 mg) and 0.66 mL of prop-1-en-2-yllithium (0.68M). Reaction carried out at room temperature. White solid obtained after column chromatography (SiO_2, *n*-pentane/ Et_2O 99:2), 41 mg, 85% yield. ^{1}H NMR (400 MHz, $CDCl_3$) δ 7.41 (d, *J* = 8.7 Hz, 2H), 6.71 (d, *J* = 8.7 Hz, 2H), 5.27 (s, 1H), 4.92 (s, 1H), 2.97 (s, 6H), 2.14 (s, 3H) ppm. ^{13}C NMR (100 MHz, $CDCl_3$) δ 160.2, 150.0, 142.7, 126.2, 112.2, 108.8, 40.6, 21.8 ppm. EI-MS m/z: 161 (100%), 146, 130, 115.

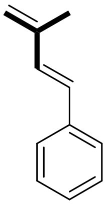

(_E_)-(3-methylbuta-1,3-dien-1-yl)benzene (2o)[12]

Synthesized using the general procedure A with (2-bromovinyl)benzene (4:1 *E/Z* mixture, 0.3 mmol, 55 mg) and 0.66 mL of prop-1-en-2-yllithium (0.68M). Reaction carried out at room temperature. Colorless oil obtained after column chromatography (SiO_2, *n*-pentane/ Et_2O 99:1), 35 mg, 81% yield. ^{1}H NMR (400 MHz, $CDCl_3$) δ 7.45 (d, *J* = 7.9 Hz, 2H), 7.34 (t, *J* = 7.6 Hz, 2H), 7.24 (t, *J* = 6.8 Hz, 1H) 6.90 (d, *J* = 16.2 Hz, 1H), 6.56 (d, *J* = 16.2 Hz, 1H), 5.14 (s, 1H), 5.10 (s, 1H), 2.00 (s, 3H) ppm. ^{13}C NMR (100 MHz, $CDCl_3$) δ 142.1, 137.4, 131.7, 128.7, 128.6, 127.4, 126.5, 117.3, 18.6 ppm. EI-MS m/z: 144 (100%), 129, 115.

[11] E. Peyroux, F. Berthiol, H. Doucet, and M. Santelli, *Eur. J. Org. Chem.* 2004, 1075.
[12] P. Liu, Y. Pan, K. Hu, X. Huang, Y. Liang and H. Wang, *Tetrahedron* 2013, **69**, 7925.

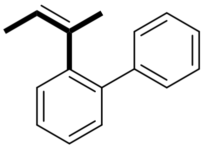

(*Z*)-2-(but-2-en-2-yl)-1,1'-biphenyl (2p)

Synthesized using the general procedure A with 2-bromo-1,1'-biphenyl (0.3 mmol, 70 mg) and 0.75 mL of (*E*)-but-2-en-2-yllithium (0.68M). Reaction carried out at room temperature. Colorless oil obtained after column chromatography (SiO_2, *n*-pentane/ Et_2O 99:1), 57 mg, 91% yield. ^1H NMR (400 MHz, $CDCl_3$) δ 7.51 – 7.31 (m, 9H), 7.23 – 7.19 (m, 1H), 5.45 (q, J = 6.8 Hz, 1H), 1.75 (s, 3H), 1.45 (d, J = 6.6 Hz, 3H) ppm. ^{13}C NMR (100 MHz, $CDCl_3$) δ 141.9, 140.6, 140.5, 137.2, 130.02, 129.5, 128.7, 127.9, 127.1, 126.9, 126.7, 122.8, 25.2, 15.0 ppm. EI-MS m/z: 208, 193 (100%), 178, 152.

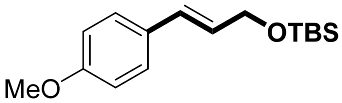

(*E*)-*tert*-butyl((3-(4-methoxyphenyl)allyl)oxy)dimethylsilane (2q)

Synthesized using the general procedure A with 1-bromo-4-methoxybenzene (0.3 mmol, 56 mg) and 0.75 mL of (*E*)-(3-((*tert*-butyldimethylsilyl)oxy)prop-1-en-1-yl)lithium (0.60M). Reaction carried out at room temperature. Yellow solid obtained after column chromatography (SiO_2, *n*-pentane/ AcOEt 99:1), 54 mg, 65% yield. The instability of the product to silica gel necessitated the use of rapid flash chromatography. Spectral data match those previously reported.[13]

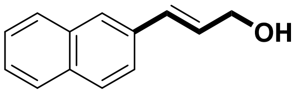

(*E*)-3-(naphthalen-2-yl)prop-2-en-1-ol (2r)

Synthesized using the general procedure A with 2-bromonaphthalene (0.3 mmol, 62 mg) and 0.75 mL of (*E*)-(3-((*tert*-butyldimethylsilyl)oxy)prop-1-en-1-yl)lithium (0.60M). Reaction carried out at room temperature. The reaction crude was worked up as described in general procedure A, dissolved in THF (1mL) and then treated with TBAF (0.5 mL, 0.5 mmol, 1.7 equiv., 1.0 M in THF). After stirring at room temperature for 1 h, the solution was quenched with sat. aq. NH_4Cl, the aqueous layer was extracted with EtOAc (3 x 10 mL), and the combined organic layers were dried with anhydrous Na_2SO_4, filtered and concentrated under reduced pressure. White solid obtained after column chromatography (SiO_2, *n*-pentane/ AcOEt 4:1), 28 mg, 51% yield. Spectral data match those previously reported.[14]

[13] M. Seki, and K, Mori, *Eur. J. Org. Chem.* 1999, 2965.
[14] A. B. Charette, C. Molinaro, and C. Brochu, *J. Am. Chem. Soc.* 2001, **123**, 12168.

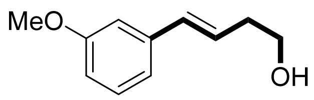

(*E*)-4-(3-methoxyphenyl)but-3-en-1-ol (2s)

Synthesized using the general procedure A with 1-bromo-3-methoxybenzene (0.3 mmol, 56 mg) and 0.75 mL of (*E*)-(4-((*tert*-butyldimethylsilyl)oxy)but-1-en-1-yl)lithium (0.60M). Reaction carried out at room temperature. The reaction crude was worked up as described in general procedure A, dissolved in THF (1mL) and then treated with TBAF (0.5 mL, 0.5 mmol, 1.7 equiv., 1.0 M in THF). After stirring at room temperature for 1 h, the solution was quenched with sat. aq. NH_4Cl, the aqueous layer was extracted with EtOAc (3 x 10 mL), and the combined organic layers were dried with anhydrous Na_2SO_4, filtered and concentrated under reduced pressure. Pale yellow oil obtained after column chromatography (SiO_2, *n*-pentane/ AcOEt 4:1), 36 mg, 67% yield. Spectral data match those previously reported.[15]

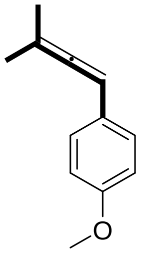

1-methoxy-4-(3-methylbuta-1,2-dien-1-yl)benzene (2t).[16]

Synthesized using the general procedure A with 1-bromo-4-methoxybenzene (0.3 mmol, 56 mg) and 0.75 mL of (3-methylbuta-1,2-dien-1-yl)lithium (0.6M). Reaction carried out at 40°C. Colorless oil obtained after column chromatography (SiO_2, *n*-pentane/ Et_2O 99:2), 42 mg, 80% yield. ^{1}H NMR (400 MHz, $CDCl_3$) δ 7.20 (d, *J* = 8.8 Hz, 2H), 6.84 (d, *J* = 8.8 Hz, 2H), 5.96 (q, *J* = 2.9 Hz, 1H), 3.80 (s, 3H), 1.82 (d, *J* = 2.9 Hz, 6H) ppm. ^{13}C NMR (100 MHz, $CDCl_3$) δ 202.4, 158.4, 128.3, 127.6, 114.0, 99.0, 91.9, 55.3, 20.4 ppm. EI-MS m/z: 174 (100%), 159, 144, 128, 115.

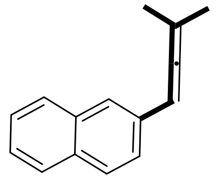

1-(3-methylbuta-1,2-dien-1-yl)naphthalene (2u).

Synthesized using the general procedure A with 2-bromonaphthalene (0.3 mmol, 62 mg) and 0.75 mL of (3-methylbuta-1,2-dien-1-yl)lithium (0.6M). Reaction carried out at 40°C. White solid obtained after column chromatography (SiO_2, *n*-pentane/ Et_2O

[15] X. Zeng, C. Miao, S. Wang, C. Xia and W. Sun, *Chem. Commun.* 2013, **49**, 2418.
[16] M. A. Schade, S. Yamada and P. Knochel, *Chem. Eur. J.* 2011, **17**, 4232.

99:1), 45 mg, 77% yield. ^{1}H NMR (400 MHz, $CDCl_3$) δ 7.83 – 7.76 (m, 3H), 7.65 (s, 1H), 7.52 – 7.40 (m, 3H), 6.21 (q, J = 2.8 Hz, 1H), 1.90 (d, J = 2.8 Hz, 6H) ppm. ^{13}C NMR (100 MHz, $CDCl_3$) δ 203.7, 133.8, 133.6, 132.5, 128.0, 127.7, 127.6, 126.1, 125.3, 125.1, 124.9, 99.4, 92.9, 20.4 ppm. EI-MS m/z: 194, 179 (100%), 165, 152.

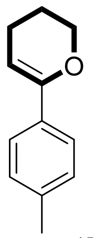

6-(*p*-tolyl)-3,4-dihydro-2*H*-pyran (2v)[17]

Synthesized using the general procedure A with 1-bromo-4-methylbenzene (0.9 mmol, 154 mg) and 2.25 mL of (3,4-dihydro-2*H*-pyran-6-yl)lithium (0.6M). Reaction carried out at 60°C in 3 mL of toluene. The reaction was then quenched by the addition of a few drops of methanol and the solution filtered through a short plug of celite. The instability of the product to silica gel necessitated the use of rapid flash chromatography. Pale yellow oil obtained after column chromatography (SiO_2, *n*-pentane/ AcOEt 50:1 + 1% Et_3N), 111 mg, 71% yield. ^{1}H NMR (400 MHz, $CDCl_3$) δ 6.97 (d, J = 8.2 Hz, 2H), 6.65 (d, J = 8.2 Hz, 2H), 4.81 (t, J = 4.1 Hz, 1H), 3.70 (t, J = 5.1 Hz, 1H), 1.86 (s, 3H), 1.73 (m, 2H), 1.43 (m, 2H) ppm. ^{13}C NMR (100 MHz, $CDCl_3$) δ 151.8, 137.4, 133.5, 128.8, 124.3, 96.5, 66.4, 22.5, 21.2, 20.9 ppm. . EI-MS m/z: 174, 159, 145, 131, 119 (100%).

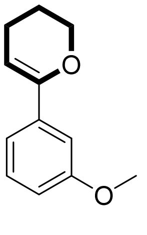

6-(3-methoxyphenyl)-3,4-dihydro-2*H*-pyran (2w)

Synthesized using the general procedure A with 1-bromo-3-methoxybenzene (0.9 mmol, 168 mg) and 2.25 mL of (3,4-dihydro-2*H*-pyran-6-yl)lithium (0.6M). Reaction carried out at 60°C in 3 mL of toluene. The reaction was then quenched by the addition of a few drops of methanol and the solution filtered through a short plug of celite. The instability of the product to silica gel necessitated the use of rapid flash chromatography. Pale yellow oil obtained after column chromatography (SiO_2, *n*-pentane/ AcOEt 50:1 + 1% Et_3N), 138 mg, 81% yield. ^{1}H NMR (400 MHz, $CDCl_3$) δ 7.25 (d, J = 7.9 Hz, 1H), 7.19 – 7.13 (m, 2H), 6.84 (ddd, J = 8.1, 2.6, 0.8 Hz, 1H), 5.37 (t, J = 4.0 Hz, 1H), 4.20 (t, J = 5.1 Hz, 1H), 3.83 (s, 3H), 2.23 (m, 2H), 1.93 (m, 2H) ppm. ^{13}C NMR (100 MHz, $CDCl_3$) δ 159.6, 151.6, 137.8, 129.0, 116.9, 113.6, 109.7, 97.7, 66.5, 55.2, 22.4, 20.9 ppm. EI-MS m/z: 190 (100%), 175, 159, 147, 135.

[17] U. Lehmann, S. Awasthi and T. Minehan, *Org. Lett.* 2003, **5**, 2405.

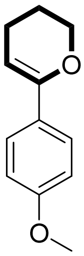

6-(4-methoxyphenyl)-3,4-dihydro-2*H*-pyran (2x)[17]

Synthesized using the general procedure A with 1-bromo-4-methoxybenzene (0.9 mmol, 168 mg) and 2.25 mL of (3,4-dihydro-2*H*-pyran-6-yl)lithium (0.6M). Reaction carried out at 60°C in 3 mL of toluene. The reaction was then quenched by the addition of a few drops of methyl alcohol and the solution filtered through a short plug of celite. The instability of the product to silica gel necessitated the use of rapid flash chromatography. Pale yellow oil obtained after column chromatography (SiO_2, *n*-pentane/ AcOEt 50:1 + 1% Et_3N), 130 mg, 76% yield. ^1H NMR (400 MHz, $CDCl_3$) δ 7.49 (d, *J* = 8.9 Hz, 2H), 6.86 (d, *J* = 8.9 Hz, 2H), 5.22 (t, *J* = 4.0 Hz, 1H), 4.17 (t, *J* = 5.1 Hz, 1H), 3.81 (s, 3H), 2.21 (m, 2H), 1.91 (m, 2H) ppm. ^{13}C NMR (100 MHz, $CDCl_3$) δ 159.3, 151.4, 129.1, 125.6, 113.4, 95.6, 66.5, 55.2, 22.5, 20.8 ppm. EI-MS m/z: 190, 162, 135 (100%).

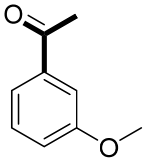

1-(3-methoxyphenyl)ethan-1-one (6a)

Synthesized using the general procedure B with 1-bromo-3-methoxybenzene (6.0 mmol, 1122 mg). Colorless oil obtained after column chromatography (SiO_2, *n*-pentane/ AcOEt 9:1), 734 mg, 81% yield. Spectral data match those previously reported.[18]

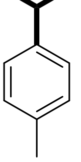

1-(*p*-tolyl)ethanone (6b)

Synthesized using the general procedure B with 1-bromo-4-methylbenzene (4.0 mmol, 684 mg). Colorless oil after column chromatography (SiO_2, *n*-pentane/ AcOEt 9:1), 412 mg, 77% yield. Spectral data match those previously reported.[19]

---

[18] G. Zhang and S. K. Hanson, *Org. Lett.* 2013, **15**, 650.
[19] A. Hamasaki, H. Kuwada and M. Tokunaga, *Tetrahedron Lett.* 2012, **53**, 811.

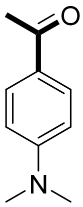

4-*N,N*-Diethylaminoacetophenone (6c)[20]

Synthesized using the general procedure B with 4-bromo-*N,N*-dimethylaniline (6.0 mmol, 1200 mg). pale yellow solid after column chromatography (SiO_2, *n*-pentane/ AcOEt 9:1), 782 mg, 80% yield. ^{1}H NMR (400 MHz, $CDCl_3$) δ 7.86 (d, *J* = 9.1 Hz, 2H), 6.64 (d, *J* = 9.1 Hz), 3.04 (s, 6H), 2.50 (s, 3H) ppm. ^{13}C NMR (100 MHz, $CDCl_3$) δ 196.4, 153.4, 130.5, 110.6, 40.0, 26.0 ppm. EI-MS m/z: 163, 148 (100%).

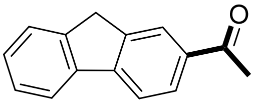

1-(9*H*-fluoren-2-yl)ethanone (6d)

Synthesized using the general procedure B with 2-bromo-9*H*-fluorene (6.0 mmol, 1470 mg). White solid after column chromatography (SiO_2, *n*-pentane/ AcOEt 9:1), 908 mg, 73% yield. Spectral data match those previously reported.[21]

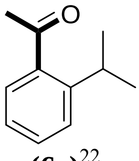

1-(2-isopropylphenyl)-ethanone (6e)[22]

Synthesized using the general procedure B with 1-bromo-2-isopropylbenzene (6.0 mmol, 1195 mg). Colorless oil after column chromatography (SiO_2, *n*-pentane/ AcOEt 9:1), 543 mg, 56% yield. ^{1}H NMR (400 MHz, $CDCl_3$) δ 7.50 – 7.39 (m, 3H), 7.26 – 7.20 (m, 1H), 3.46 (septuplet, *J* = 6.9 Hz, 1H), 2.57 (s, 3H), 1.24 (d, *J* = 6.9 Hz, 6H) ppm. ^{13}C NMR (100 MHz, $CDCl_3$) δ 203.7, 147.7, 138.9, 131.0, 127.6, 126.5, 125.4, 30.7, 29.3, 24.1 ppm. EI-MS m/z: 162, 147 (100%), 129, 115, 103.

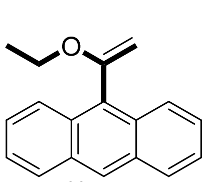

9-(1-ethoxyvinyl)anthracene (5f)[23]

---

[20] C. Herbivo, A. Comel, G. Kirsch and M. M. M. Raposo, *Tetrahedron* 2009**,** **65**, 2079.
[21] S. J. Hwang, H. J. Kim and S. Chang, *Org. Lett.* 2009, **11**, 4588.
[22] G. Cahiez, D. Luart and F. Lecomte, *Org. Lett*. 2004, **6**, 4395.
[23] Z. Rappoport, P. Shulman and M. Thuval. *J. Am. Chem. Soc.* 1978, **100**, 7041.

Synthesized using the general procedure B with 9-bromoanthracene (6.0 mmol, 1543 mg). pale yellow solid after column chromatography (SiO_2, *n*-pentane/ AcOEt 9:1), 1033 mg, 78% yield. ^{1}H NMR (400 MHz, $CDCl_3$) δ 8.49 (s, 1H), 8.31 (d, *J* = 8.6 Hz, 2H), 8.03 (d, *J* = 8.6 Hz, 2H), 7.56 – 7.48 (m, 4H), 4.94 (d, *J* = 2.0 Hz, 1H), 4.48 (d, *J* = 2.0 Hz, 1H), 4.19 (c, *J* = 7.0 Hz, 2H), 1.47 (t, *J* = 7.0 Hz, 3H) ppm. ^{13}C NMR (100 MHz, $CDCl_3$) δ 157.6, 132.6, 131.4, 129.9, 128.4, 127.6, 126.3, 125.8, 125.2, 89.4, 63.6, 14.7 ppm. EI-MS m/z: 248, 219, 202, 191 (100%), 176, 164.

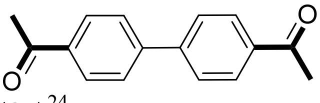

4,4'-Bisacetylbiphenyl (6g)[24]

Synthesized using the general procedure B with 4,4'-dibromo-1,1'-biphenyl (3.0 mmol, 936 mg). White solid after column chromatography (SiO_2, *n*-pentane/ AcOEt 7:1), 543 mg, 76% yield. ^{1}H NMR (400 MHz, $CDCl_3$) δ 8.01 (d, *J* = 8.3 Hz, 4H), 7.68 (d, *J* = 8.3 Hz, 4H), 2.61 (s, 6H) ppm. ^{13}C NMR (100 MHz, $CDCl_3$) δ 197.5, 144.3, 136.5, 129.0, 127.4, 26.7 ppm.

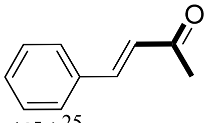

(*E*)-4-phenylbut-3-en-2-one (6h)[25]

Synthesized using the general procedure B with (2-bromovinyl)benzene (4:1 *E/Z* mixture, 6.0 mmol, 770 mg). Orange solid after column chromatography (SiO_2, *n*-pentane/ AcOEt 9:1), 535 mg, 61% yield. ^{1}H NMR (400 MHz, $CDCl_3$) δ 7.55 –7.47 (m, 3H), 7.40 – 7.36 (m, 3H), 6.70 (d, *J* = 16.3 Hz, 1H), 2.37 (s, 3H) ppm. ^{13}C NMR (100 MHz, $CDCl_3$) δ 198.3, 148.4, 134.4, 130.5, 129.0, 128.2, 127.1, 27.5 ppm. EI-MS m/z: 145 (100%), 131 115, 102.

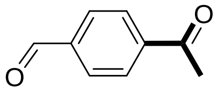

4-acetylbenzaldehyde (6i)[26]

Synthesized using the general procedure B with 2-(4-bromophenyl)-1,3-dioxolane (5.24 mmol, 1200 mg). Yellow solid after column chromatography (SiO_2, *n*-pentane/ AcOEt 9:1), 720 mg, 93% yield. ^{1}H NMR (400 MHz, $CDCl_3$) δ 10.09 (s, 1H), 8.08 (d, *J* = 8.0 Hz, 2H), 7.96 (d, *J* = 8.0 Hz, 2H), 2.64 (s, 3H) ppm. ^{13}C NMR (100 MHz, $CDCl_3$) δ 197.3, 191.6, 141.1, 139.1, 129.8, 128.8, 26.9 ppm. EI-MS m/z: 148, 133 (100%), 105.

---

[24] C. F. Nising, U. K. Schmid, M. Nieger and S. Bräse, *J. Org. Chem.* 2004, **69**, 6830.
[25] M. McConville, O. Saidi, J. Blacker and J. Xiao, *J. Org. Chem.* 2009,**74**, 2692.
[26] S. Liu, N. Berry, N. Thomson, A. Pettman, Z. Hyder, J. Mo and J. Xiao, *J. Org. Chem.* 2006, **71**, 7467.

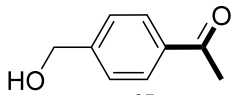

4'-Hydroxymethylacetophenone (6j)[27]

Isopropylmagnesium bromide 2M (4.28 mmol, 2.14 mL) was added in 5 min to a solution of (4-bromophenyl)methanol (4.28 mmol, 800 mg) in toluene (6.7 mL). In a separate dry Schlenk flask $Pd_2(dba)_3$ (1.25 mol%, 50 mg) and XPhos (5 mol%, 102 mg) were dissolved in toluene (4 mL), the solution was stirred under nitrogen atmosphere at room temperature for 5 min and added to the former solution. The temperature was raised to 40°C and (1-ethoxyvinyl)lithium solution in THF (1.5 equiv, 0.6 M, 10.7 mL) was slowly added over 2.5 h by the use of a syringe pump. After the addition was completed the reaction was worked up as described in general procedure B. **6j**, Yellow solid after column chromatography (SiO_2, *n*-pentane/ AcOEt 1:1), 429 mg, 67% yield. ^1H NMR (400 MHz, $CDCl_3$) δ 7.88 (d, J = 8.4 Hz, 2H), 7.40 (d, J = 8.4 Hz, 2H), 4.72 (s, 2H), 2.55 (s, 3H) ppm. ^{13}C NMR (100 MHz, $CDCl_3$) δ 198.3, 146.5, 136.1, 128.6, 126.6, 64.4, 26.6 ppm. EI-MS m/z: 150, 135 (100%).

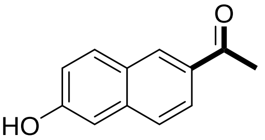

6-Acyl-2-hydroxynaphthalene (6k)

Isopropylmagnesium bromide 2M (6 mmol, 3 mL) was added in 5 min over a suspension of 6-bromonaphthalen-2-ol (6 mmol, 1338 mg) in toluene (10 mL). In a separate dry Schlenk flask $Pd_2(dba)_3$ (1.25 mol%, 69 mg) and XPhos (5 mol%, 143 mg) were dissolved in toluene (5 mL), the solution was stirred under nitrogen atmosphere at room temperature for 5 min and added over the former solution. The temperature was then raised to 40°C and (1-ethoxyvinyl)lithium solution in THF (1.5 equiv, 0.6 M, 15 mL) was slowly added over 2.5h by the use of a syringe pump. After the addition was completed the reaction was worked up as described in general procedure B. **6h**, White solid after column chromatography (SiO_2, *n*-pentane/ AcOEt 1:1), 849 mg, 76% yield. Spectral data match those previously reported.[28]

[27] J. Ruan, X. Li, O. Saidi, J. Xiao. *J. Am. Chem. Soc.* 2008, **130**, 2424.
[28] S. Grunder, R. Huber, S. Wu, C. Schönenberger, M. Calame and M. Mayor *Eur. J. Org. Chem.* **2010**, 833.

^{1}H and ^{13}C NMR spectra

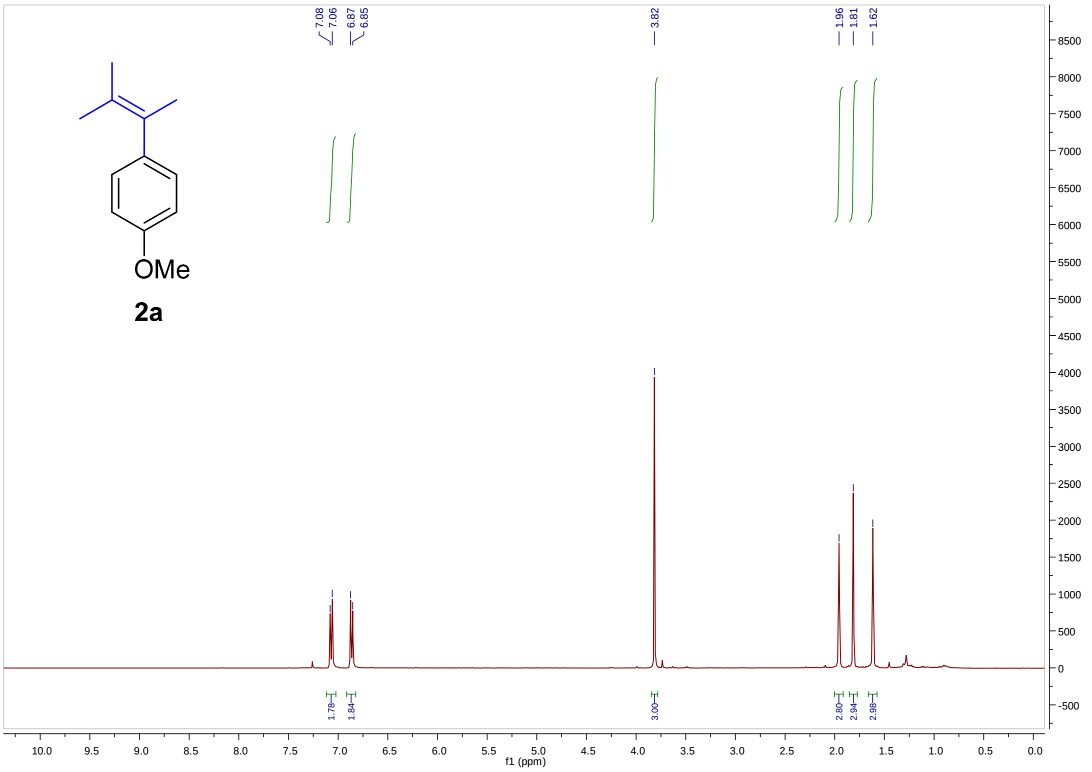

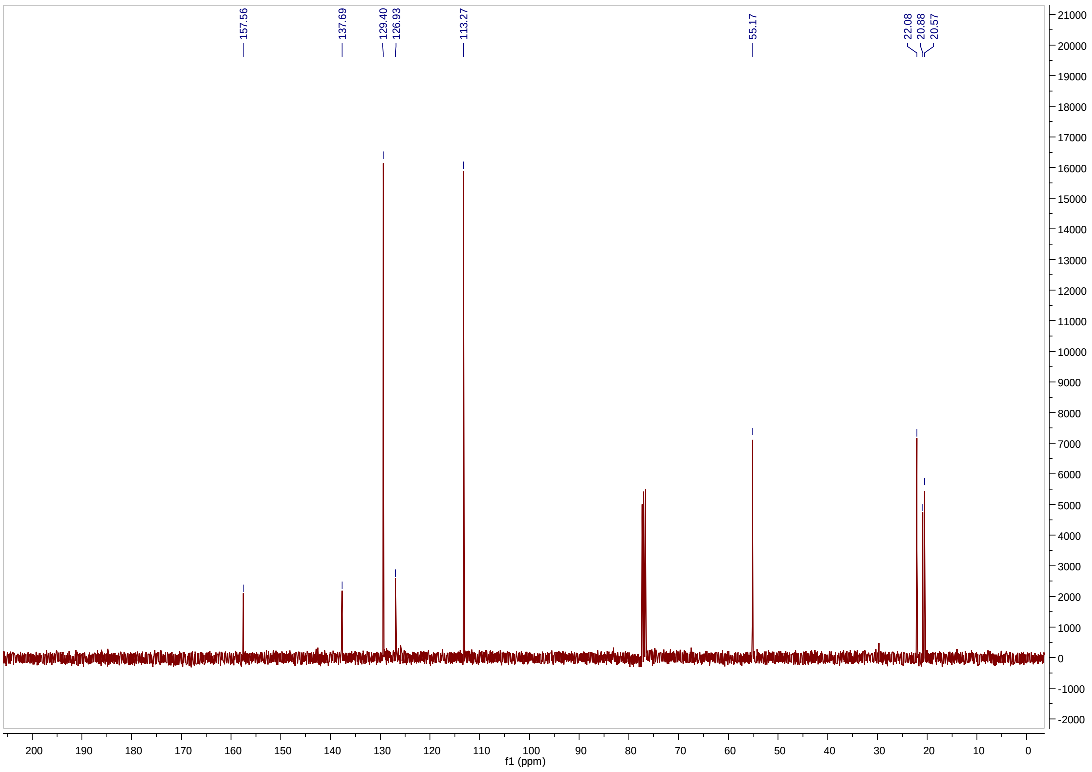

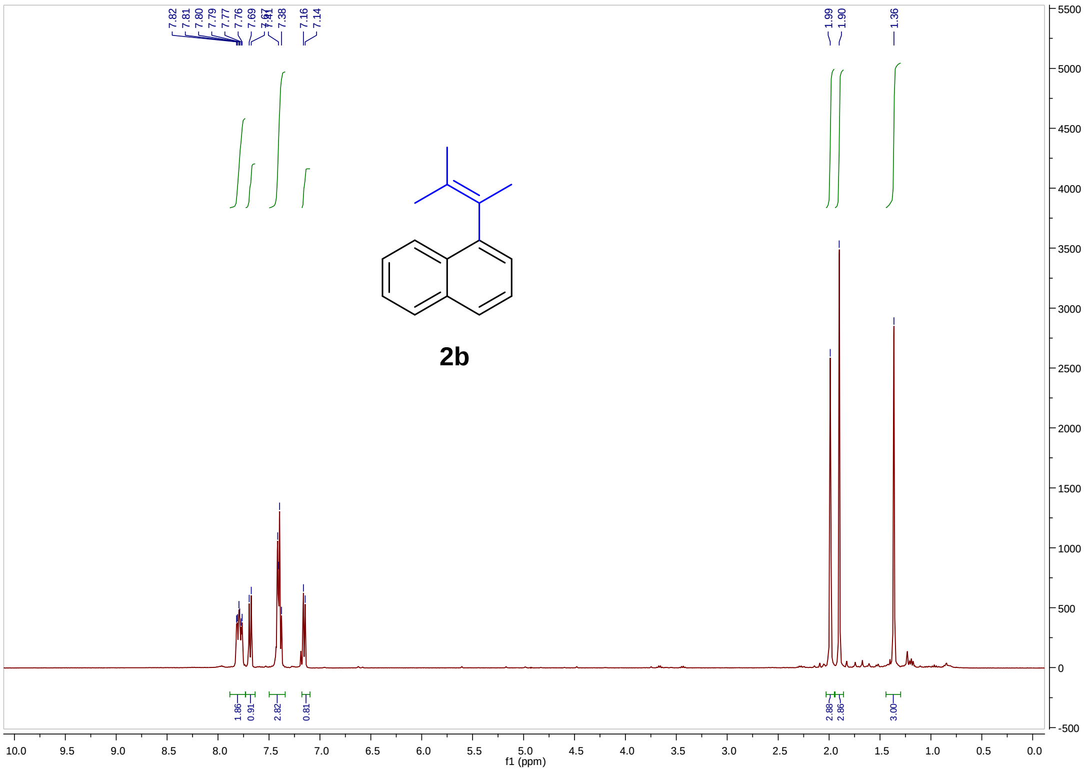
7.82
7.81
7.80
7.79
7.77
7.76
7.69
7.67
7.41
7.38
7.16
7.14
1.99
1.90
1.36
2b
1.86
0.91
2.82
0.81
2.88
2.86
3.00
f1 (ppm)

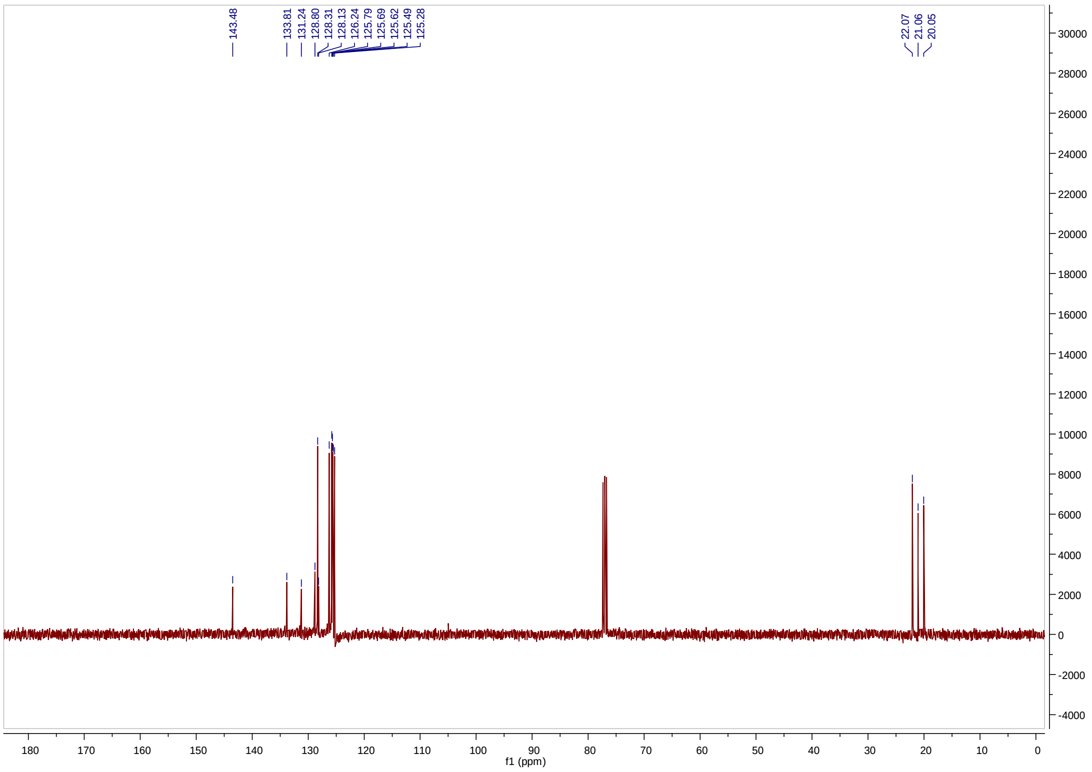
143.48
133.81
131.24
128.80
128.31
128.13
126.24
125.79
125.69
125.62
125.49
125.28
22.07
21.06
20.05
f1 (ppm)

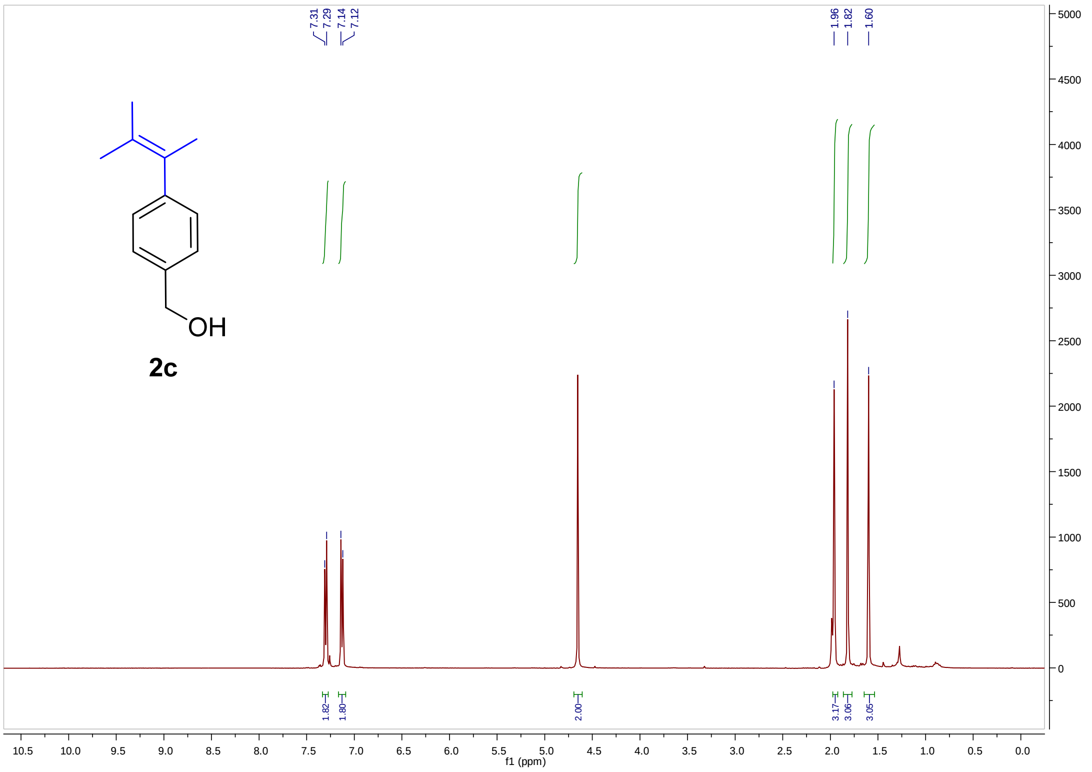
7.31
7.29
7.14
7.12
1.96
1.82
1.60
OH
2c
1.82
1.80
2.00
3.17
3.06
3.05
f1 (ppm)

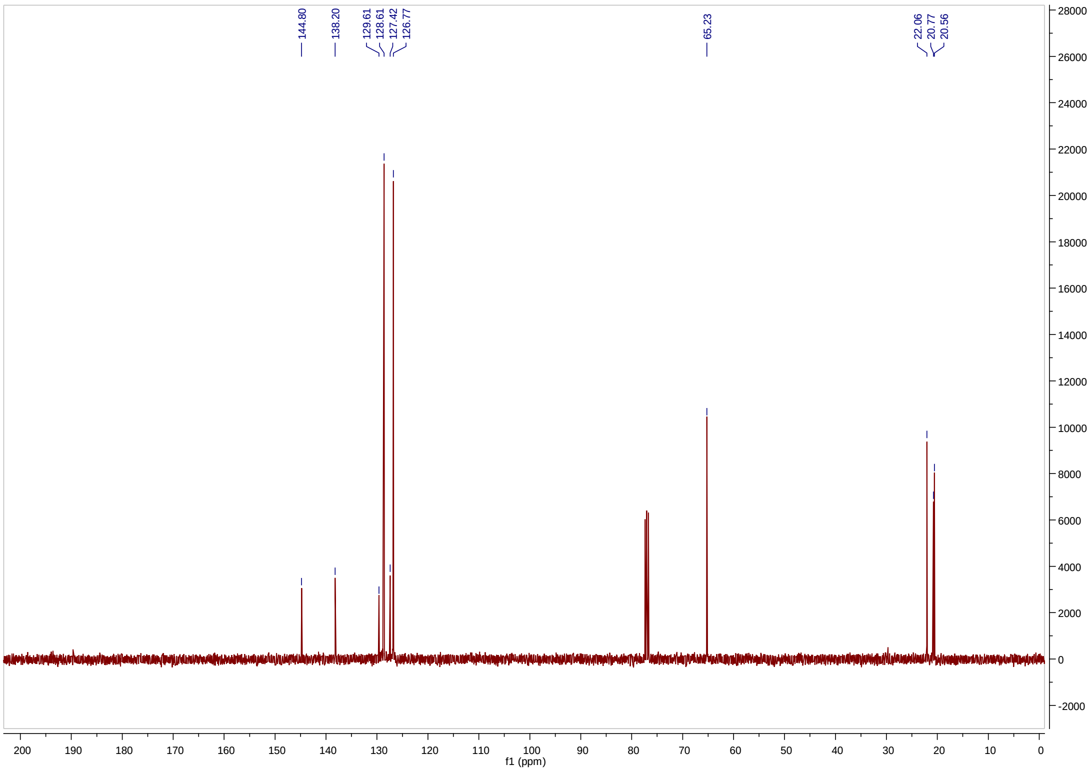
144.80
138.20
129.61
128.61
127.42
126.77
65.23
22.06
20.77
20.56
f1 (ppm)

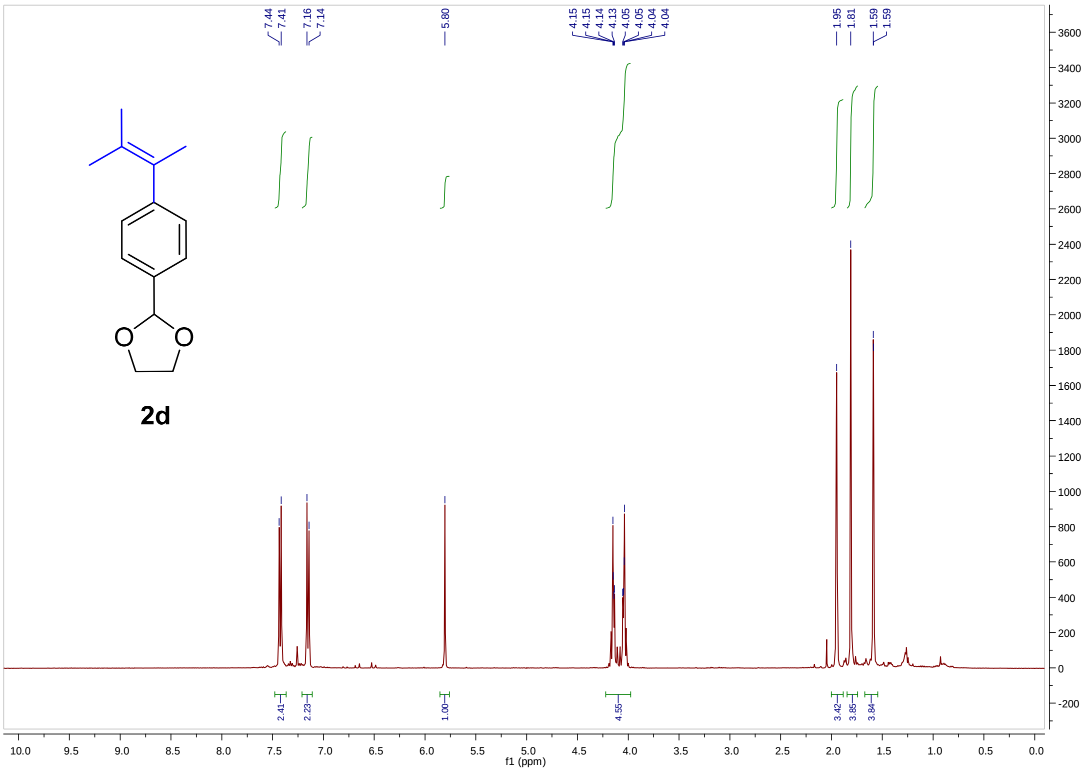
7.44
7.41
7.16
7.14
5.80
4.15
4.15
4.14
4.13
4.05
4.05
4.04
4.04
1.95
1.81
1.59
1.59
O
O
2d
2.41
2.23
1.00
4.55
3.42
3.85
3.84
f1 (ppm)

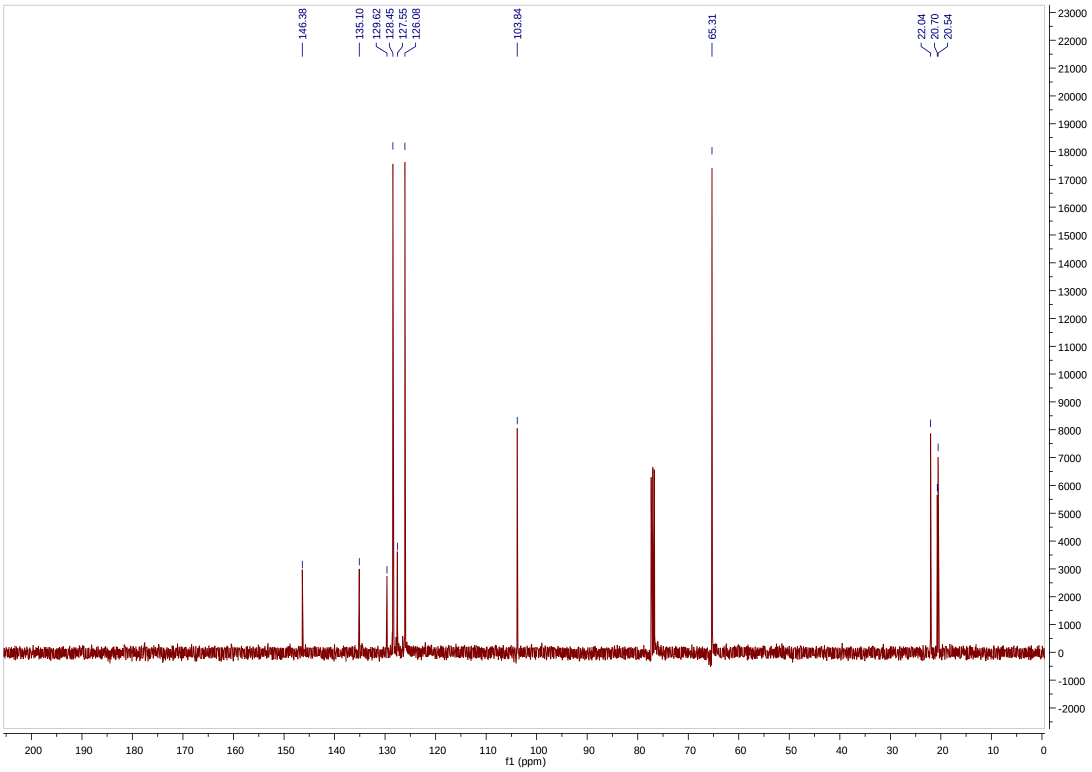
146.38
135.10
129.62
128.45
127.55
126.08
103.84
65.31
22.04
20.70
20.54
f1 (ppm)

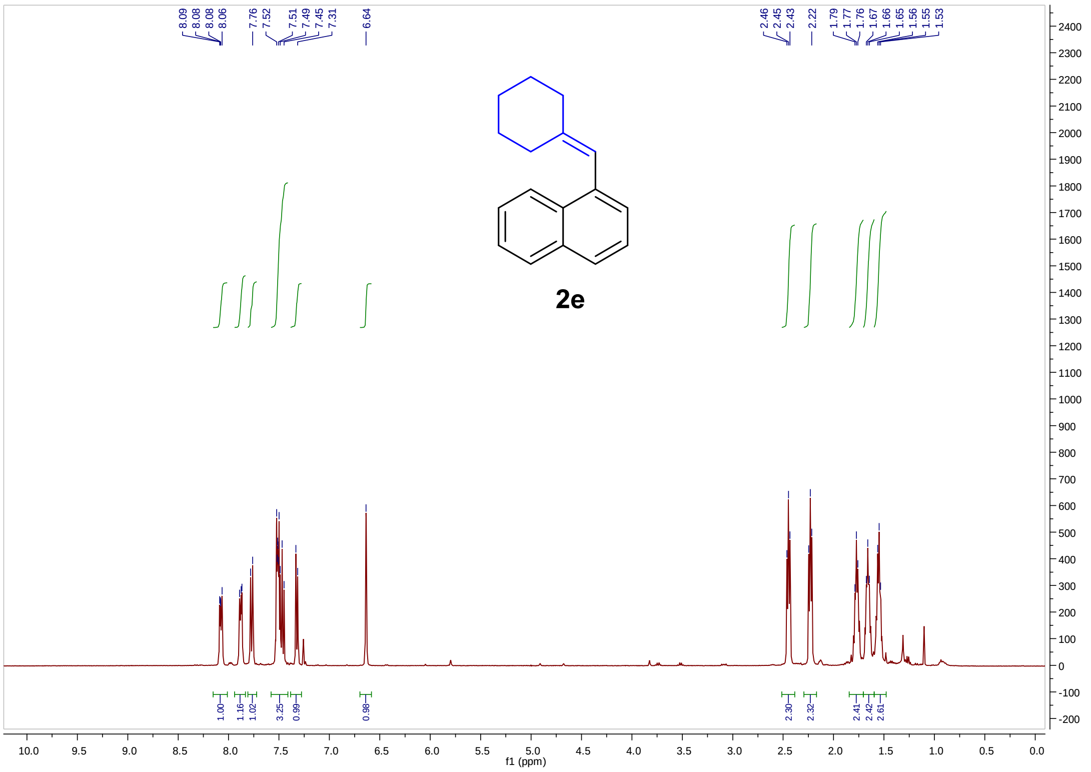
8.09 8.08 8.08 8.06 7.76 7.52 7.51 7.49 7.45 7.31 6.64
2.46 2.45 2.43 2.22 1.79 1.77 1.76 1.67 1.66 1.65 1.56 1.55 1.53
2e
1.00 1.16 1.02 3.25 0.99 0.98 2.30 2.32 2.41 2.42 2.61
f1 (ppm)

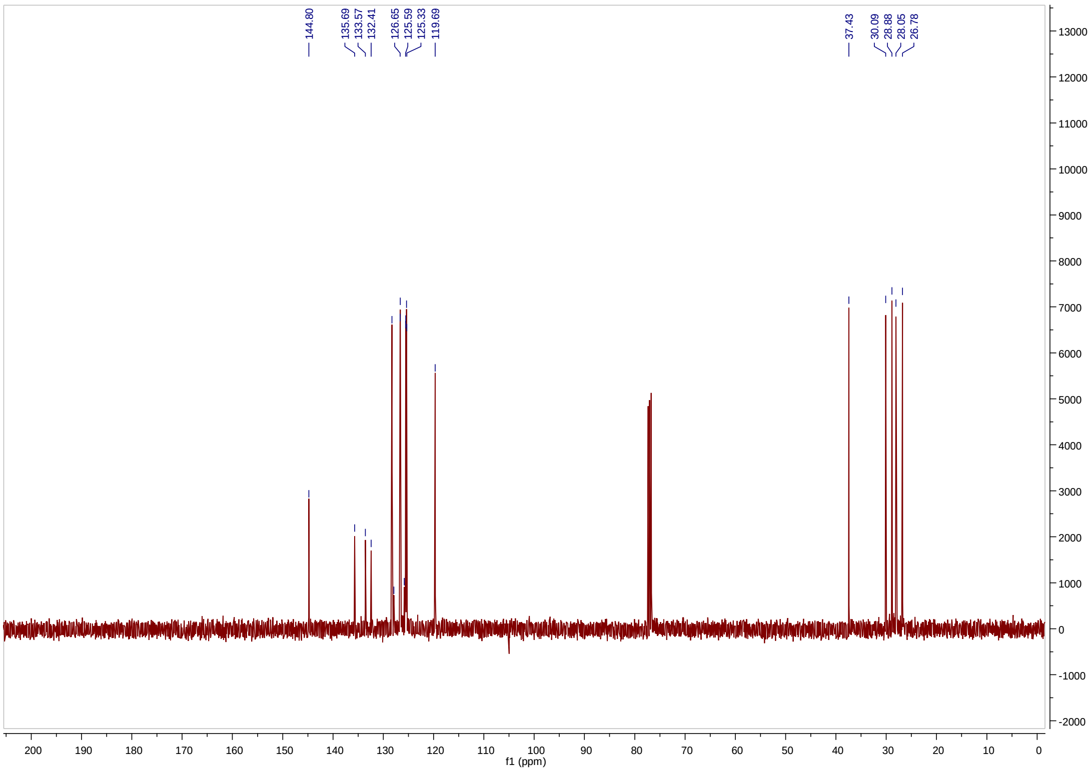
144.80 135.69 133.57 132.41 126.65 125.59 125.33 119.69
37.43 30.09 28.88 28.05 26.78
f1 (ppm)

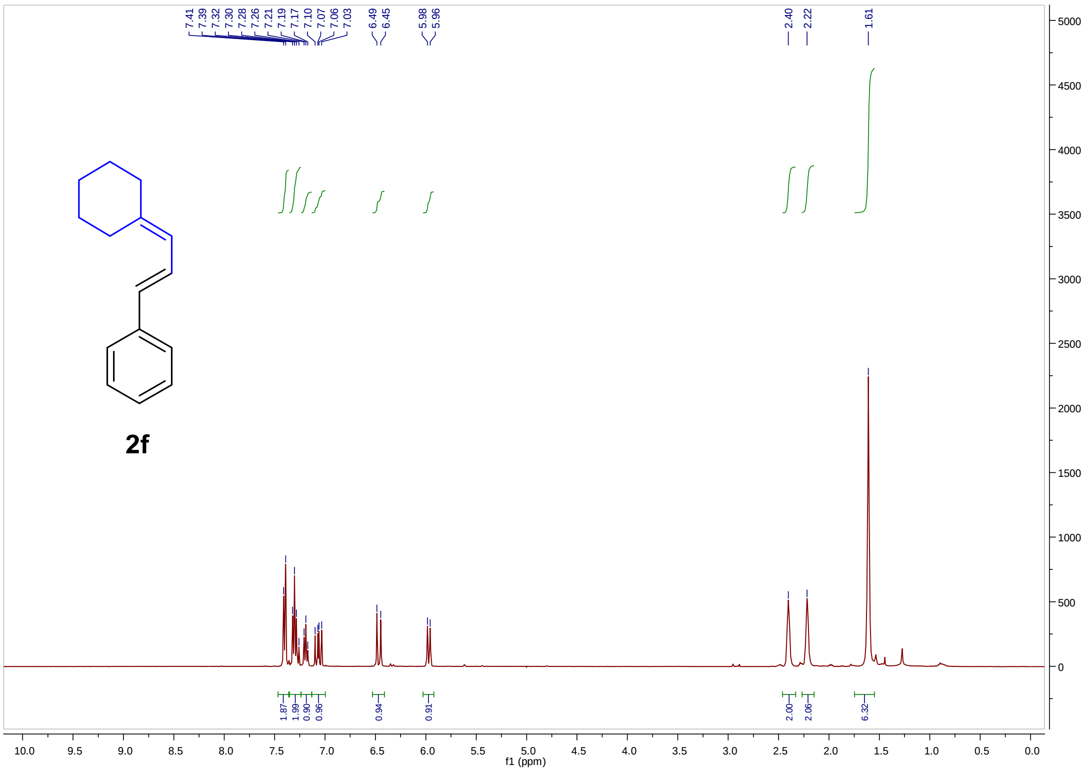
7.41 7.39 7.32 7.30 7.28 7.26 7.21 7.19 7.17 7.10 7.07 7.06 7.03
6.49 6.45
5.98 5.96
2.40
2.22
1.61
2f
1.87 1.99 0.90 0.96
0.94
0.91
2.00
2.06
6.32
f1 (ppm)

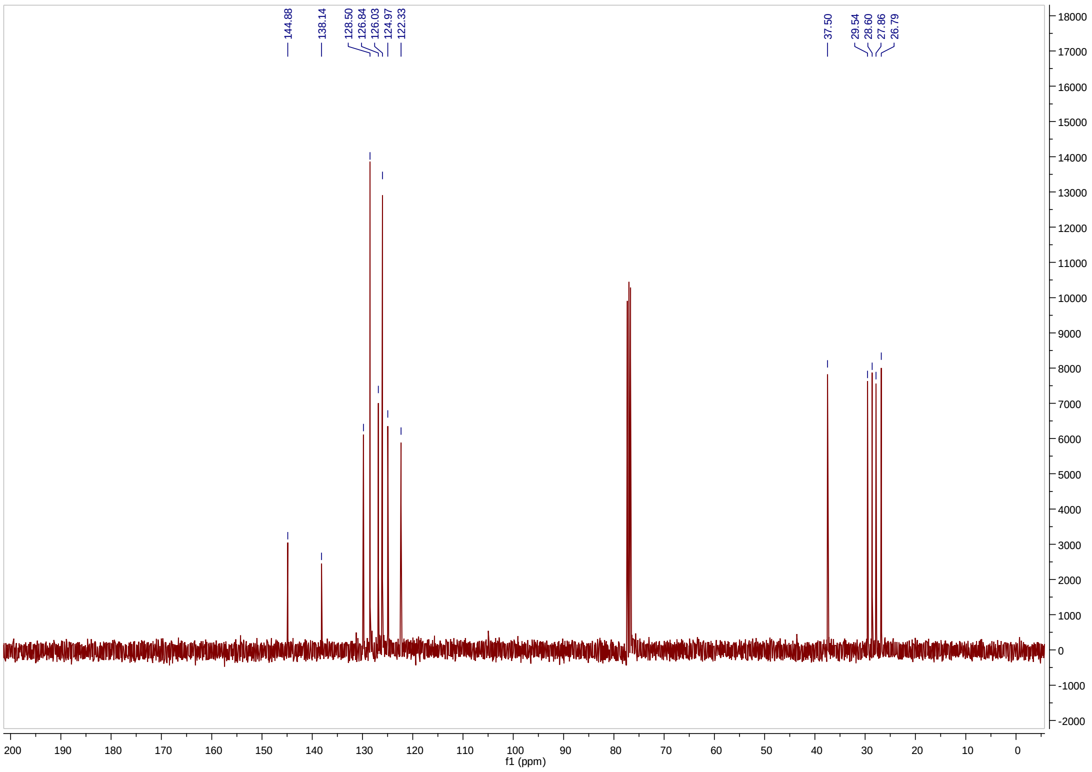
144.88
138.14
128.50 126.84 126.03 124.97 122.33
37.50
29.54 28.60 27.86 26.79
f1 (ppm)

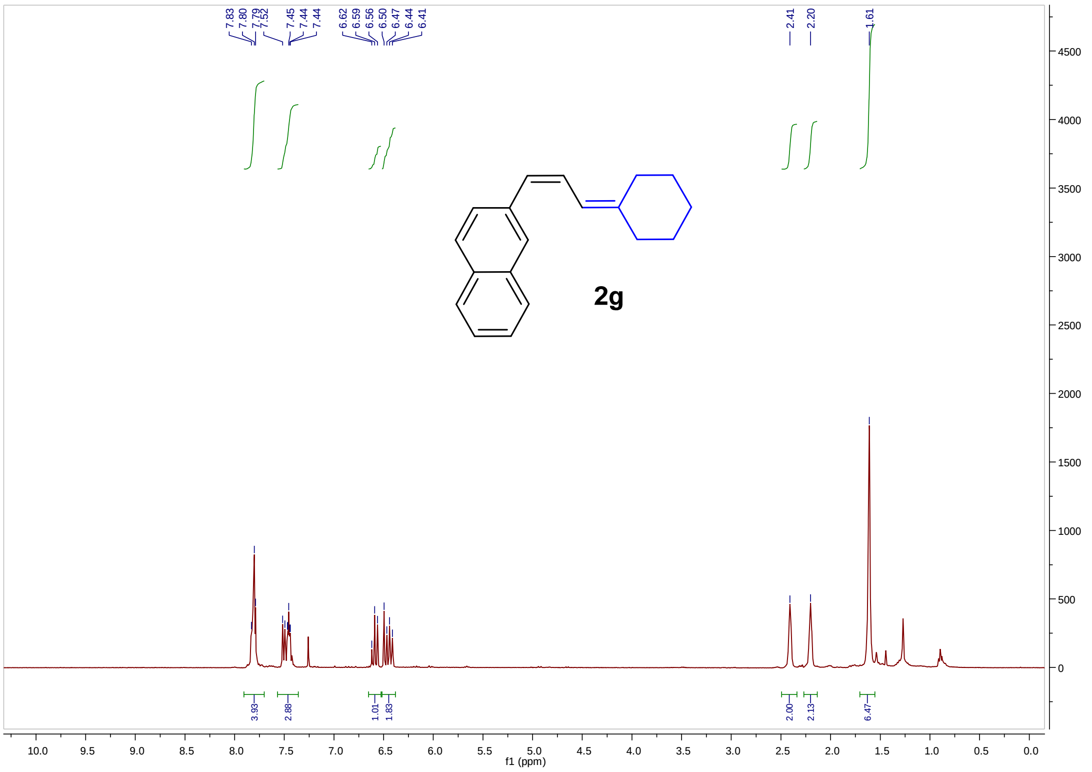
7.83
7.80
7.79
7.52
7.45
7.44
7.44
6.62
6.59
6.56
6.50
6.47
6.44
6.41
2.41
2.20
1.61
2g
3.93
2.88
1.01
1.83
2.00
2.13
6.47
f1 (ppm)

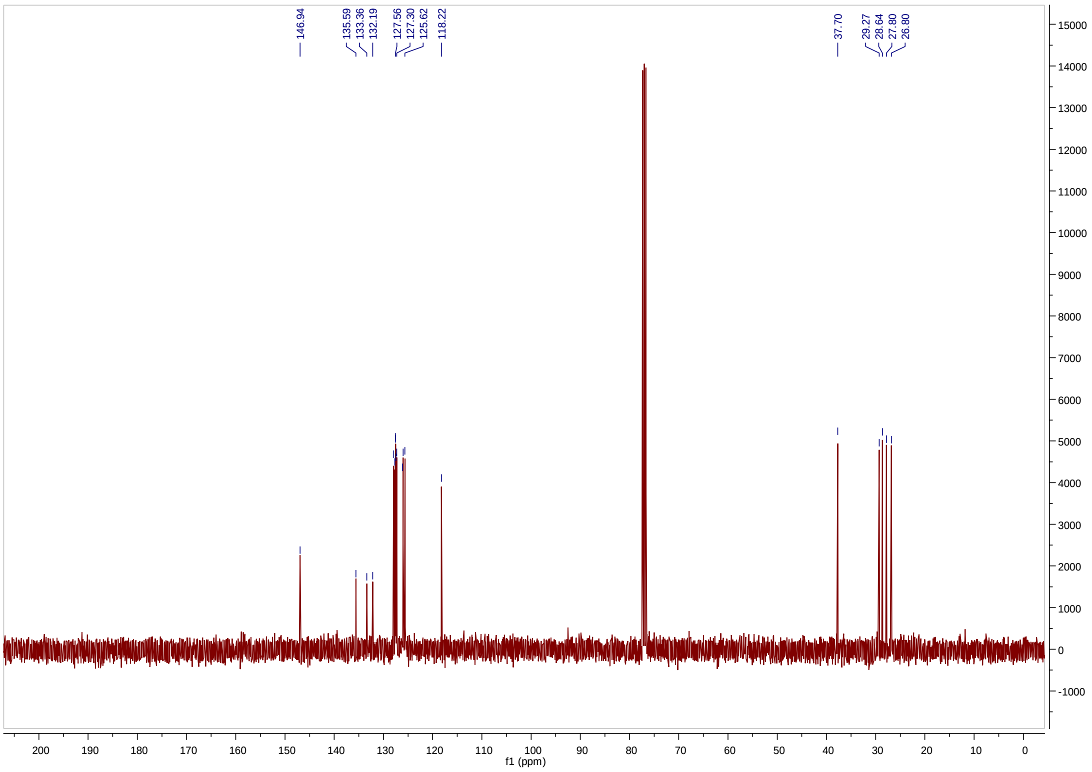
146.94
135.59
133.36
132.19
127.56
127.30
125.62
118.22
37.70
29.27
28.64
27.80
26.80
f1 (ppm)

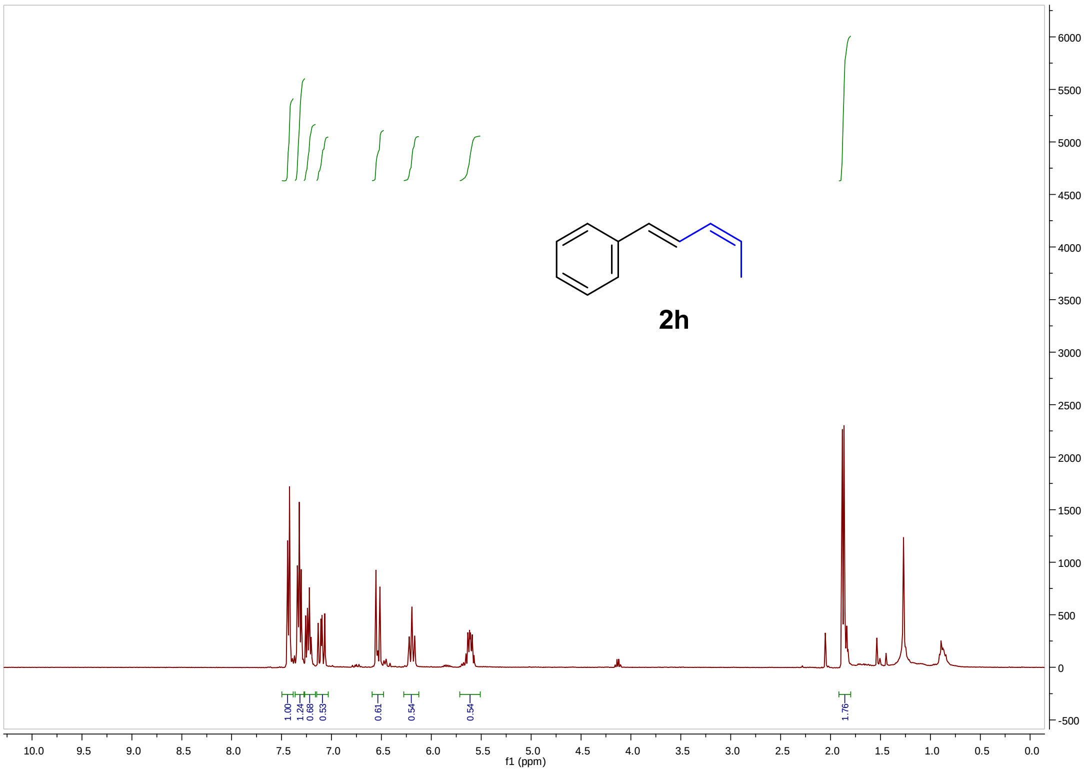
2h
1.00
1.24
0.68
0.53
0.61
0.54
0.54
1.76
f1 (ppm)

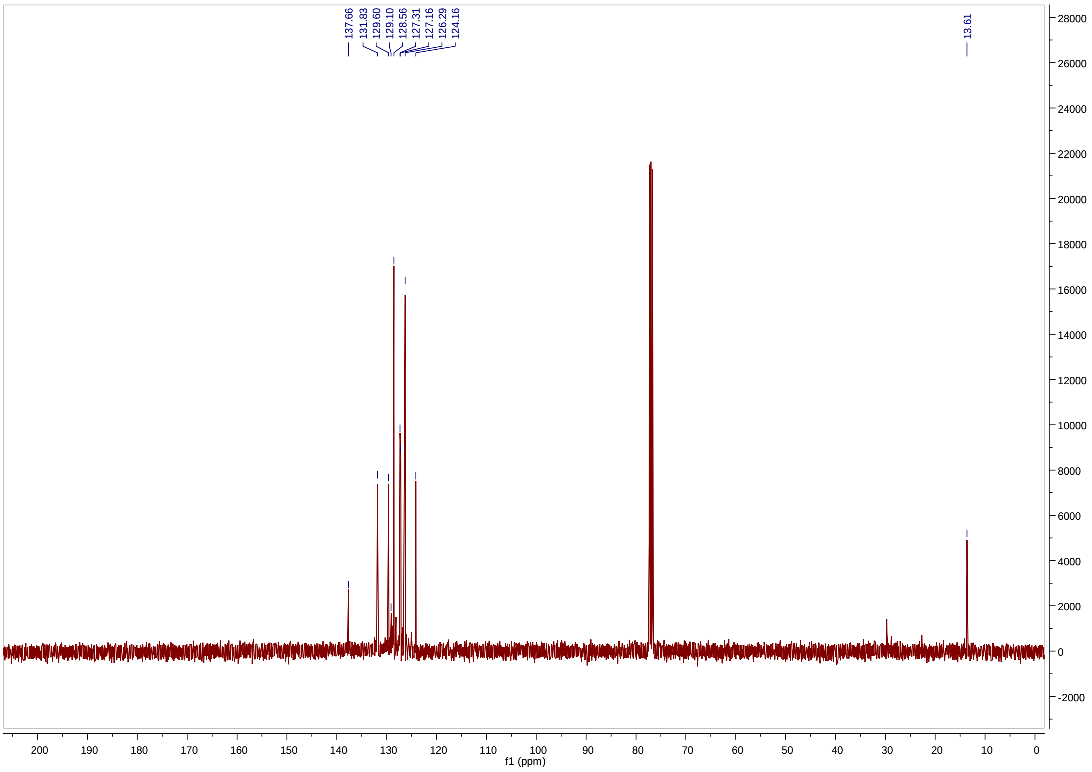
137.66
131.83
129.60
129.10
128.56
127.31
127.16
126.29
124.16
13.61
f1 (ppm)

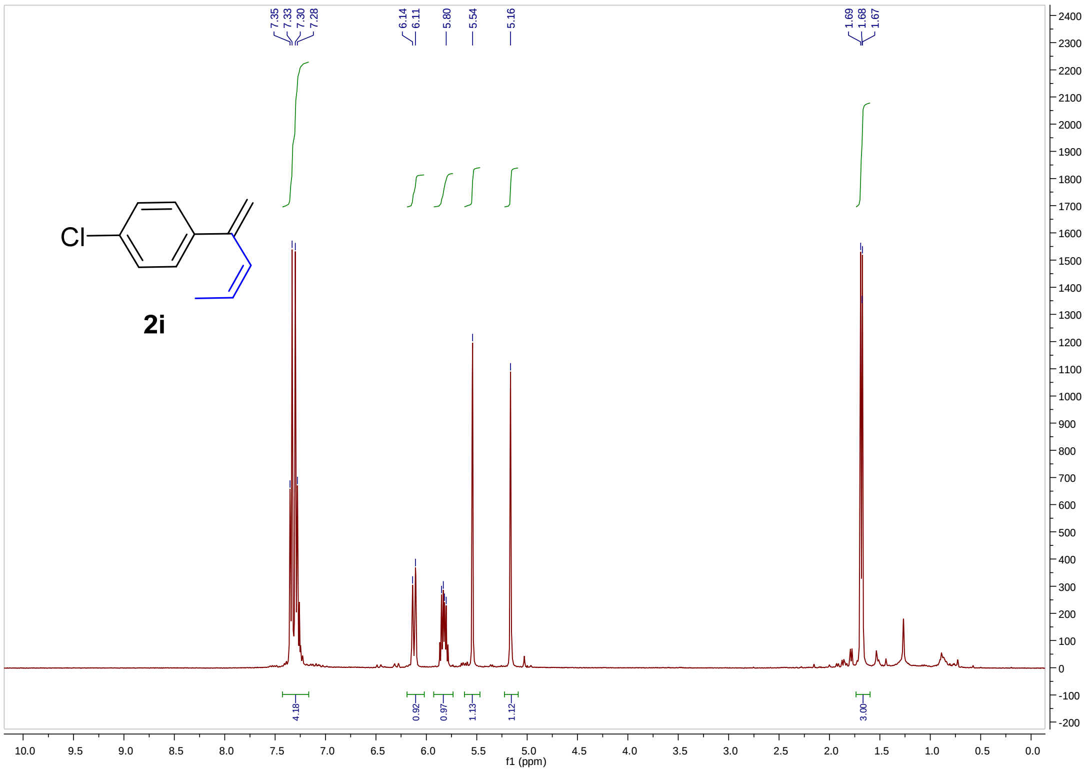

7.35
7.33
7.30
7.28
6.14
6.11
5.80
5.54
5.16
1.69
1.68
1.67
Cl
2i
4.18
0.92
0.97
1.13
1.12
3.00
f1 (ppm)

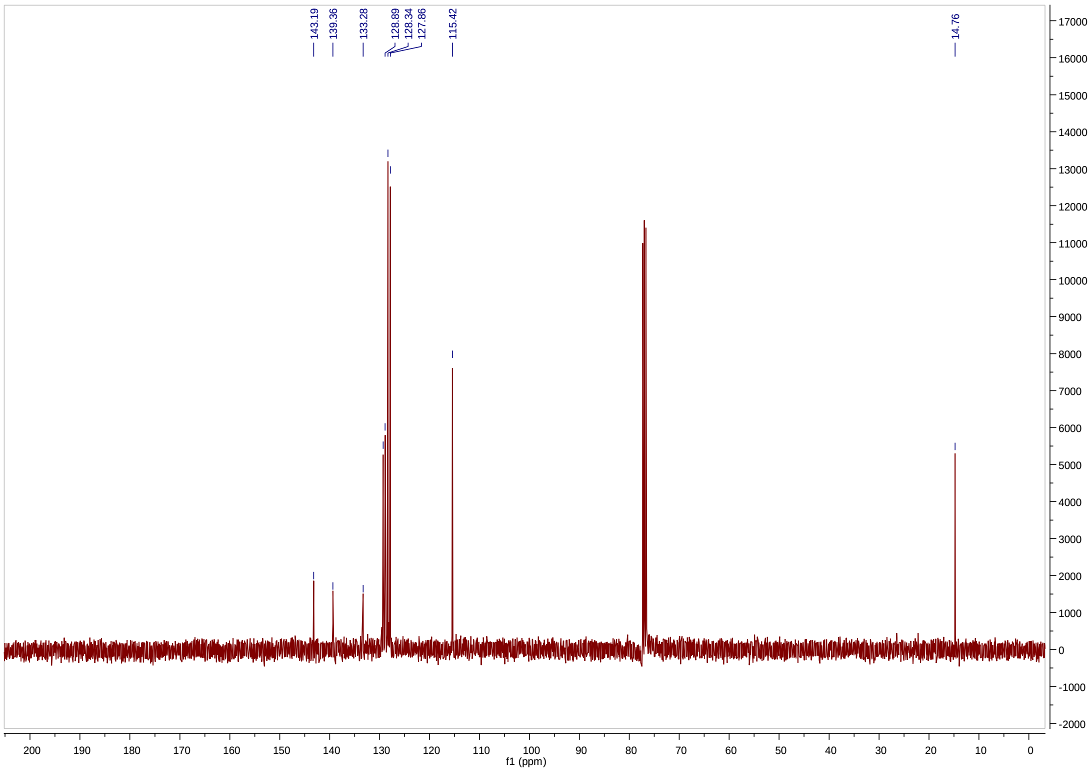

143.19
139.36
133.28
128.89
128.34
127.86
115.42
14.76
f1 (ppm)

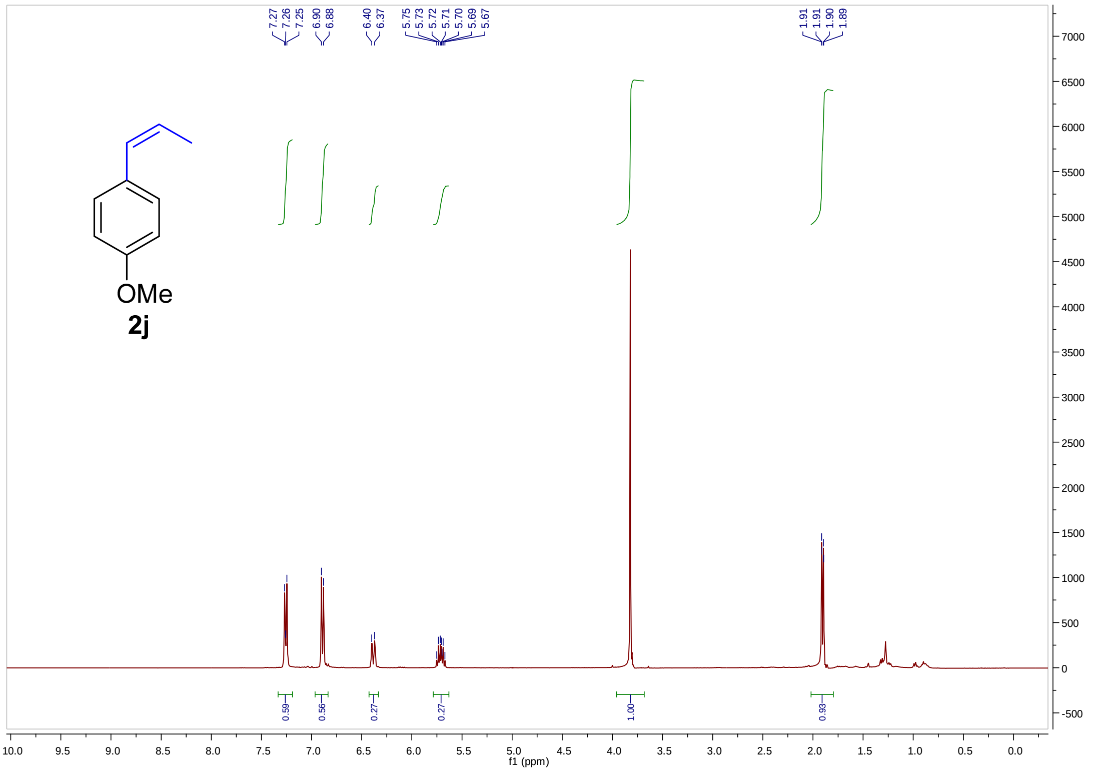
7.27
7.26
7.25
6.90
6.88
6.40
6.37
5.75
5.73
5.72
5.71
5.70
5.69
5.67
1.91
1.91
1.90
1.89
OMe
2j
0.59
0.56
0.27
0.27
1.00
0.93
f1 (ppm)

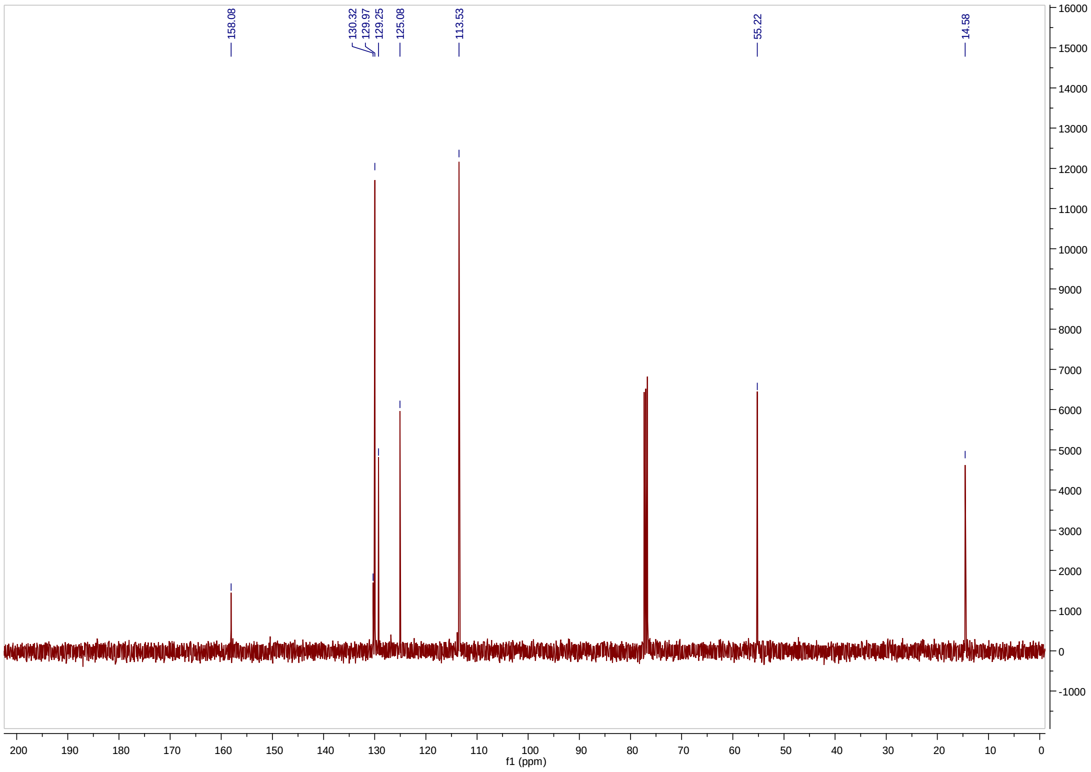
158.08
130.32
129.97
129.25
125.08
113.53
55.22
14.58
f1 (ppm)

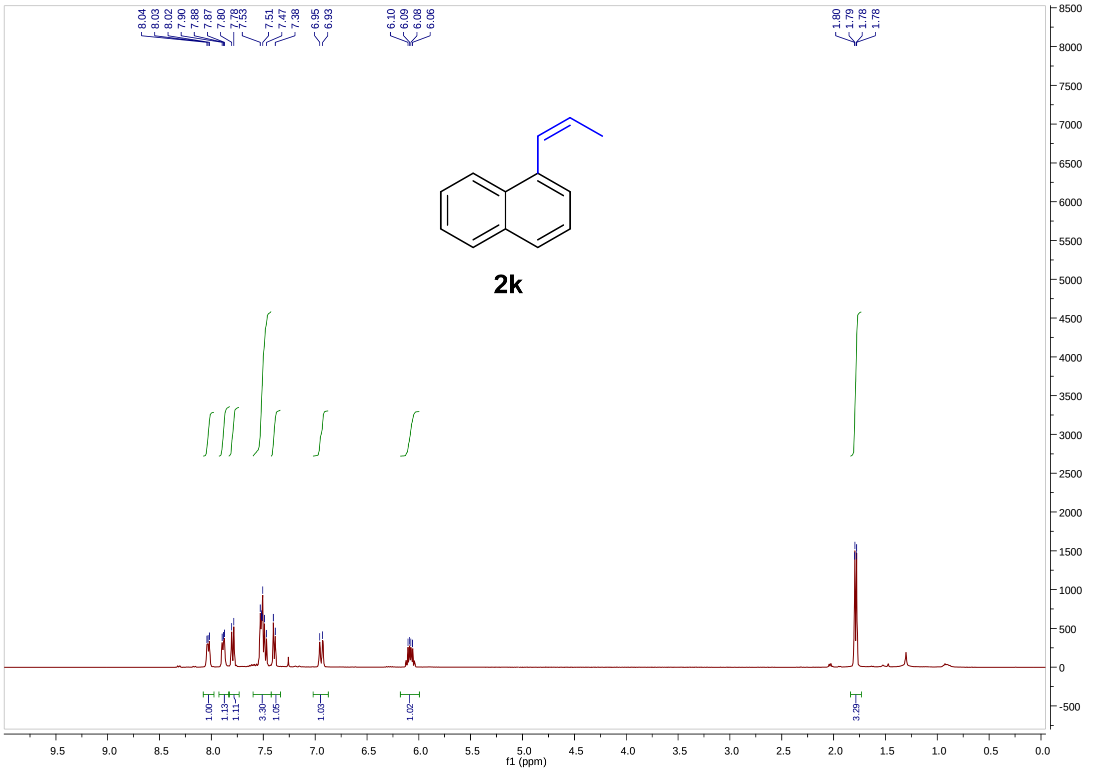
8.04
8.03
8.02
7.90
7.88
7.87
7.80
7.78
7.53
7.51
7.47
7.38
6.95
6.93
6.10
6.09
6.08
6.06
1.80
1.79
1.78
1.78
2k
1.00
1.13
1.11
3.30
1.05
1.03
1.02
3.29
f1 (ppm)

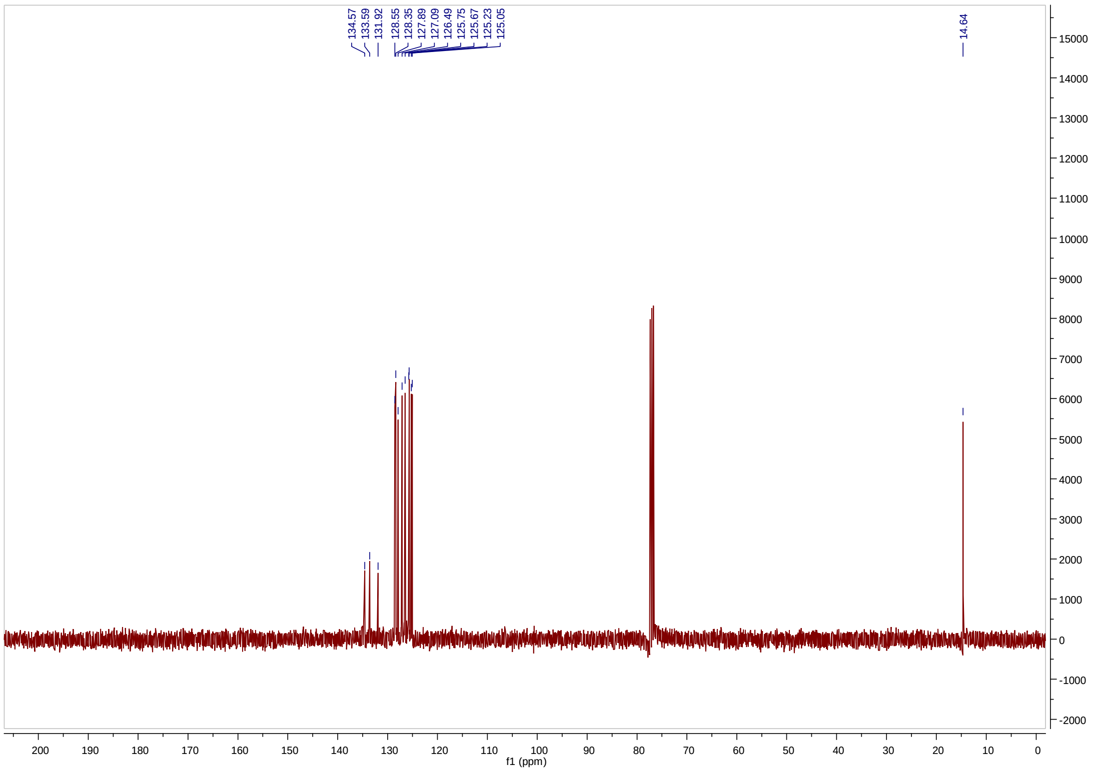
134.57
133.59
131.92
128.55
128.35
127.89
127.09
126.49
125.75
125.67
125.23
125.05
14.64
f1 (ppm)

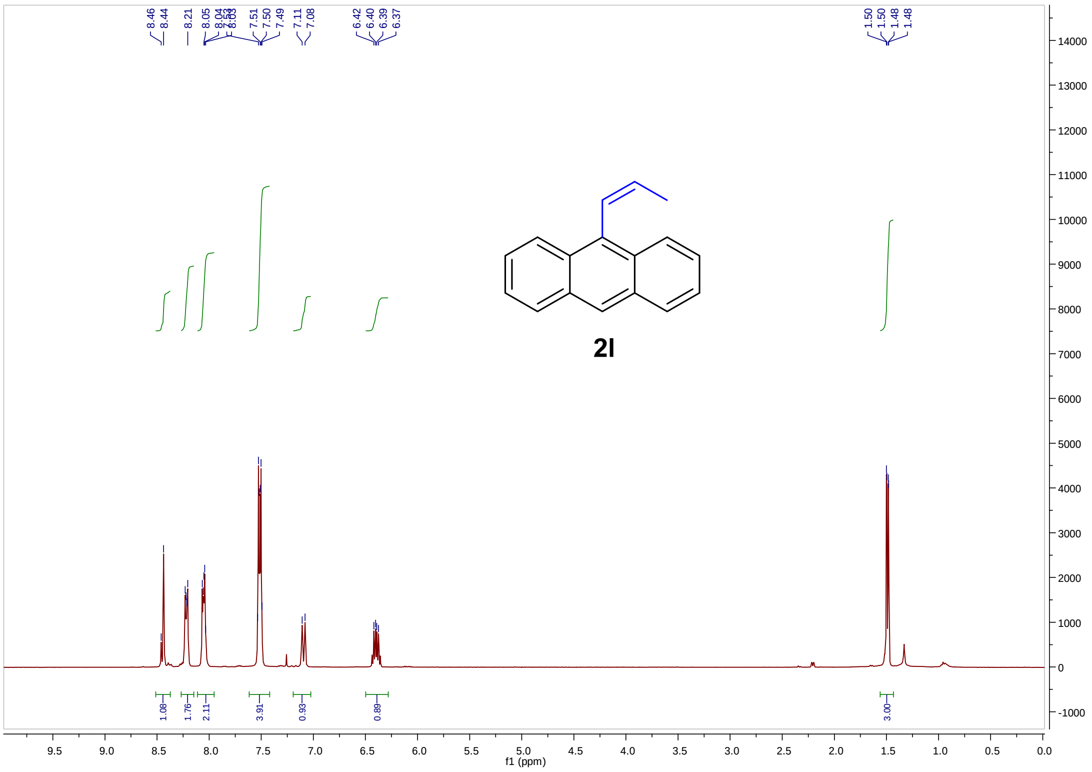
8.46
8.44
8.21
8.05
8.04
8.03
8.03
7.51
7.50
7.49
7.11
7.08
6.42
6.40
6.39
6.37
1.50
1.50
1.48
1.48
2l
1.08
1.76
2.11
3.91
0.93
0.89
3.00
f1 (ppm)

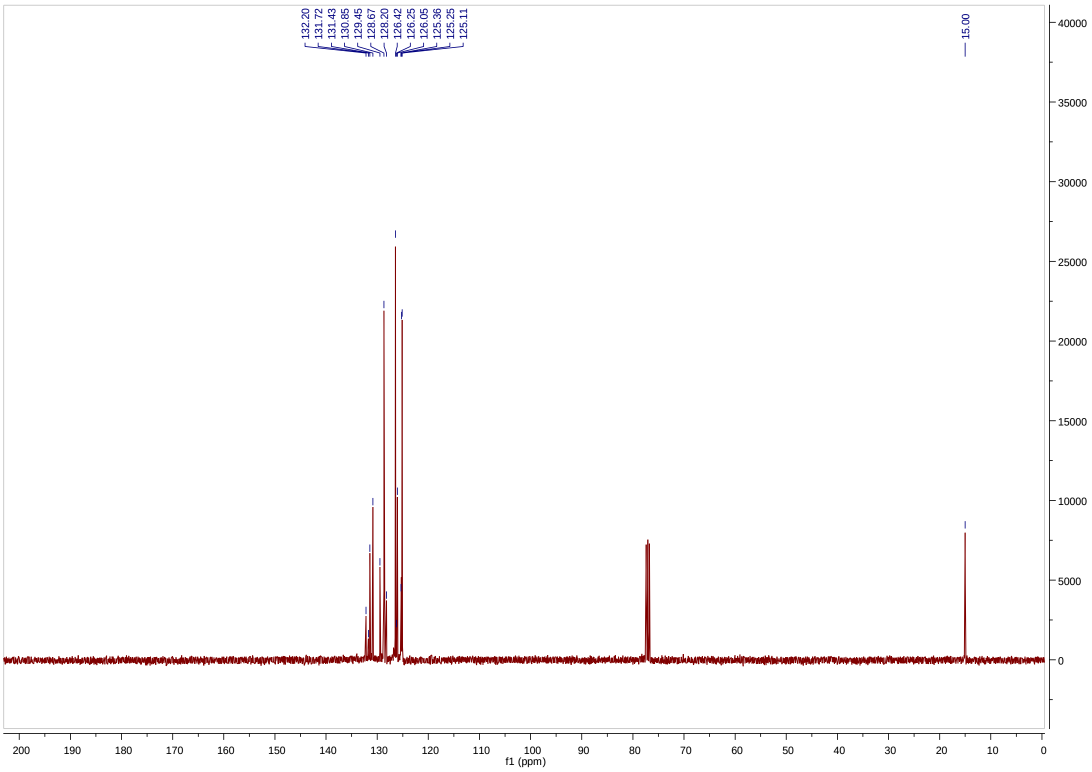
132.20
131.72
131.43
130.85
129.45
128.67
128.20
126.42
126.25
126.05
125.36
125.25
125.11
15.00
f1 (ppm)

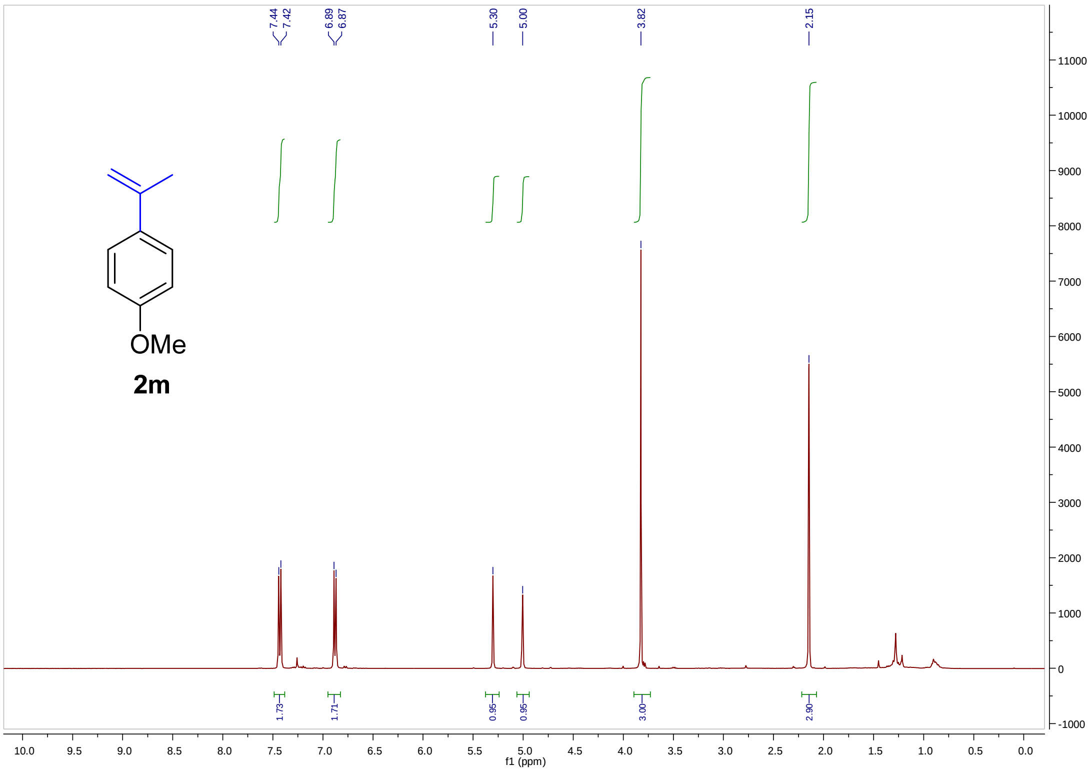
7.44
7.42
6.89
6.87
5.30
5.00
3.82
2.15
OMe
2m
1.73
1.71
0.95
0.95
3.00
2.90
f1 (ppm)

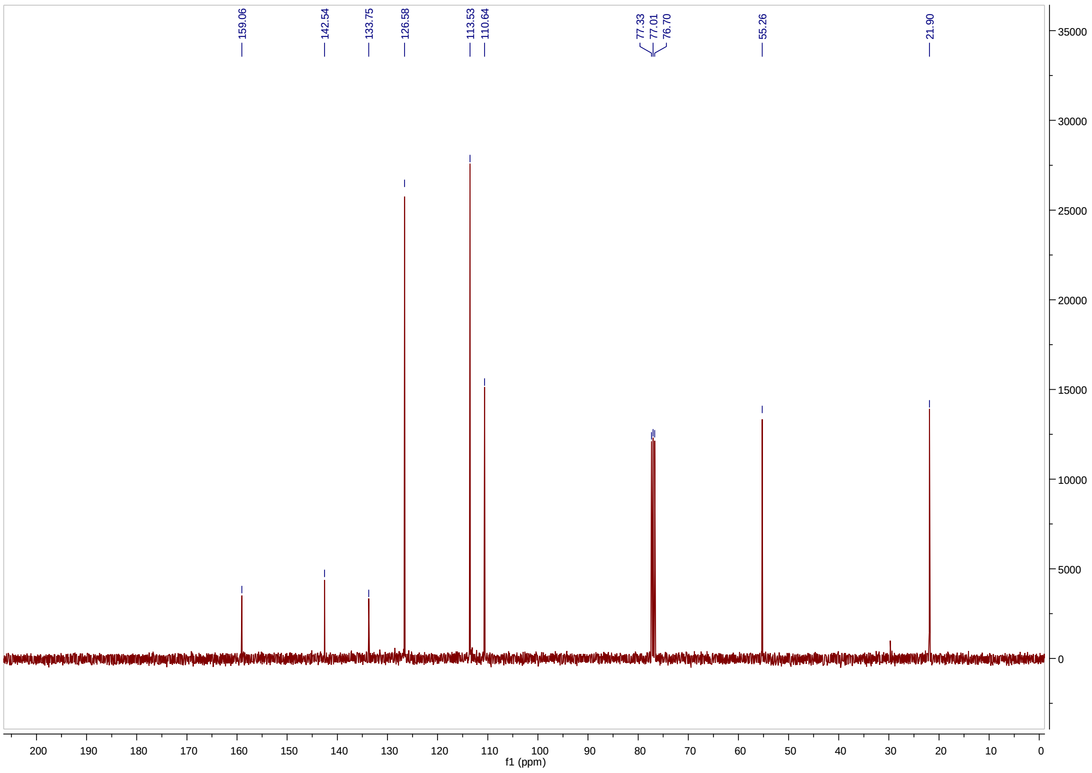
159.06
142.54
133.75
126.58
113.53
110.64
77.33
77.01
76.70
55.26
21.90
f1 (ppm)

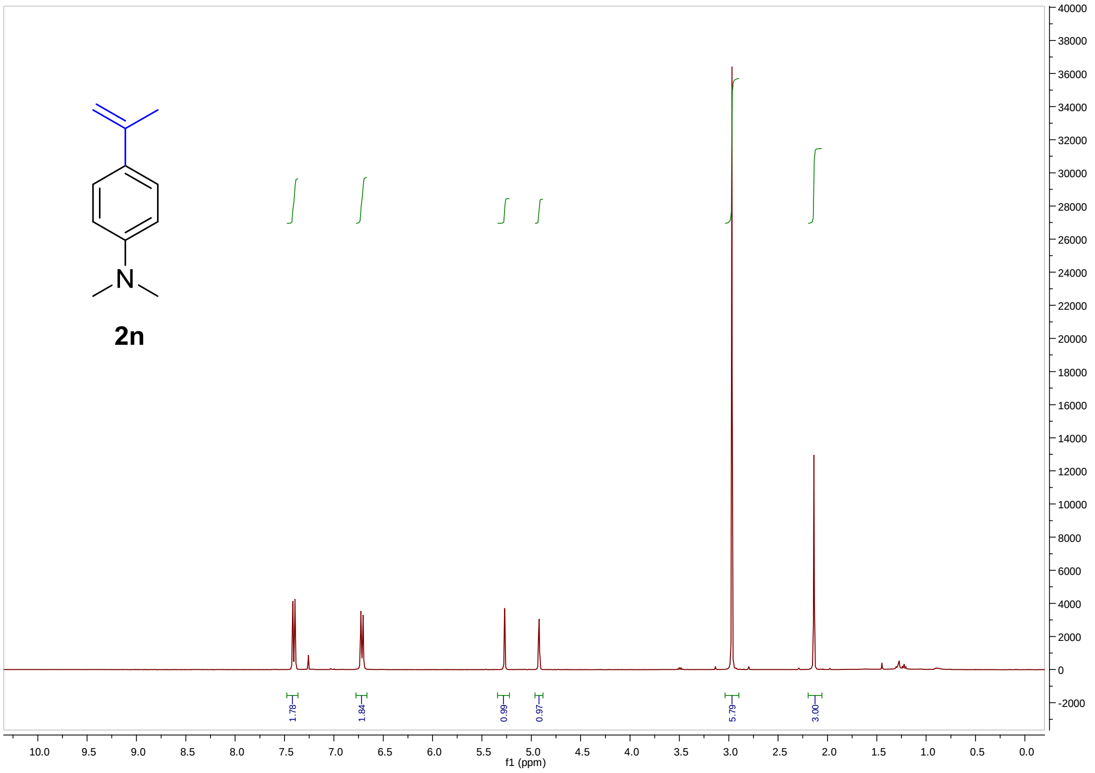

2n
1.78
1.84
0.99
0.97
5.79
3.00
f1 (ppm)

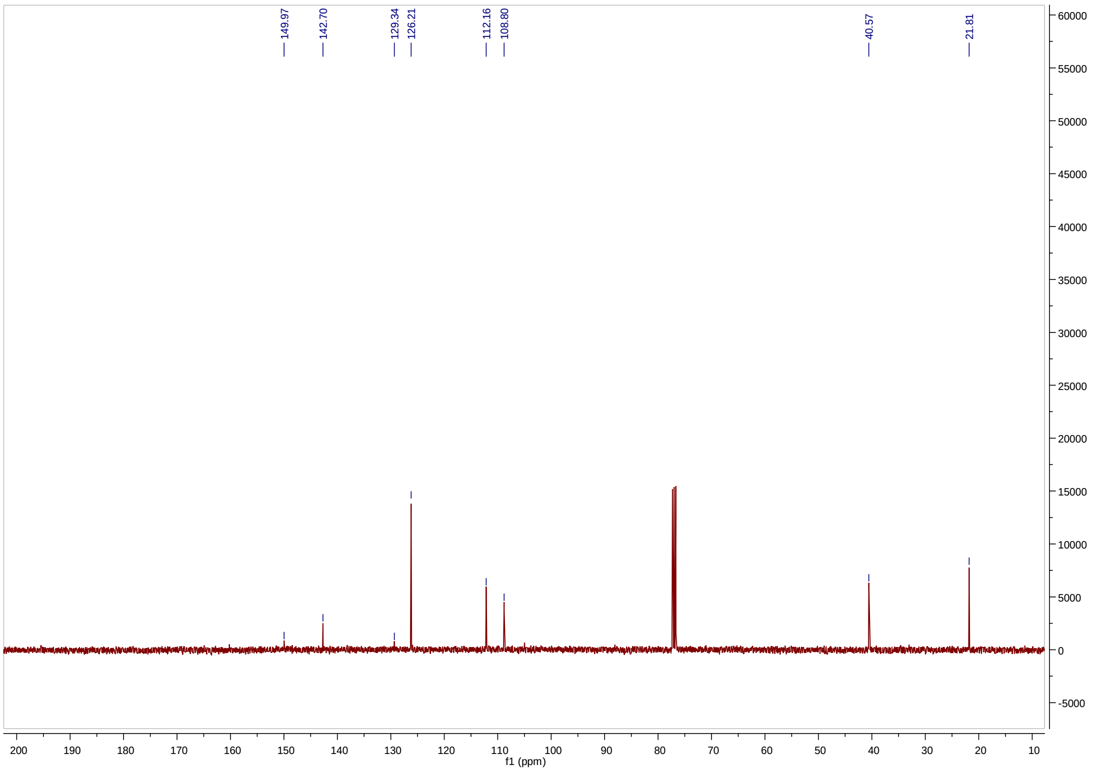

149.97
142.70
129.34
126.21
112.16
108.80
40.57
21.81
f1 (ppm)

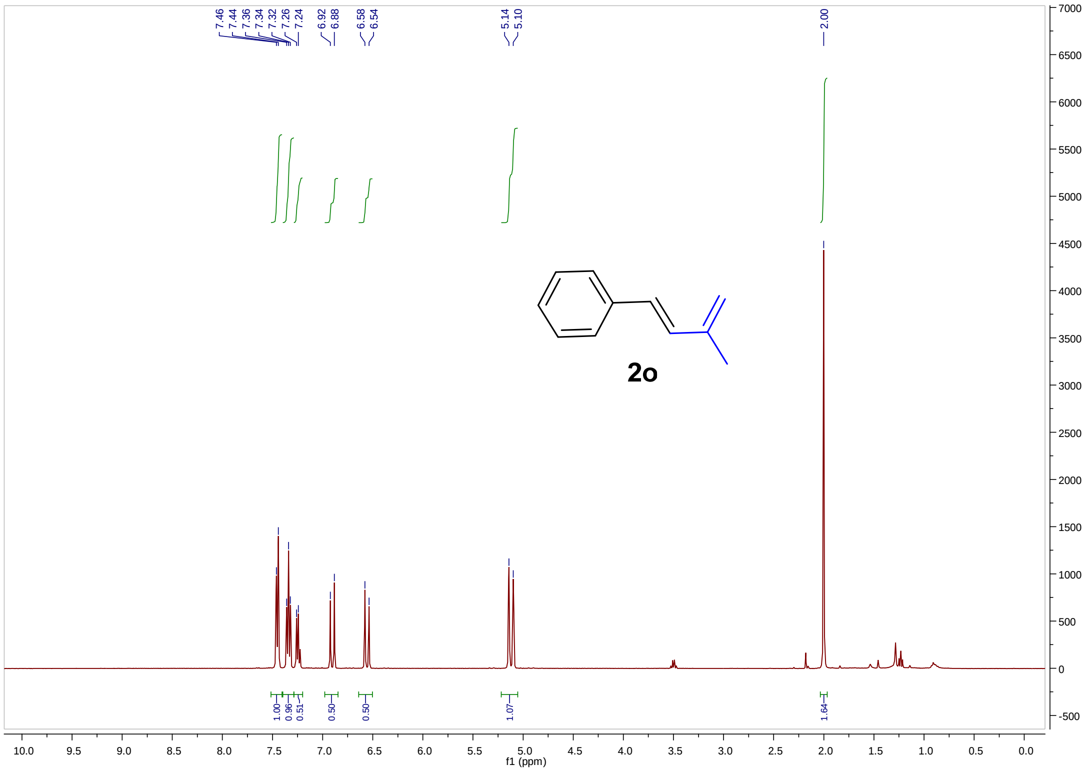

7.46
7.44
7.36
7.34
7.32
7.26
7.24
6.92
6.88
6.58
6.54
5.14
5.10
2.00
2o
1.00
0.96
0.51
0.50
0.50
1.07
1.64
f1 (ppm)

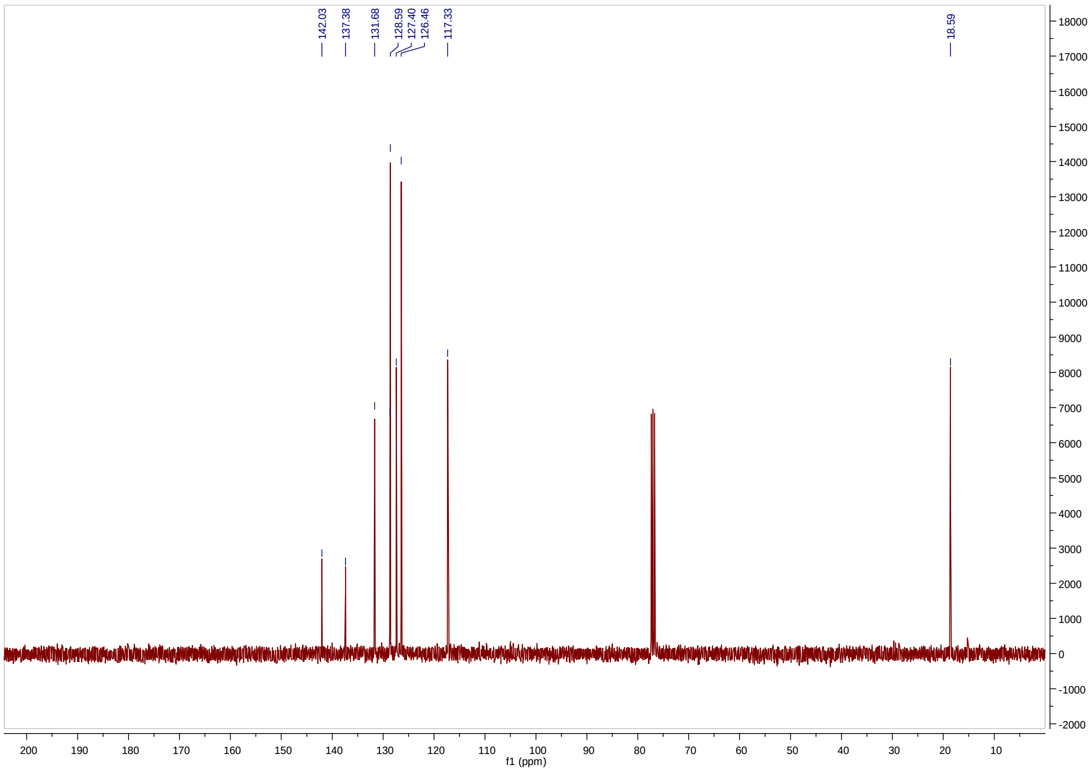

142.03
137.38
131.68
128.59
127.40
126.46
117.33
18.59
f1 (ppm)

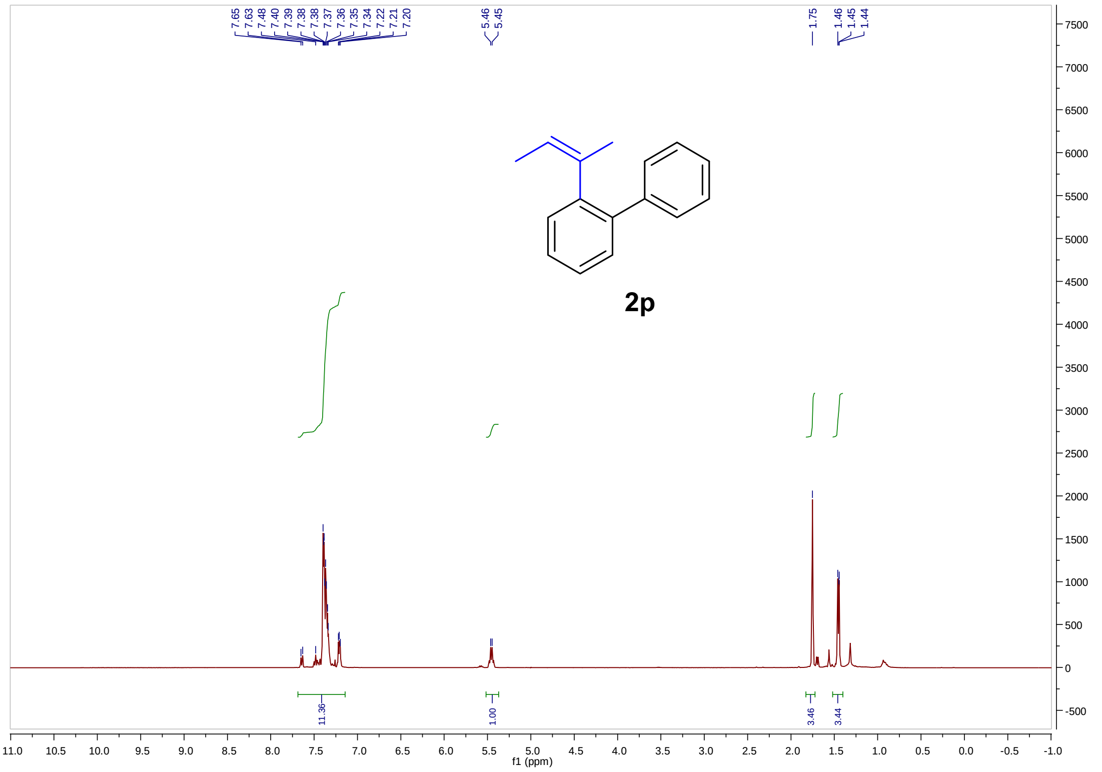
7.65
7.63
7.48
7.40
7.39
7.38
7.38
7.37
7.36
7.35
7.34
7.22
7.21
7.20
5.46
5.45
1.75
1.46
1.45
1.44
2p
11.36
1.00
3.46
3.44
f1 (ppm)

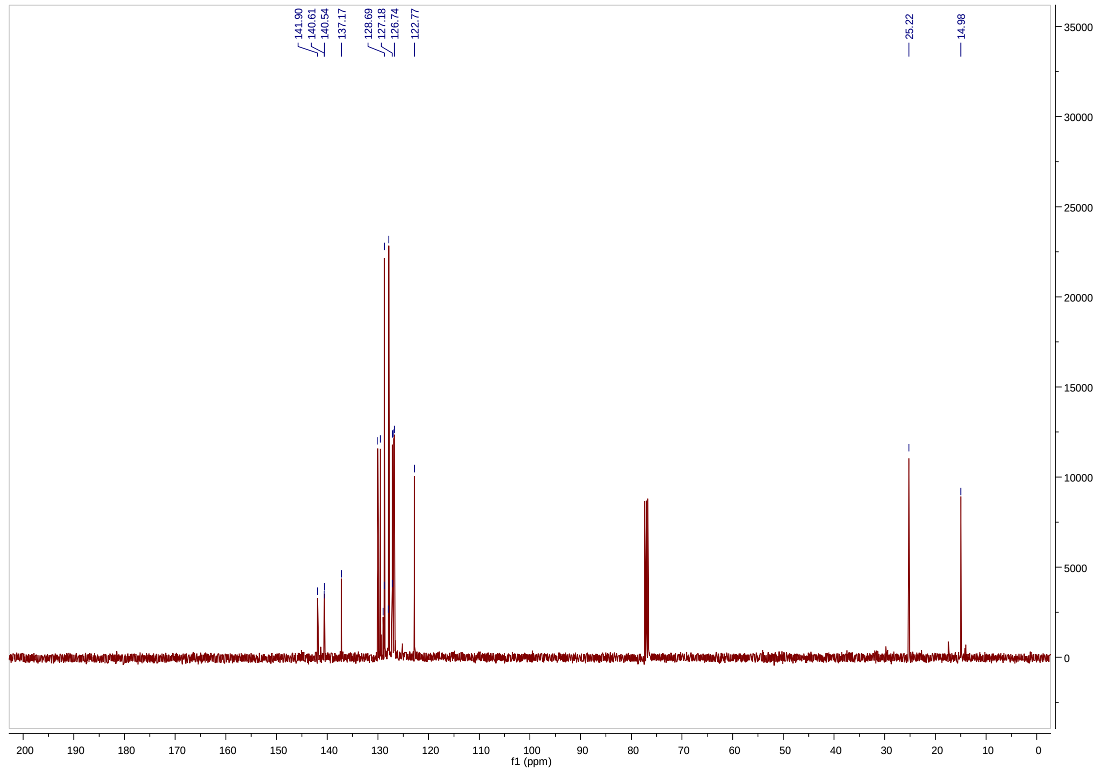
141.90
140.61
140.54
137.17
128.69
127.18
126.74
122.77
25.22
14.98
f1 (ppm)

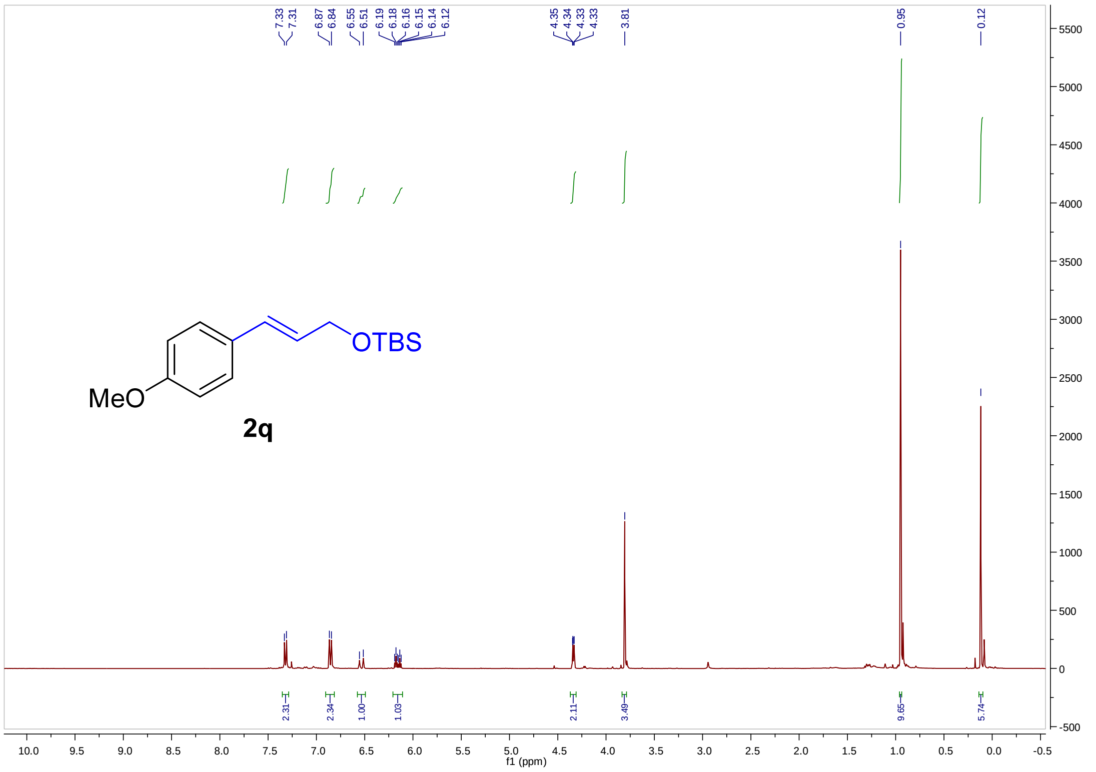
7.33
7.31
6.87
6.84
6.55
6.51
6.19
6.18
6.16
6.15
6.14
6.12
4.35
4.34
4.33
4.33
3.81
0.95
0.12
OTBS
MeO
2q
2.31
2.34
1.00
1.03
2.11
3.49
9.65
5.74
f1 (ppm)

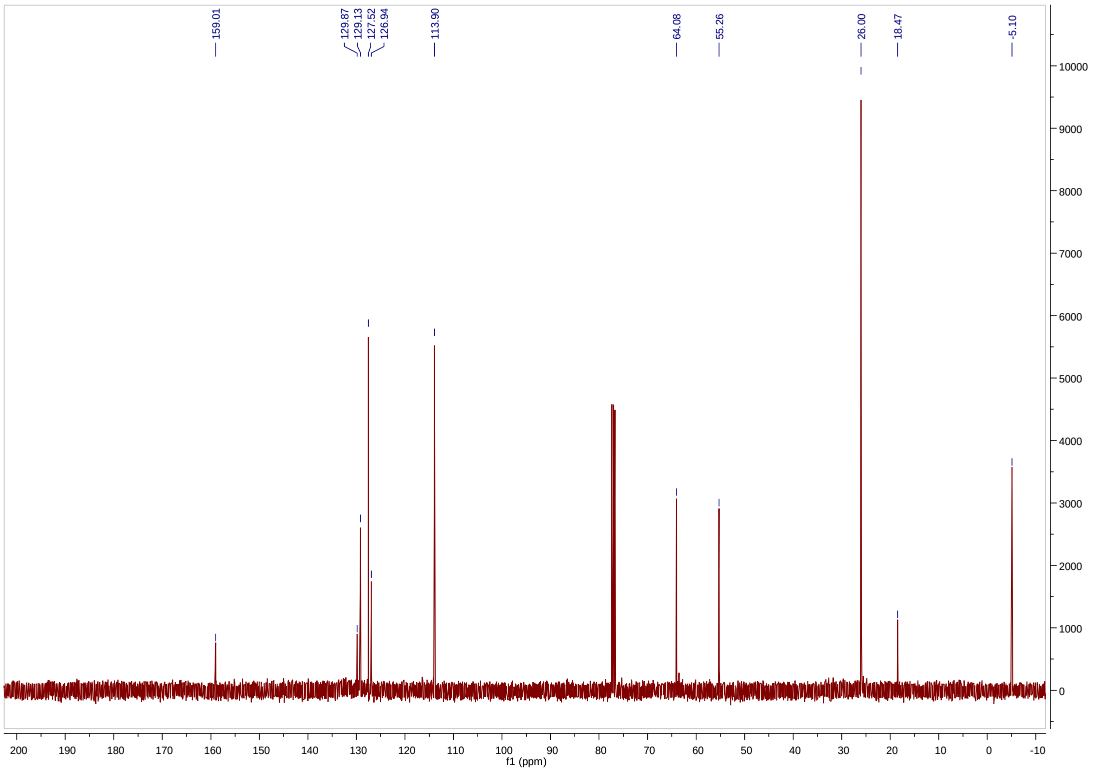
159.01
129.87
129.13
127.52
126.94
113.90
64.08
55.26
26.00
18.47
-5.10
f1 (ppm)

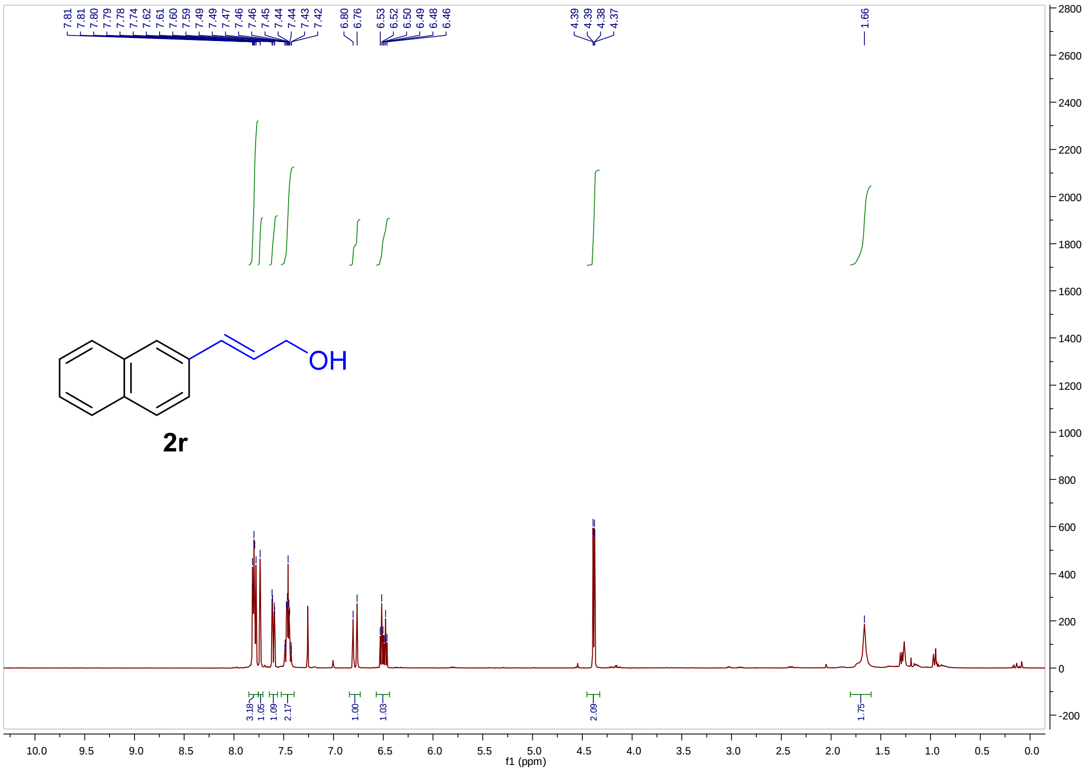
7.81
7.81
7.80
7.79
7.78
7.74
7.62
7.61
7.60
7.59
7.49
7.49
7.47
7.46
7.45
7.44
7.44
7.43
7.42
6.80
6.76
6.53
6.52
6.50
6.49
6.48
6.46
4.39
4.39
4.38
4.37
1.66
OH
2r
3.18
1.05
1.09
2.17
1.00
1.03
2.09
1.75
f1 (ppm)

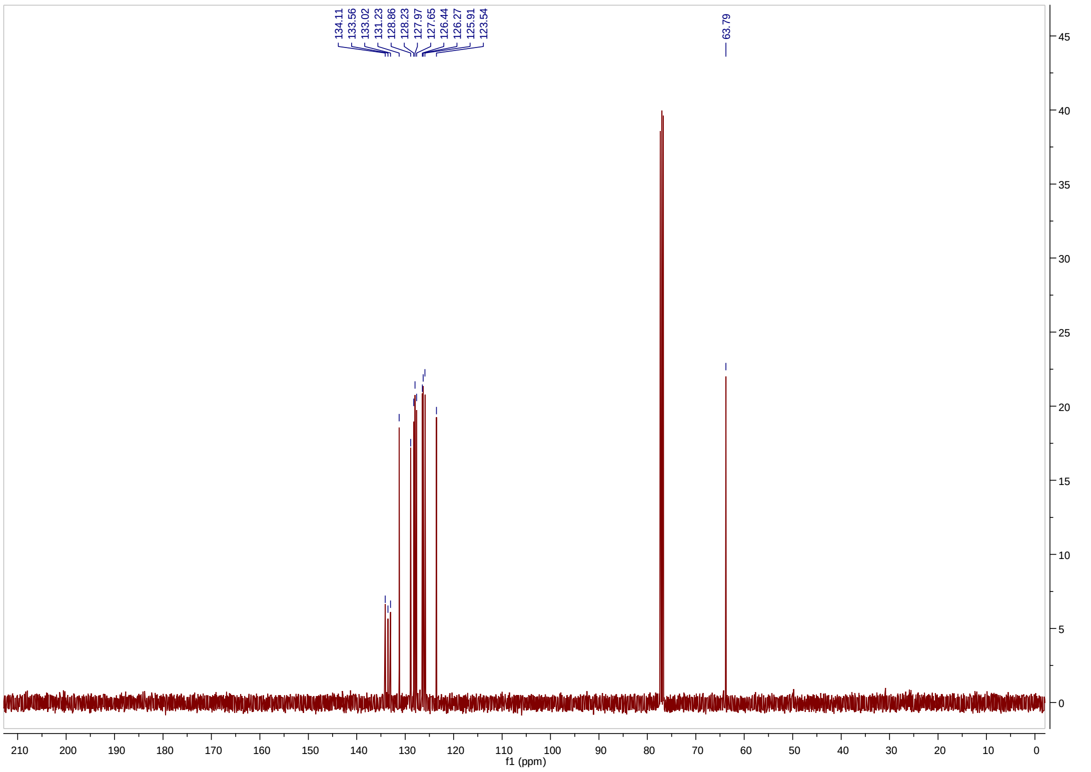
134.11
133.56
133.02
131.23
128.86
128.23
127.97
127.65
126.44
126.27
125.91
123.54
63.79
f1 (ppm)

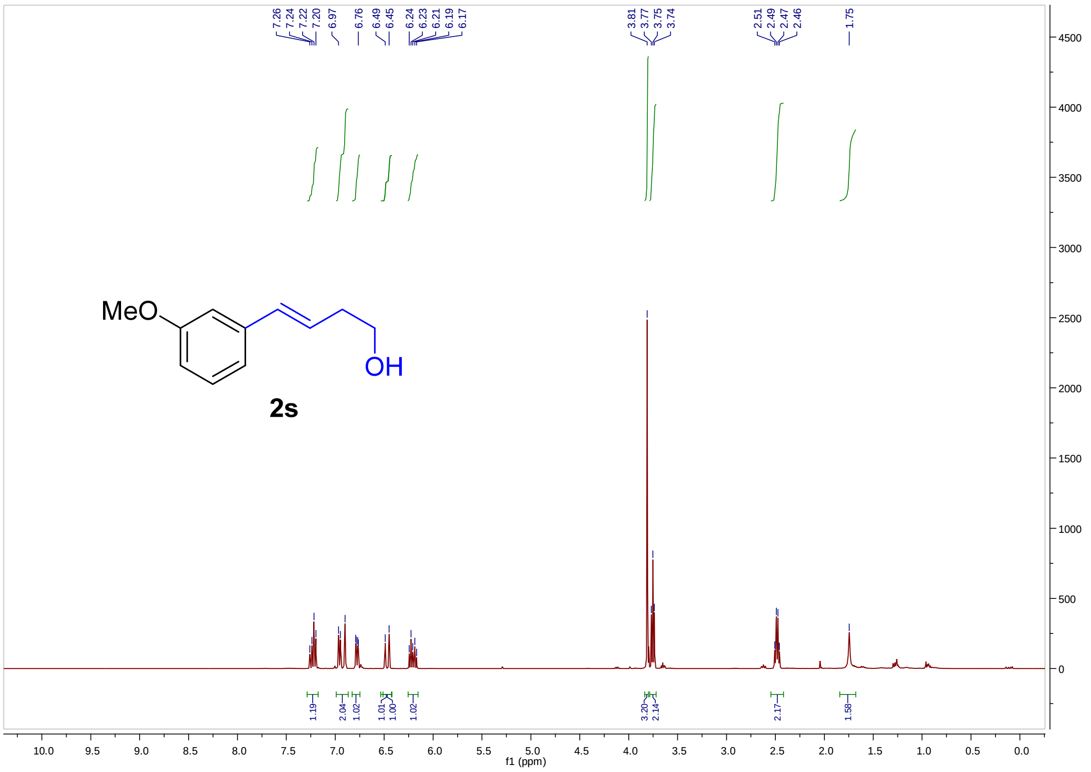

MeO
OH
2s
7.26
7.24
7.22
7.20
6.97
6.76
6.49
6.45
6.24
6.23
6.21
6.19
6.17
3.81
3.77
3.75
3.74
2.51
2.49
2.47
2.46
1.75
1.19
2.04
1.02
1.01
1.00
1.02
3.20
2.14
2.17
1.58
f1 (ppm)

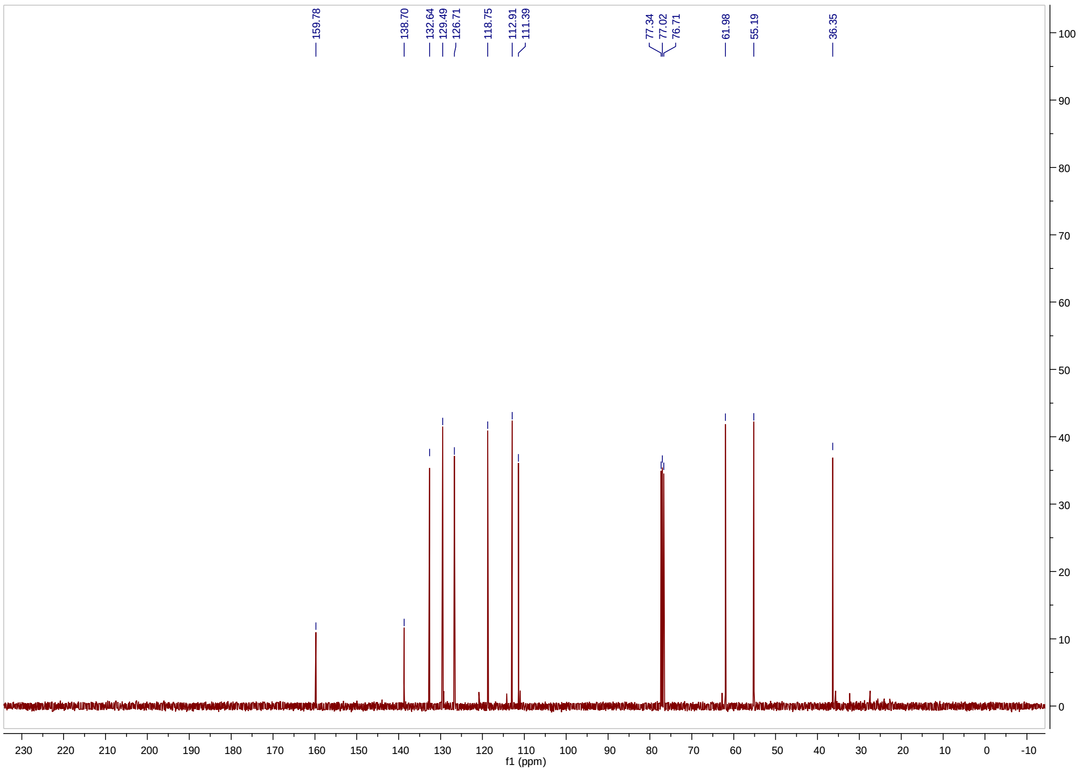

159.78
138.70
132.64
129.49
126.71
118.75
112.91
111.39
77.34
77.02
76.71
61.98
55.19
36.35
f1 (ppm)

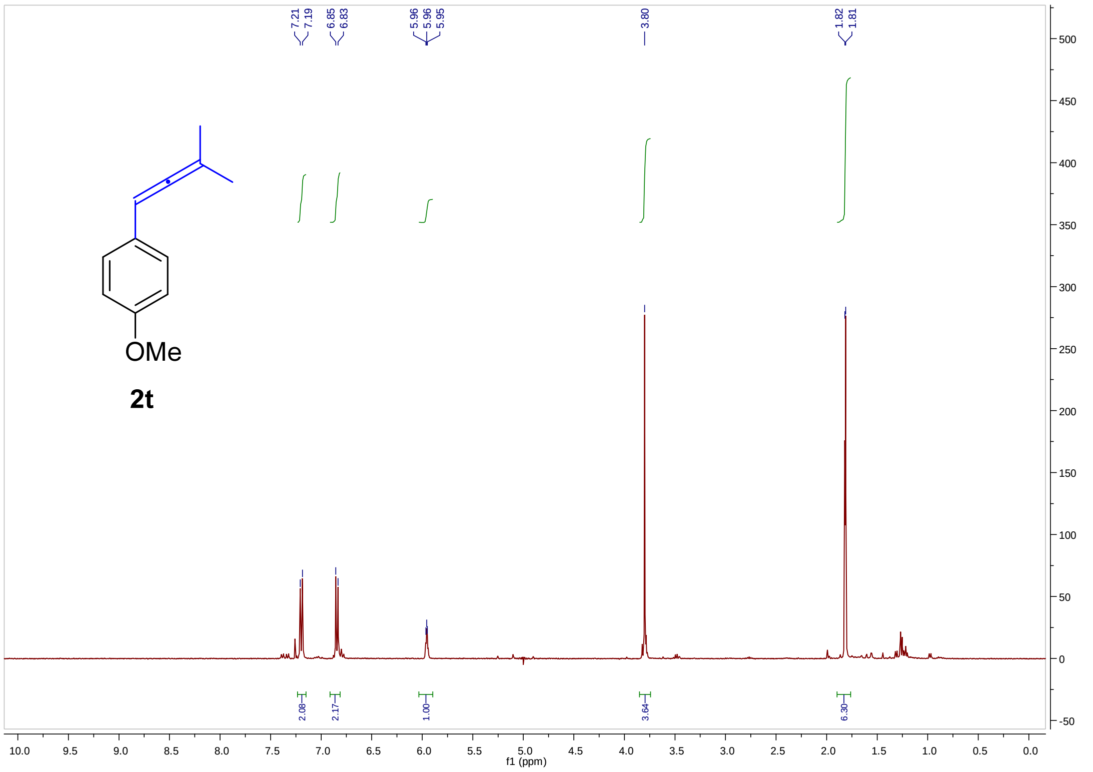
7.21
7.19
6.85
6.83
5.96
5.96
5.95
3.80
1.82
1.81
OMe
2t
2.08
2.17
1.00
3.64
6.30
f1 (ppm)

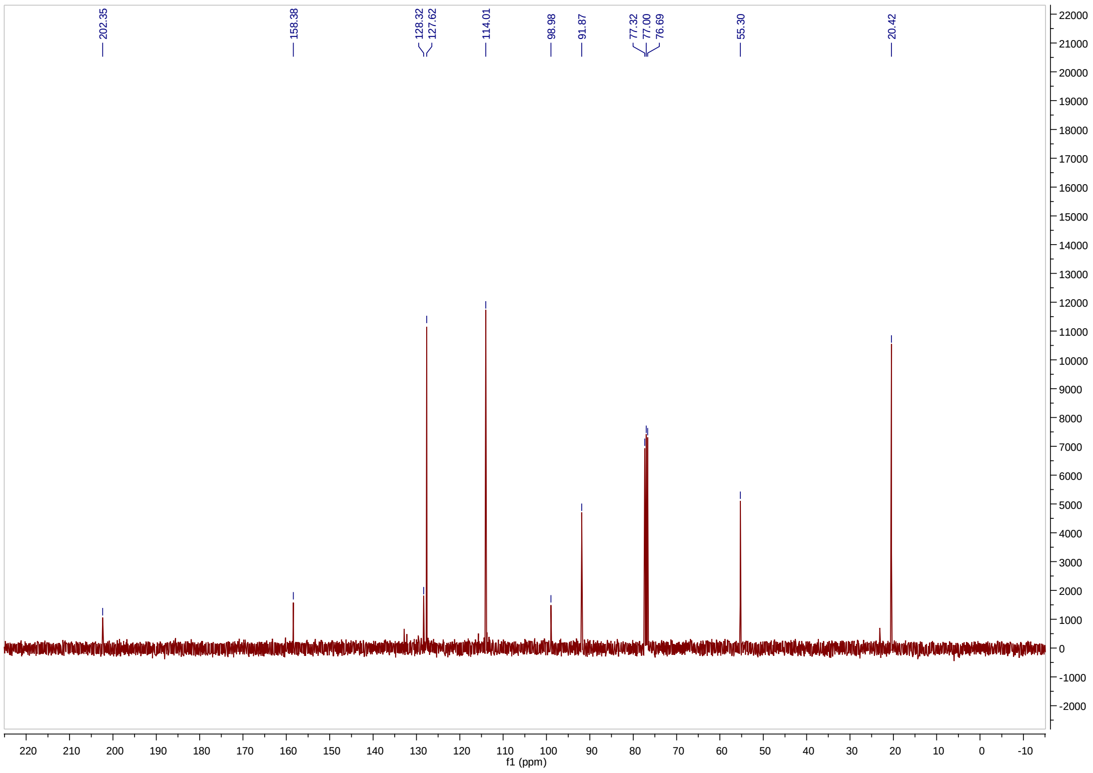
202.35
158.38
128.32
127.62
114.01
98.98
91.87
77.32
77.00
76.69
55.30
20.42
f1 (ppm)

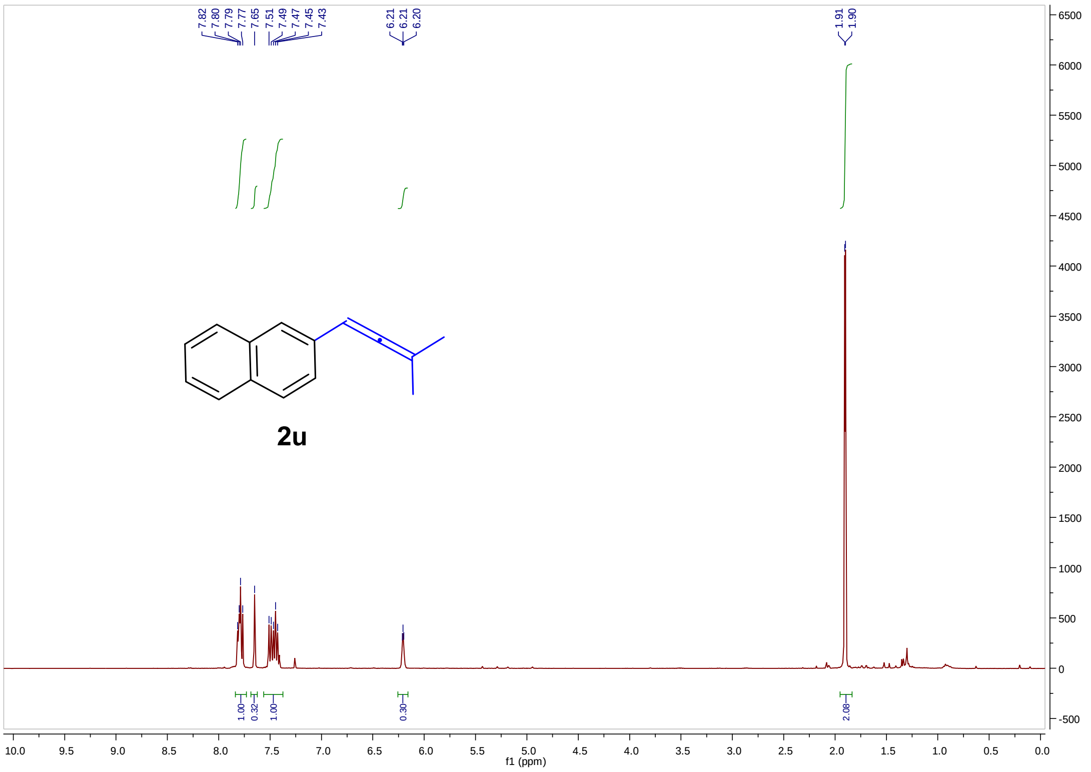
7.82
7.80
7.79
7.77
7.65
7.51
7.49
7.47
7.45
7.43
6.21
6.21
6.20
1.91
1.90
2u
1.00
0.32
1.00
0.30
2.08
f1 (ppm)

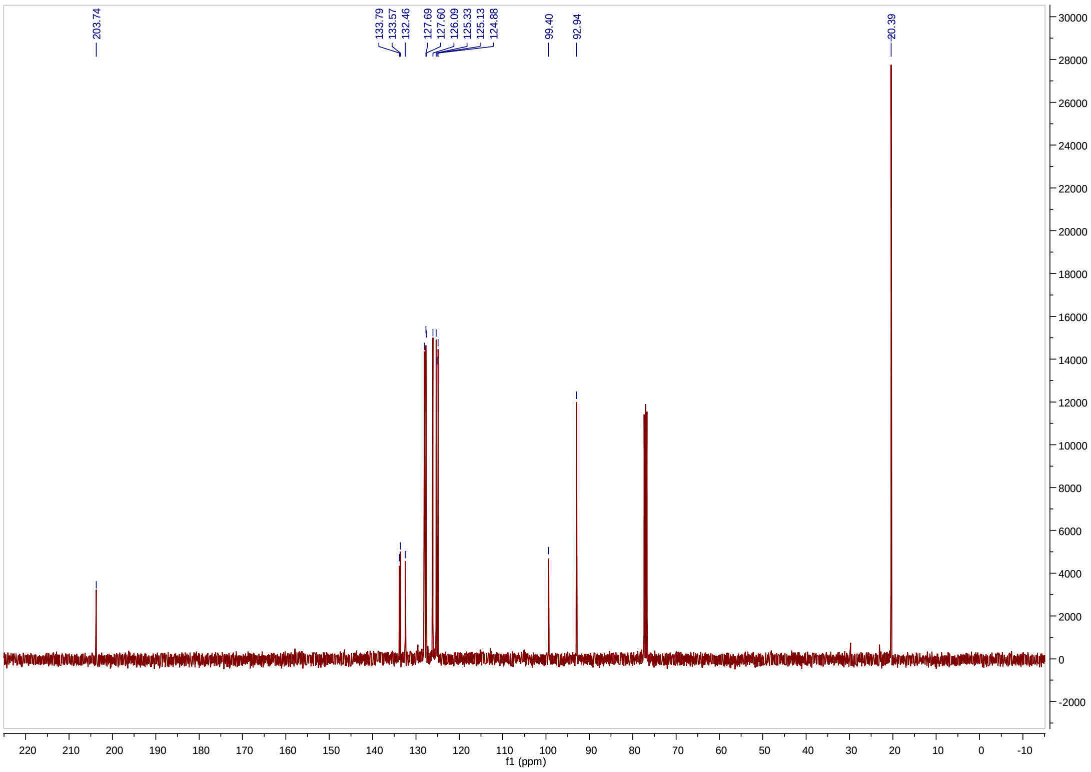
203.74
133.79
133.57
132.46
127.69
127.60
126.09
125.33
125.13
124.88
99.40
92.94
20.39
f1 (ppm)

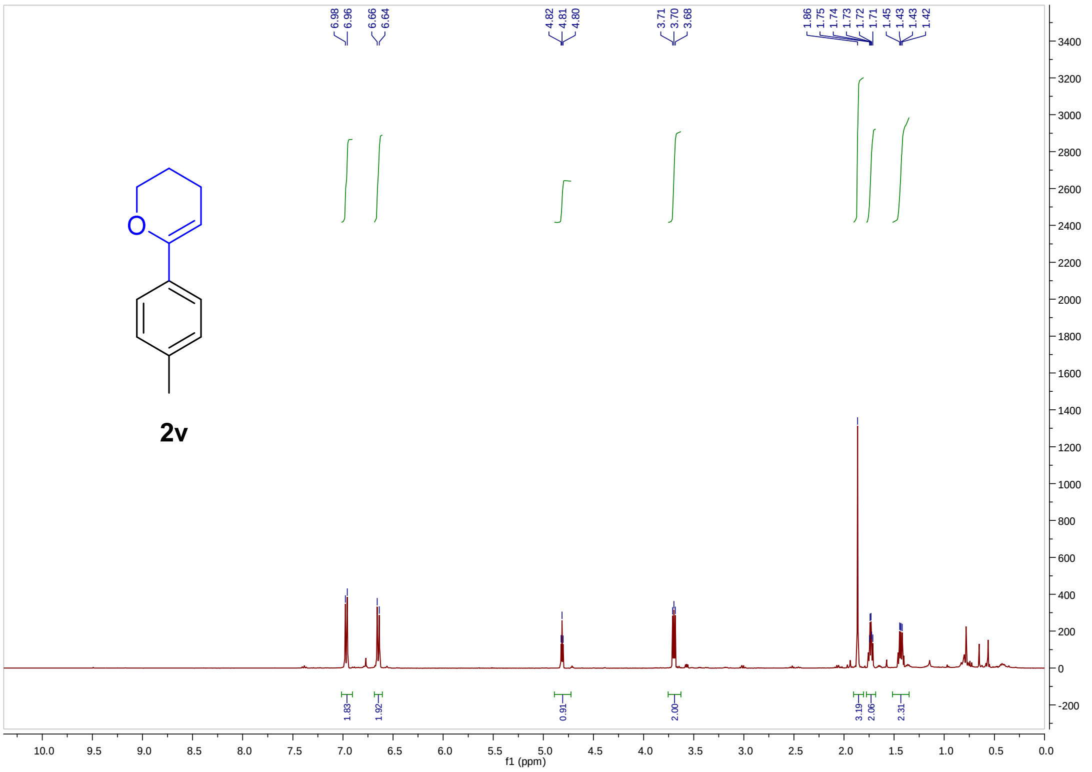
2v
6.98
6.96
6.66
6.64
4.82
4.81
4.80
3.71
3.70
3.68
1.86
1.75
1.74
1.73
1.72
1.71
1.45
1.43
1.43
1.42
1.83
1.92
0.91
2.00
3.19
2.06
2.31
f1 (ppm)

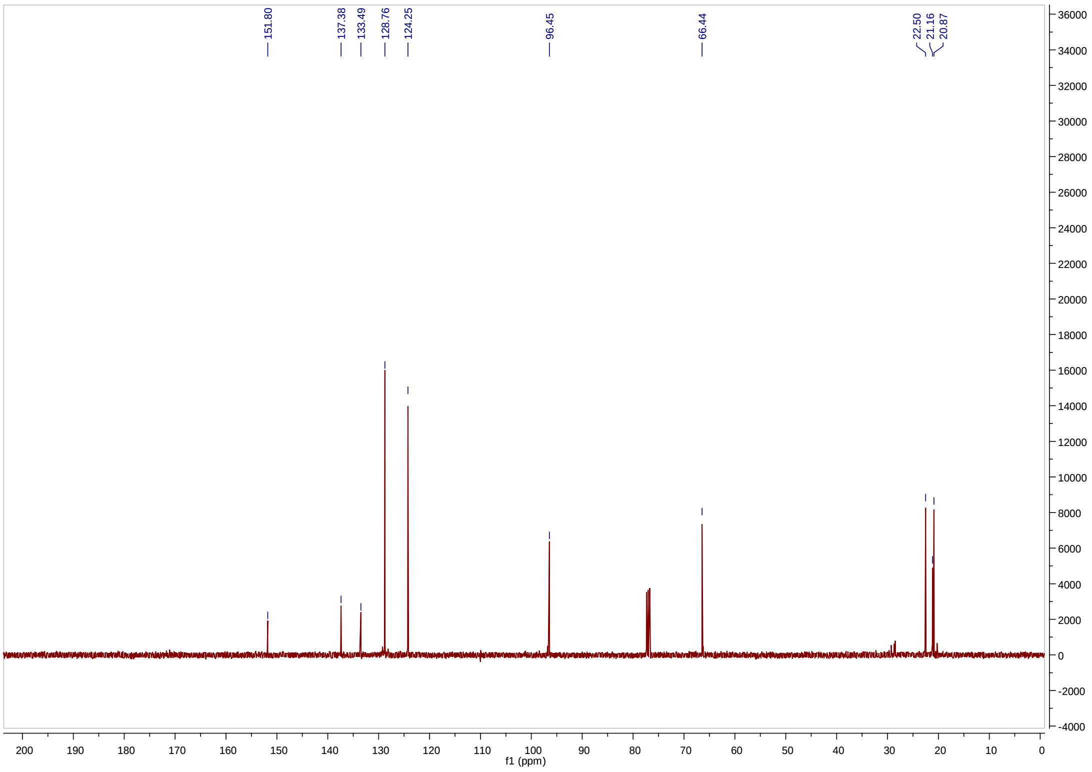
151.80
137.38
133.49
128.76
124.25
96.45
66.44
22.50
21.16
20.87
f1 (ppm)

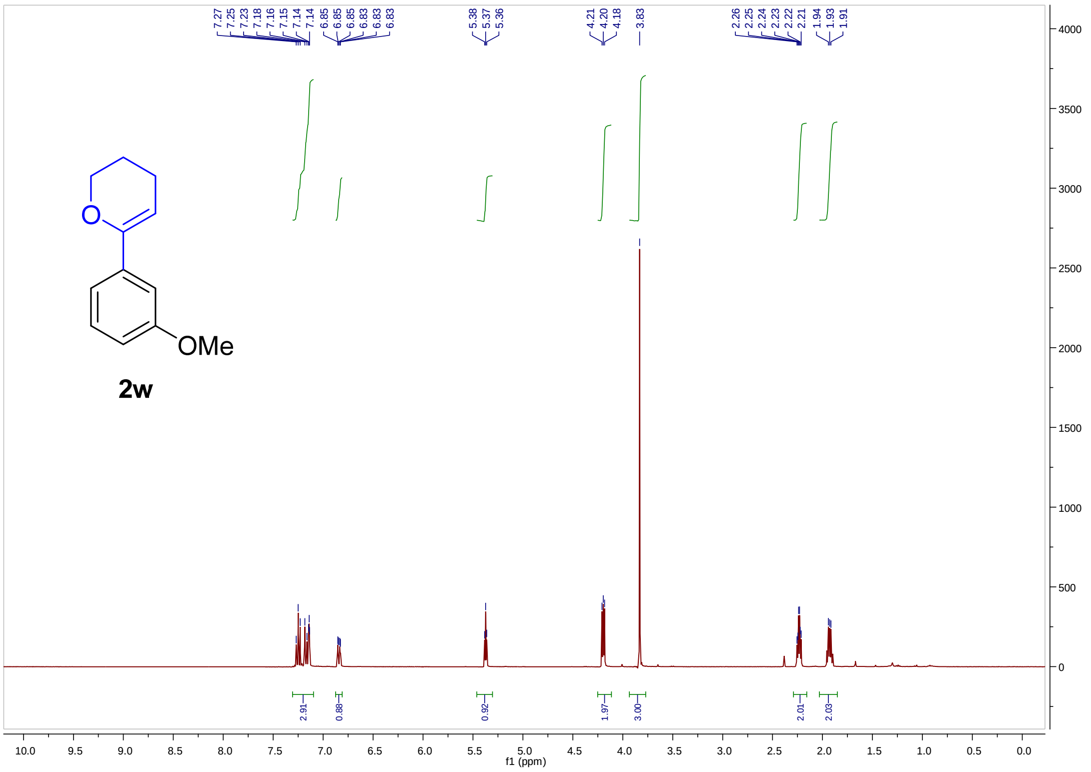
7.27 7.25 7.23 7.18 7.16 7.15 7.14 7.14 6.85 6.85 6.85 6.83 6.83 6.83
5.38 5.37 5.36
4.21 4.20 4.18 3.83
2.26 2.25 2.24 2.23 2.22 2.21 1.94 1.93 1.91
O
OMe
2w
2.91 0.88 0.92 1.97 3.00 2.01 2.03
f1 (ppm)

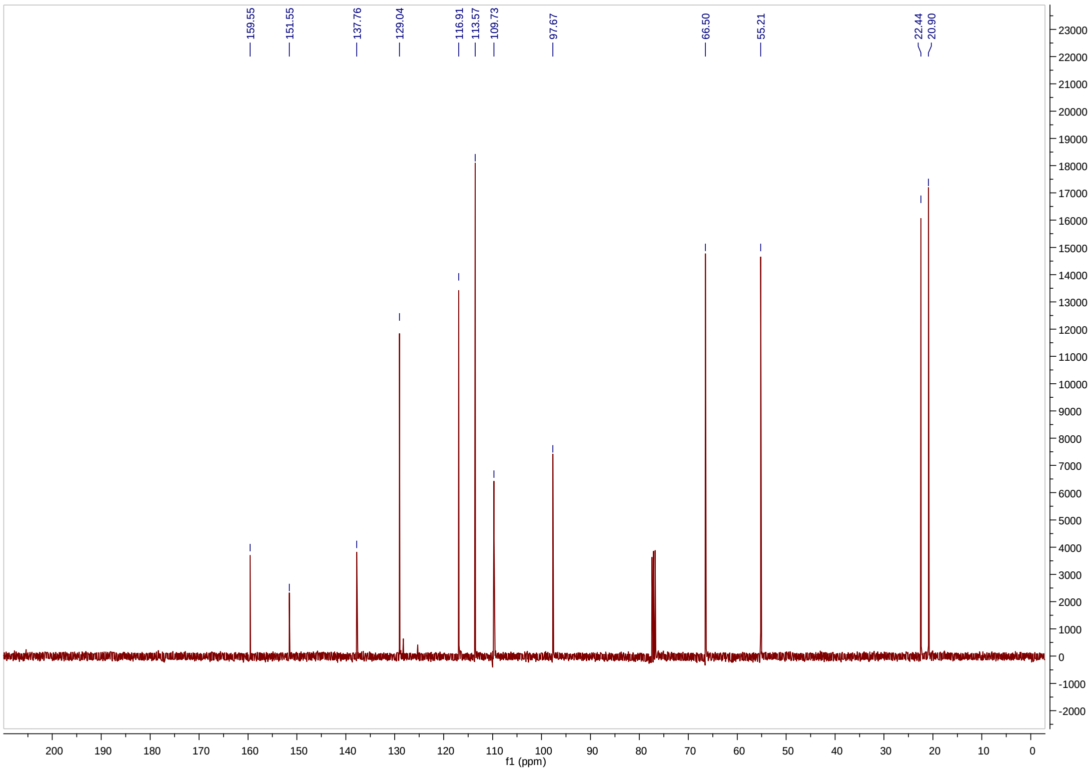
159.55 151.55 137.76 129.04 116.91 113.57 109.73 97.67 66.50 55.21 22.44 20.90
f1 (ppm)

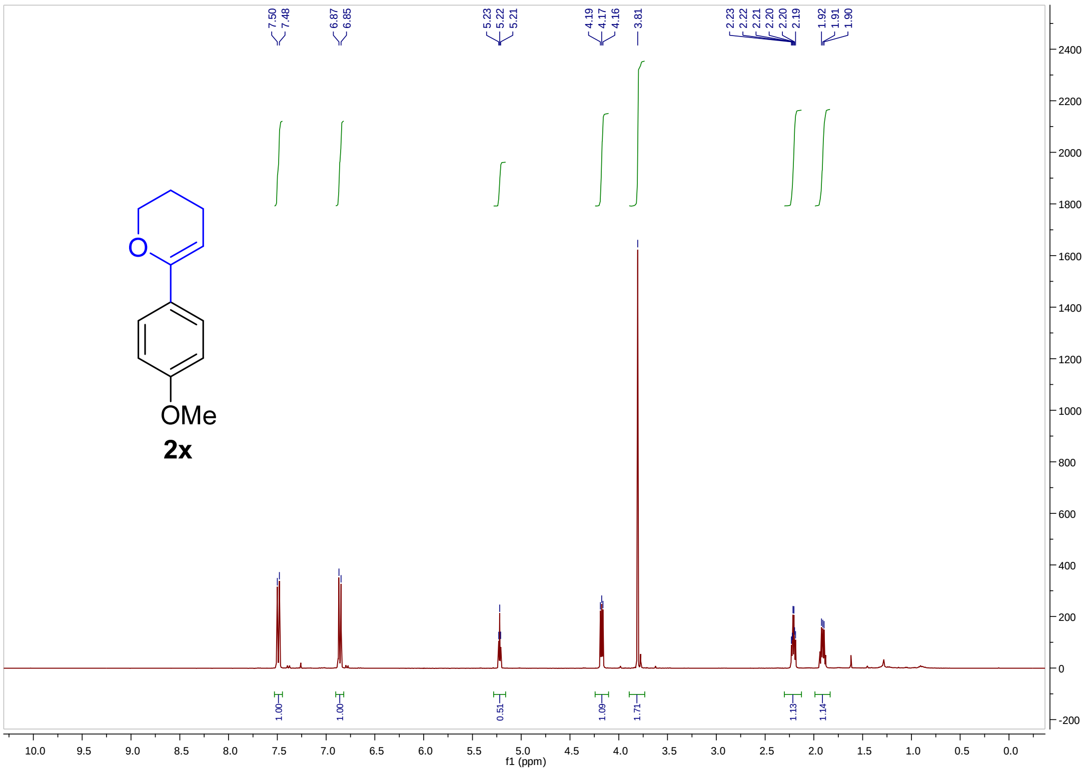
7.50
7.48
6.87
6.85
5.23
5.22
5.21
4.19
4.17
4.16
3.81
2.23
2.22
2.21
2.20
2.20
2.19
1.92
1.91
1.90
OMe
2x
1.00
1.00
0.51
1.09
1.71
1.13
1.14
f1 (ppm)

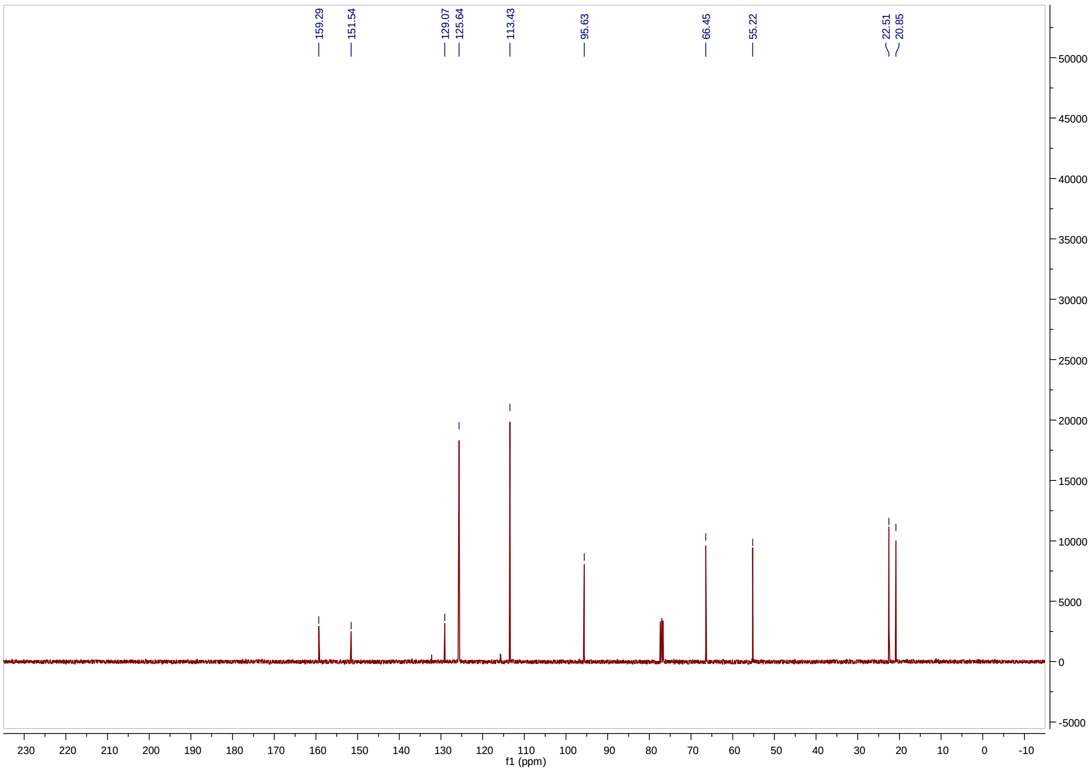
159.29
151.54
129.07
125.64
113.43
95.63
66.45
55.22
22.51
20.85
f1 (ppm)

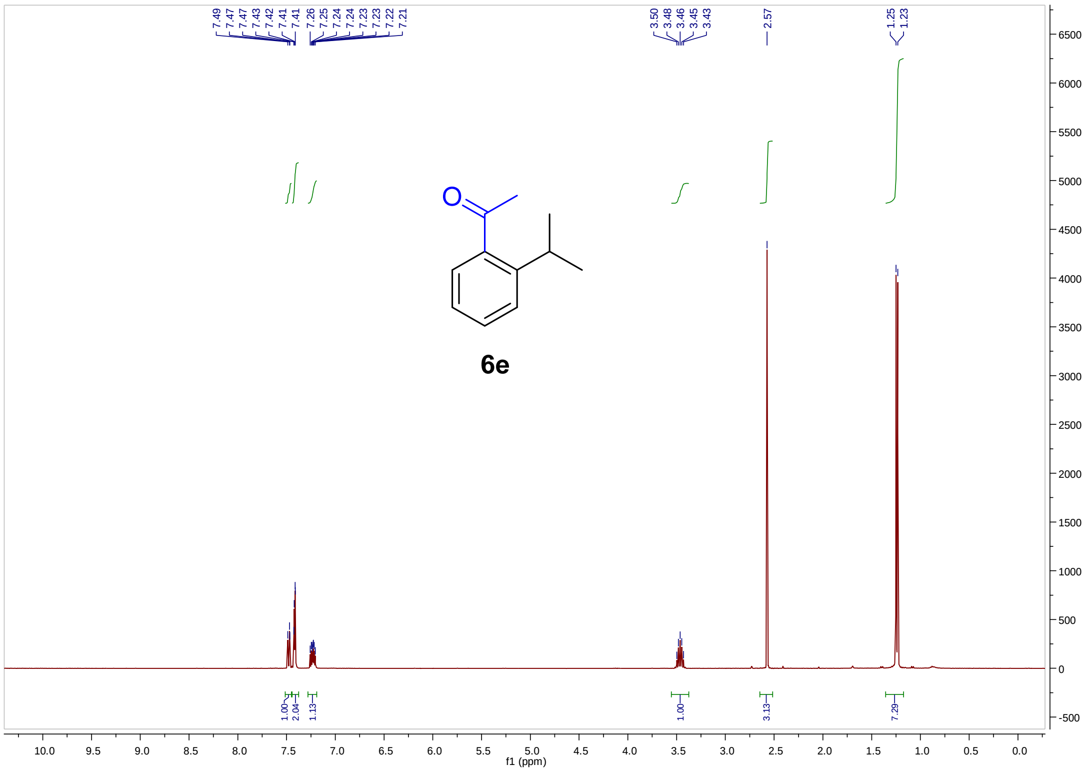
7.49
7.47
7.47
7.43
7.42
7.41
7.41
7.26
7.25
7.24
7.24
7.23
7.23
7.22
7.21
3.50
3.48
3.46
3.45
3.43
2.57
1.25
1.23
6e
1.00
2.04
1.13
1.00
3.13
7.29
f1 (ppm)

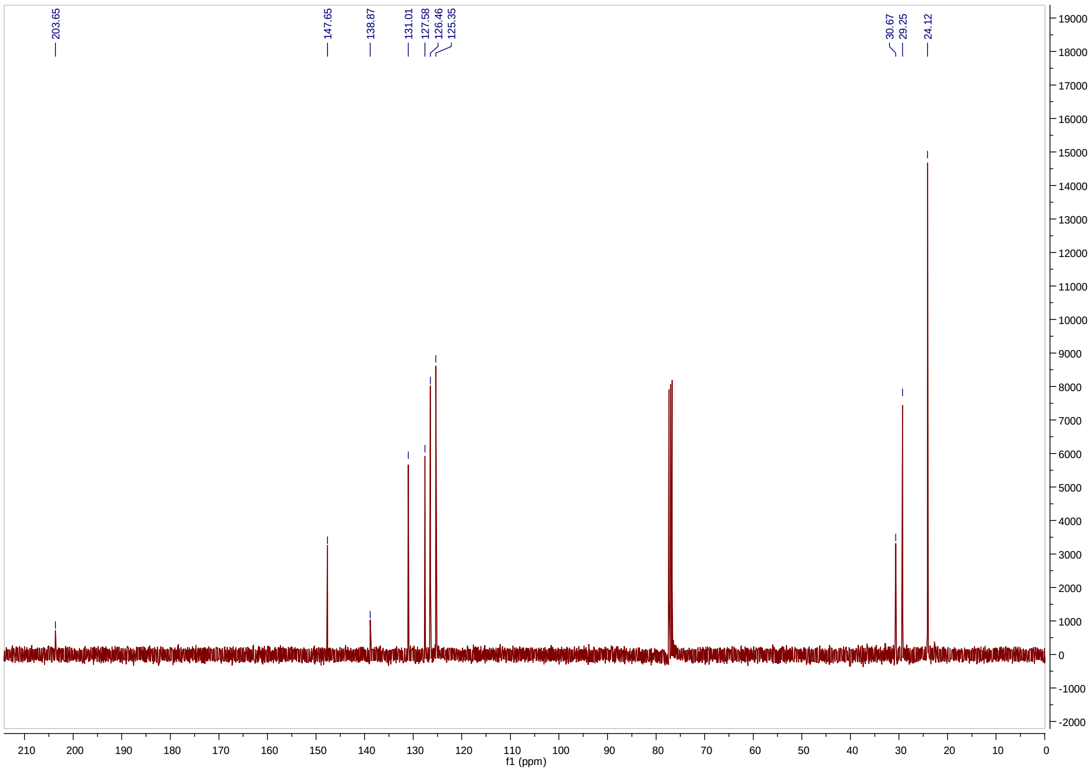
203.65
147.65
138.87
131.01
127.58
126.46
125.35
30.67
29.25
24.12
f1 (ppm)

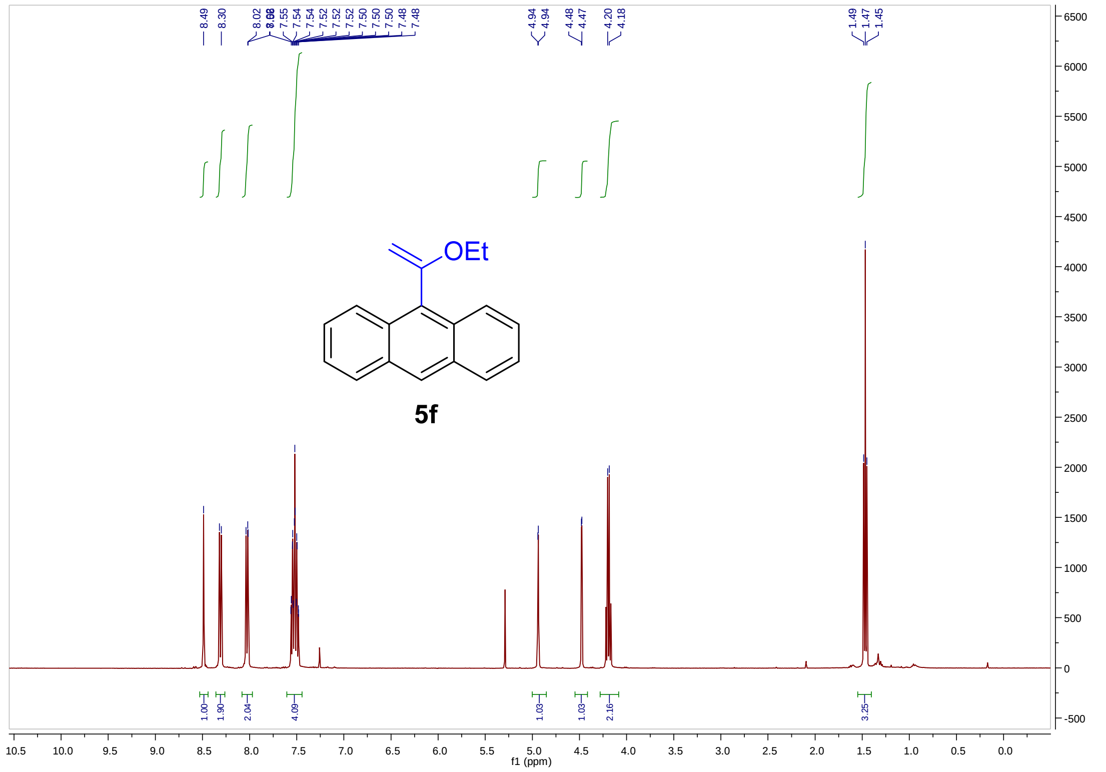
OEt
5f
8.49
8.30
8.02
7.55
7.54
7.54
7.52
7.52
7.52
7.50
7.50
7.50
7.48
7.48
4.94
4.94
4.48
4.47
4.20
4.18
1.49
1.47
1.45
1.00
1.90
2.04
4.09
1.03
1.03
2.16
3.25
f1 (ppm)

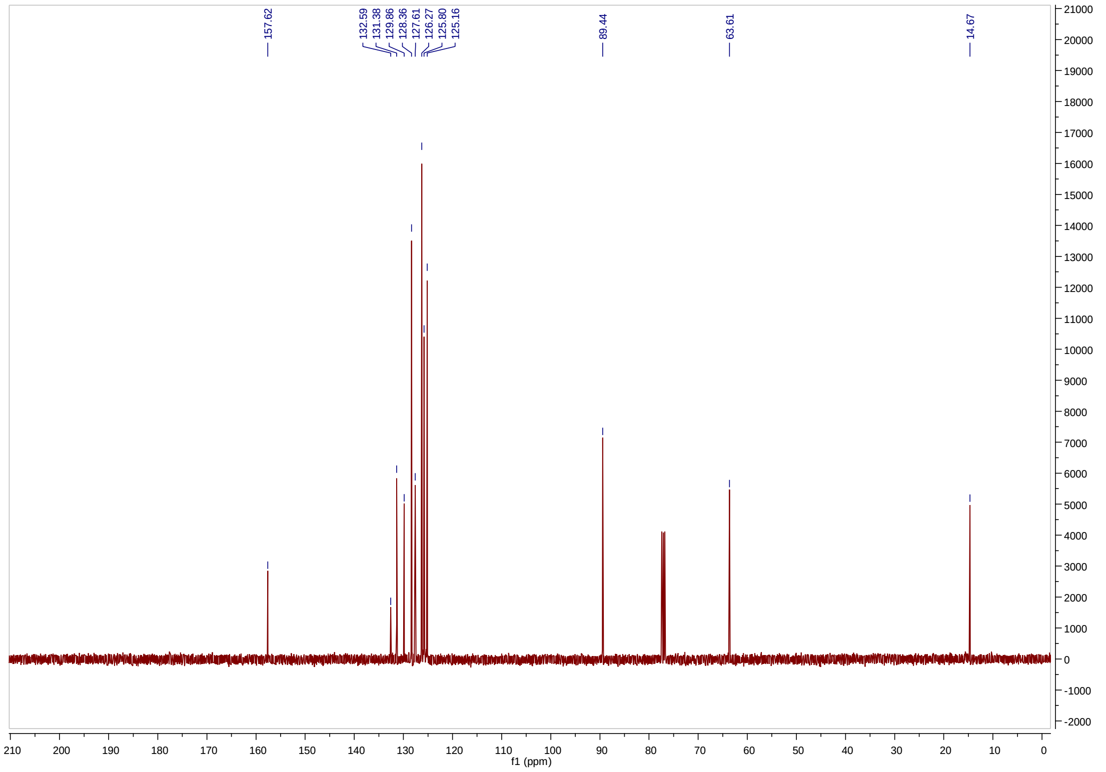
157.62
132.59
131.38
129.86
128.36
127.61
126.27
125.80
125.16
89.44
63.61
14.67
f1 (ppm)

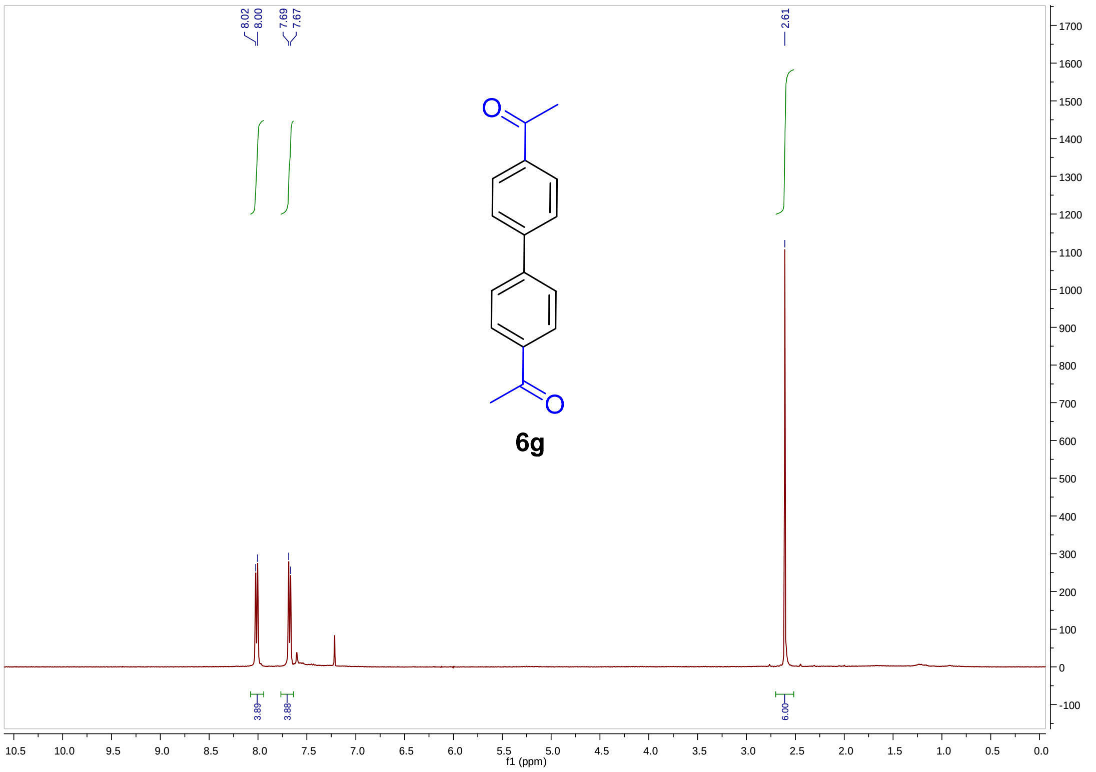
8.02
8.00
7.69
7.67
2.61
6g
3.89
3.88
6.00
f1 (ppm)

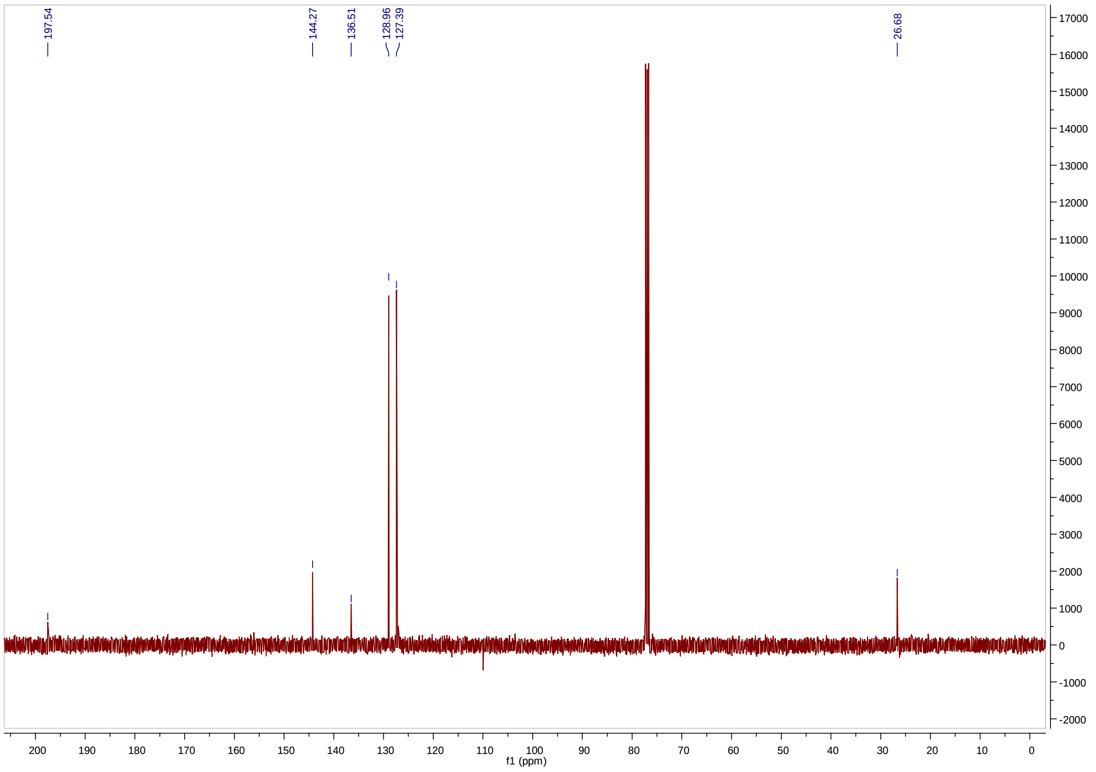
197.54
144.27
136.51
128.96
127.39
26.68
f1 (ppm)

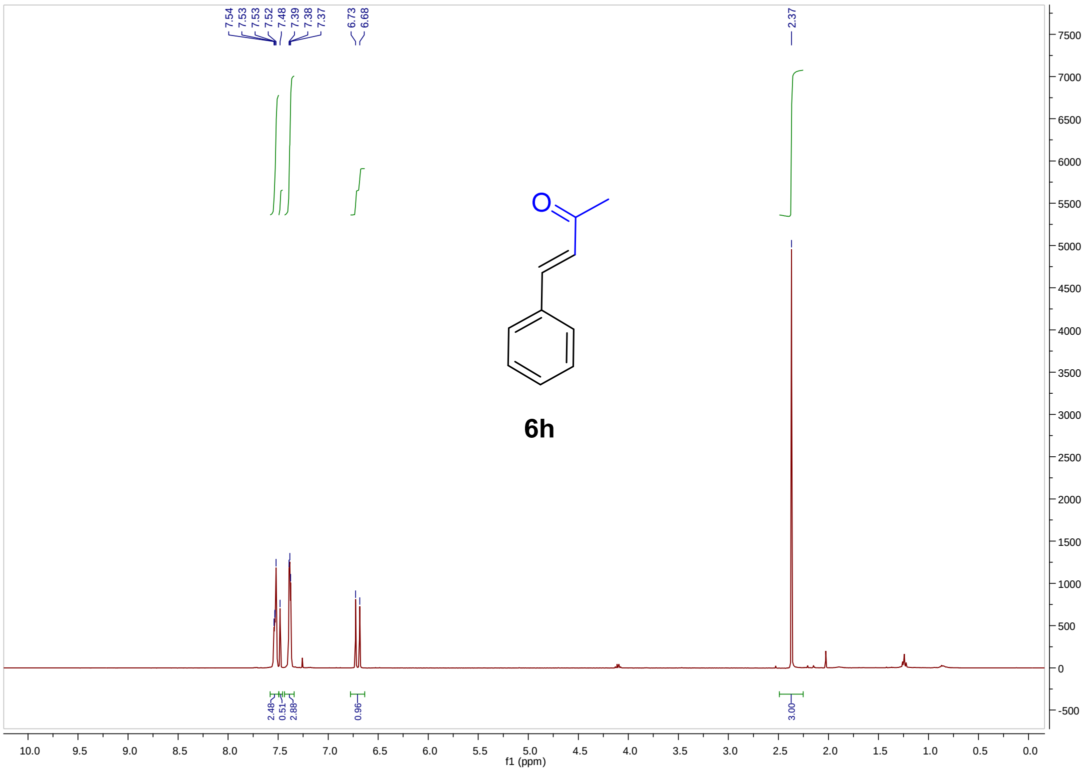
7.54
7.53
7.53
7.52
7.48
7.39
7.38
7.37
6.73
6.68
2.37
O
6h
2.48
0.51
2.88
0.96
3.00
f1 (ppm)

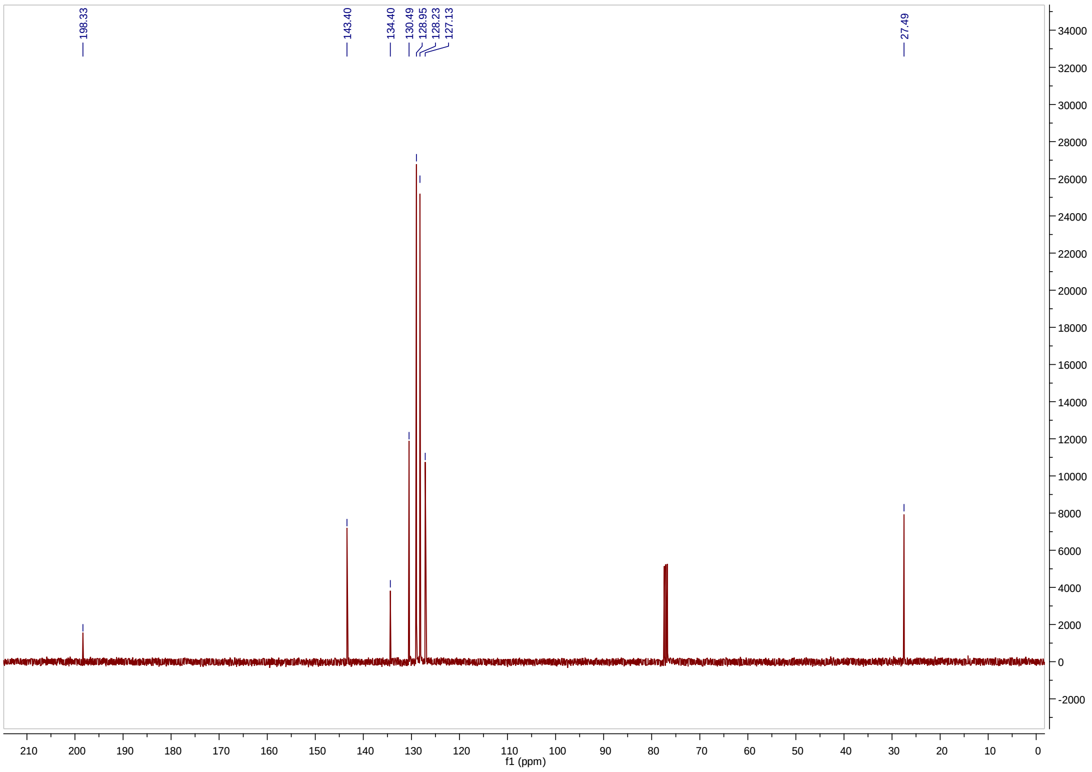
198.33
143.40
134.40
130.49
128.95
128.23
127.13
27.49
f1 (ppm)

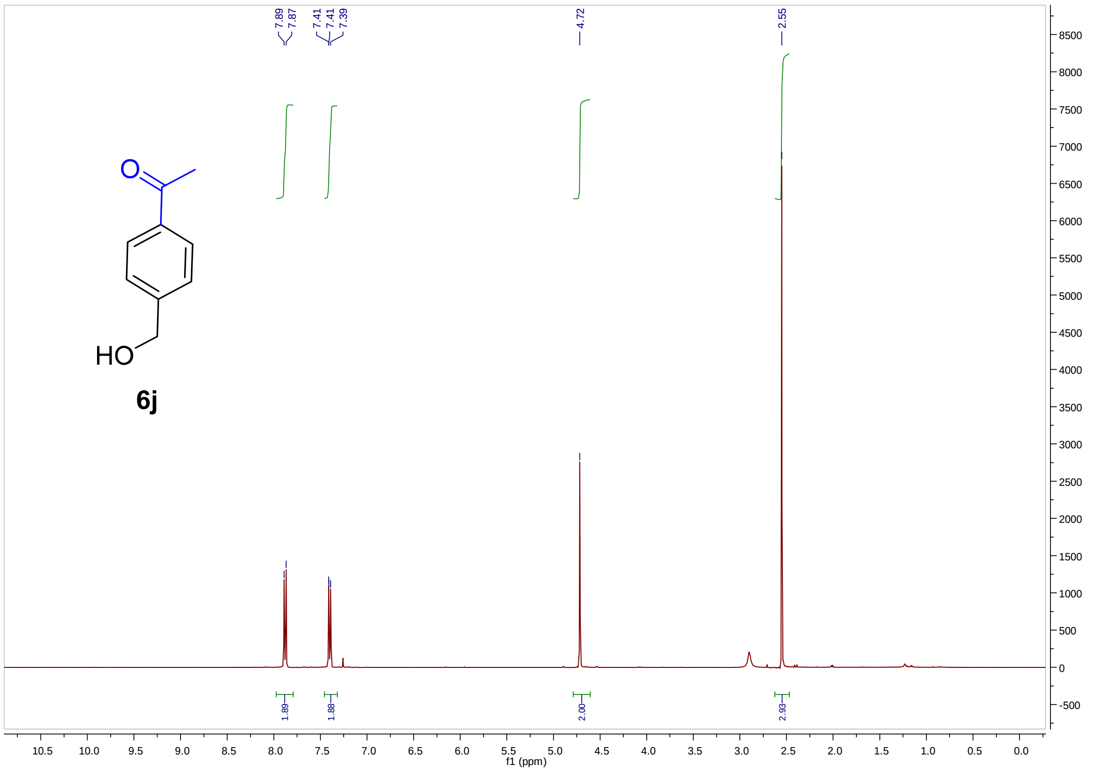
7.89
7.87
7.41
7.41
7.39
4.72
2.55
O
HO
6j
1.89
1.88
2.00
2.93
f1 (ppm)

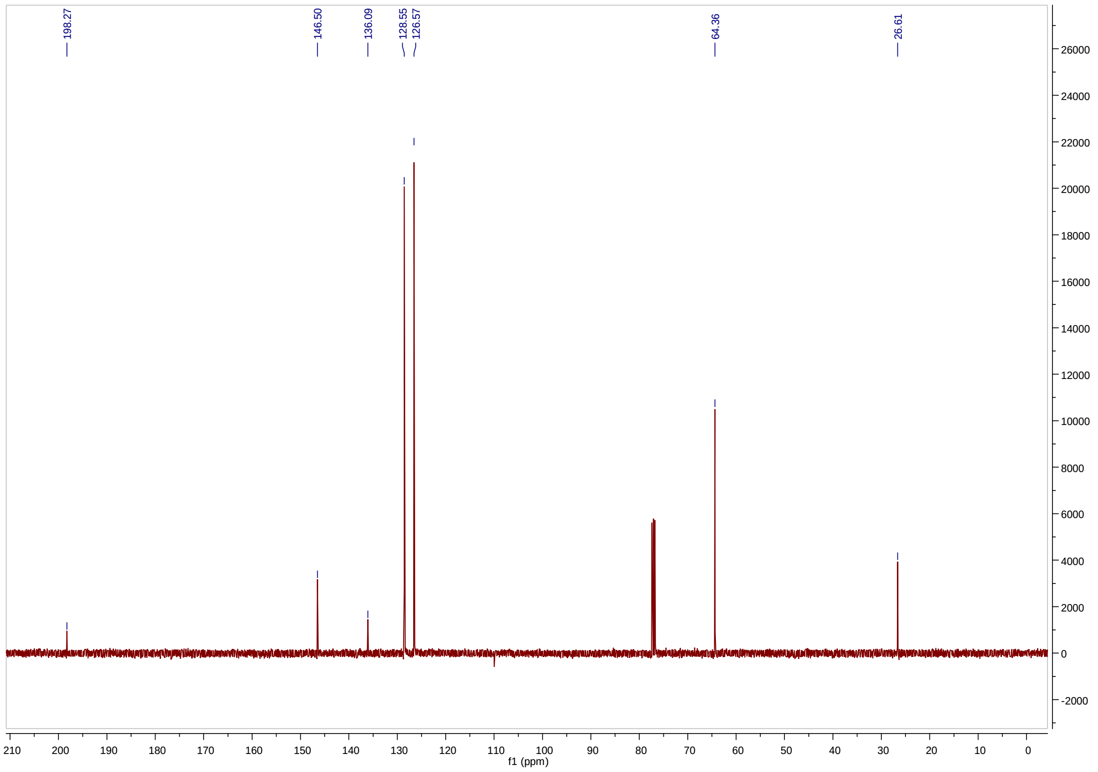
198.27
146.50
136.09
128.55
126.57
64.36
26.61
f1 (ppm)
